# Establishing the international prevalence of self-reported child maltreatment: a systematic review by maltreatment type and gender

**DOI:** 10.1186/s12889-018-6044-y

**Published:** 2018-10-10

**Authors:** Gwenllian Moody, Rebecca Cannings-John, Kerenza Hood, Alison Kemp, Michael Robling

**Affiliations:** 1 Centre for Trials Research, Cardiff University, Neuadd Meirionnydd, Heath Park, Cardiff, Wales, UK; 2 School of Medicine, Department of Population Medicine, Cardiff University, Neuadd Meirionnydd, Heath Park, Cardiff, Wales, UK

**Keywords:** Child maltreatment, Prevalence, Self-report, Systematic review

## Abstract

**Background:**

Estimating the prevalence of child maltreatment is challenging due to the absence of a clear ‘gold standard’ as to what constitutes maltreatment. This systematic review aims to review studies using self-report maltreatment to capture prevalence rates worldwide.

**Methods:**

PubMed, Ovid SP and grey literature from the NSPCC, UNICEF, The UK Government, and WHO from 2000 to 2017 were searched. The literature review focused on the variation found in self-reported lifetime prevalence for each type of maltreatment between studies by continent and gender, and how methodological differences may explain differences found.

**Results:**

Sexual abuse is the most commonly studied form of maltreatment across the world with median (25th to 75th centile) prevalence of 20.4% (13.2% to 33.6%) and 28.8% (17.0% to 40.2%) in North American and Australian girls respectively, with lower rates generally for boys. Rates of physical abuse were more similar across genders apart from in Europe, which were 12.0% (6.9% to 23.0%) and 27.0% (7.0% to 43.0%) for girls and boys respectively, and often very high in some continents, for example, 50.8% (36.0% to 73.8%) and 60.2% (43.0% to 84.9%) for girls and boys respectively in Africa. Median rates of emotional abuse were nearly double for girls than boys in North America (28.4% vs 13.8% respectively) and Europe (12.9% vs 6.2% respectively) but more similar across genders groups elsewhere. Median rates of neglect were highest in Africa (girls: 41.8%, boys: 39.1%) and South America (girls: 54.8%, boys: 56.7%) but were based on few studies in total, whereas in the two continents with the highest number of studies, median rates differed between girls (40.5%) and boys (16.6%) in North America but were similar in Asia (girls: 26.3%, boys: 23.8%).

**Conclusions:**

Median prevalence rates differ substantially by maltreatment category, gender and by continent. The number of studies and available data also varies and relatively little is known about prevalence for some forms of maltreatment, particularly outside of the North American context. Prevalence rates require caution in interpretation as some variation will reflect methodological differences, including the data collection methods, and how the maltreatment is defined.

**Electronic supplementary material:**

The online version of this article (10.1186/s12889-018-6044-y) contains supplementary material, which is available to authorized users.

## Background

Nationally and internationally, there has been a growing recognition of the importance of identifying, documenting and reporting suspected and confirmed child maltreatment [1], with the World Health Organisation (WHO) in collaboration with the United Nations Children’s Fund (UNICEF) calling for maltreatment to be recognised as a global public health concern [2].

Having a clear definition of child maltreatment is recognised as fundamental [3]. WHO has defined child maltreatment as ‘All forms of physical and/or emotional ill-treatment, sexual abuse, neglect or negligent treatment or commercial or other exploitation, resulting in actual or potential harm to the child’s health, survival, development or dignity in the context of a relationship of responsibility, trust or power’, with the clear realisation that the four categories may coexist in the same child [4]. There is some variation in the definitions of the different categories in the four countries in the United Kingdom (UK), and these differences can make comparisons difficult, however, all can nevertheless be classified as ‘maltreatment’ for the purposes of this review.

### Public sector collected data

Data routinely collected within the public sector which could shed light on the extent of child maltreatment in the UK and can be found from records of contacts with child protection services i.e. social services, and in offenses against children [5]. Data related to contacts with social services include the number of referrals accepted by social services, when a child is recorded as a ‘child in need’, (assessed under section 17 of the Children Act 1989, article 17 of the Children Order 1995, Section 12 of the Children (Scotland) Act 1995), and/or has suffered or is likely to suffer ‘significant harm’ (section 47 of the Children Act 1989, articles 2(2) and 50(3) of the Children Order 1995, Children (Scotland) Act 1995), and/or the child is the subject of a ‘child protection plan’ or on the ‘child protection register’, and when a child is being ‘looked after’. Data relating to the reasons a child is subject to a protection plan or on the child protection register are also collected, with neglect being the most common reason for this in each of the four UK countries [6]. The rate of children who are subject to a child protection plan has increased in all UK countries over recent years [6]. Statistics on offences against children recorded by the police include data on homicides and child deaths [6], as well as sexual, cruelty and neglect offences.

Cases of maltreatment that come to the attention of social services or the police are only a portion of the true numbers [7, 8]. There are many more that go undetected, unreported or unrecorded [9]. Fallon et al. (2010) likened this to the tip of the iceberg [10].

### Other sources of maltreatment data: Self-report

Gathering data on maltreatment using formally collected data only can be problematic because of the sole reliance on system indicators, created for bureaucratic and tracking purposes as opposed to research purposes [11], although formally reported cases are likely to represent more serious episodes. Even when data are collected from several different organisations and combined, this is likely to be an underrepresentation [12], due to underreporting. Fallon et al. (2010) note that how a child maltreatment event is measured will affect counts of maltreatment cases by agencies. The number of children investigated for maltreatment may be hard to detect as this will depend on data collection and aggregation methods. For some agencies children investigated several times in a year may be counted each time as a separate investigation [10]. The area covered by the agency could also affect count; cases where children or families move between areas could be double-counted or missed altogether [10].

Formally collected data are especially likely to under represent child maltreatment in middle-andupper-income families [13], this may be due to agencies being less likely to intervene in these groups. Less is known about the prevalence of maltreatment that is not reported to social services or the police [5], however, many have conducted studies to attempt to capture the prevalence of maltreatment using self-reporting methods.

Formal estimations of the prevalence of child maltreatment based mostly on self-report have been conducted by other researchers. Barth et al. (2013) conducted a systematic review with a meta-analysis of the prevalence of child sexual abuse worldwide in studies published between 2002 and 2009. Fifty-five studies from 24 countries were included and prevalence estimates ranged from 3 to 31% [14]. Pereda et al. (2009b) conducted a meta-analysis of self-reported child sexual abuse in community and student samples worldwide. They included sixty-five articles covering 22 countries, and found that the mean prevalence was 7.9% for men and 19.7% for women [15]. Both of these studies included meta-analyses of data from studies of child sexual abuse only; the current review seeks to expand on this by including prevalence rates of physical, emotional/psychological abuse, and neglect. Stoltenborgh and colleagues have conducted meta-analyses of data from studies of that addressed the four types of maltreatment [17–19, 26], all of these included studies published up to 2008, the current systematic review expands on these works by reviewing more contemporary studies, and presenting studies on prevalence of the four different types of maltreatment in one review.

The aim of this current study is to establish prevalence rates for each category of self-reported maltreatment and how they may vary by gender and geography. How methodological differences may explain differences found in prevalence figures will be explored.

## Methods

### Literature review

A literature search took place between May and June 2014, and was updated in March 2017. Electronic literature databases (PubMed, OvidSP) as well as literature from other organisations (NSPCC, UK Government, WHO, UNICEF) were searched for potentially eligible studies and grey literature. The combined search strategy included terms for the population (children and young people), the incident (maltreatment) and various terms to convey ‘measurement’. Duplicate literature was removed using a standard de-duplication function in EndNote, titles and abstracts were reviewed. The detailed search strategy is included in Appendix 1.

### Study selection

The original search between May and June 2014 was conducted for a wider literature review, and included searching for all studies reporting the prevalence of ever experienced child maltreatment (under 18 years old) worldwide published from 2000 onwards, and therefore the search terms in Appendix 1 relate to this original search. Studies before 2000 were not included as the authors were interested in relatively contemporary data. The studies included in this review are more focused in that we have included only those that relate to *lifetime* prevalence of child maltreatment by *self-report*. Prevalence can be either the lifetime or period prevalence of child maltreatment. Lifetime prevalence is the number of individuals having experienced maltreatment at some point during childhood, with ‘childhood’ being defined in various ways depending on the paper or the country in question. Period prevalence is the number of individuals having experienced maltreatment at some point during a specified period of time, for example, the past year [10, 16]. It should be kept in mind that lifetime prevalence of childhood maltreatment would be contracted in some studies that include child self-report due to the children not having completed childhood which may be reduced due to lower time of exposure. For the purposes of this review therefore, ‘lifetime’ prevalence refers to true lifetime prevalence of child maltreatment as well as studies that include children and their lifetime prevalence to the point of self-report.

A reference list checking technique was used when ascertaining potential studies, i.e. where relevant studies were found using the search strategy, the reference lists of these studies were searched for other relevant publications.

Table 1 in additional file 1 details the inclusion and exclusion criteria applied to the literature.

Included in the search was any study where a participant (adult (18+) or a child (< 18)) self-reported lifetime child maltreatment before the age of 18 years. Study designs were methodologically restricted to the primary data collection (i.e. no routinely collected or secondary data sources). Excluded were any study restricting child maltreatment to a specific time reference period (e.g. in the past year) compared to entire 18 years of childhood and any study where a secondary person reports childhood maltreatment on behalf of the participant (e.g. parent).

Initial stage of review for inclusion: All titles and abstracts found were reviewed by a single reviewer. A random selection of 100 titles and abstracts were triple-screened against the inclusion/exclusion criteria by two additional reviewers.

Agreement for inclusion/exclusion between the three reviewers was ascertained using Fleiss’ Kappa [20], and agreement was very high at 0.97. Fleiss’ Kappa, as opposed to Cohen’s Kappa was used to as Fleiss’ Kappa should be used when there are more than two raters.

Final stage of review for inclusion: As reviewer agreement was high, full papers were retrieved for all selected abstracts and then screened again with more detailed inclusion criteria. Confirmation of inclusion was performed at this stage as this related to criteria that could usually only be ascertained with the whole paper.

### Data extraction

The following data were extracted from the included studies: Authors and year of publication, country, age and gender of participants, population, total number of participants in study, mode of self-reporting completion (self-completed, interview), type of maltreatment, description of maltreatment, and prevalence rates. Prevalence rates were recorded by type of maltreatment and split by gender where possible. Additional file 2 presents these data for each study included, and additional file 3 contains the references for these studies. An additional reviewer verified the data extraction for a random selection of 10 studies, the data extraction process was found to be satisfactory.

### Presentation of data

Box and whisker plots are presented to show the median (alongside 25th to 75th centiles and outliers) of lifetime prevalence of maltreatment by gender and geographical region (continent) for each of type of maltreatment (emotional/psychological abuse, neglect, sexual, physical) (Figs. 2 3, 4, and 5). Where a study reported results from more than one country we have represented prevalence rates from these countries separately where possible to do so. In two studies which involved countries politically within two continents (Turkey, Russia) we have categorised by continent based on the location of the majority of the study population (i.e. to Asia and Europe respectively). We have also generated separate prevalence rates for studies that involved separately self-reported maltreatment by adults and by children. Ranges of rates are presented rather than pooled prevalence due to the high level of heterogeneity variation observed. As this study was conducted as part of a larger body of work assessing maltreatment assessment and reporting in the UK, we have also presented data for UK studies only (Additional file 4).

Prevalence rates were apparently higher in some clinical samples compared to samples drawn from a general population. Therefore, for presentation purposes we have further presented the same figures showing rates for each type of maltreatment by gender and continent for general population samples only (Additional file 5). This excludes those sampled either due to specific socio-demographic or clinical characteristic (including specific professional groups) but has included those recruited from natural sampling frames such as schools, universities, broadly-based healthcare or primary care organisations and epidemiological cohorts (e.g. population-based pregnancy cohort).

The authors made the following assumptions and changes in order for data to be depicted in Figs. 2, 3, 4, and 5 in an orderly manner. Where prevalence figures were available for more than one country within a single study, we reported a prevalence rate for each separate country, he same was done for studies presenting separate self-reported prevalence rates for adult and child participants, these assumptions lead to there being a total of 343 ‘prevalence rates’ (within studies) relating to 337 studies For studies that reported on witnessing family violence, this was grouped under emotional/psychological abuse. As gender split for prevalence rates were unspecified in many of the studies, ‘male’, ‘female’ and ‘unspecified’ genders were included in the results. We defined the age of the victim of maltreatment to be 18 and under, however, it is important to note here that some studies included in this review specified a lower upper age limit.

## Results

**Fig. 1:**
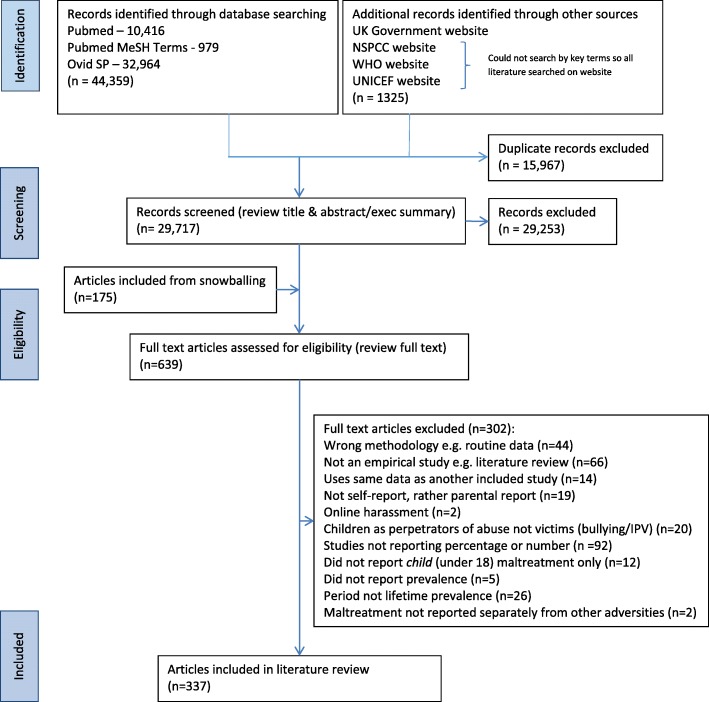
PRISMA flowchart depicting literature searched, included and excluded

Of the 44359 records identified through database searching and 1325 through additional sources, 15967 duplicates were removed and a further 29253 excluded at title and abstract stage (Fig. 1). A further 175 articles were identified through citation checking and 639 articles were assessed as full texts, of which 302 were excluded as not meeting eligibility criteria. A total of 337 articles were retained for inclusion.

There were more studies including retrospective reports from adults only (*n* = 216, 64.1%) (adults being defined as 18 or over), rather than children only (*n* = 28, 8.3%), and the remaining studies included self-reports of both adults and children (*n* = 93, 27.6%). The vast majority of studies used self-completed data collection (*n* = 213, 63.2%), the rest included data collected via interview (*n* = 120, 35.6%), and a very small number collected data via both interview *and* self-completion (*n* = 3, 0.9%), or interview *or* self-completion (n = 1, 0.3%).

**Fig. 2:**
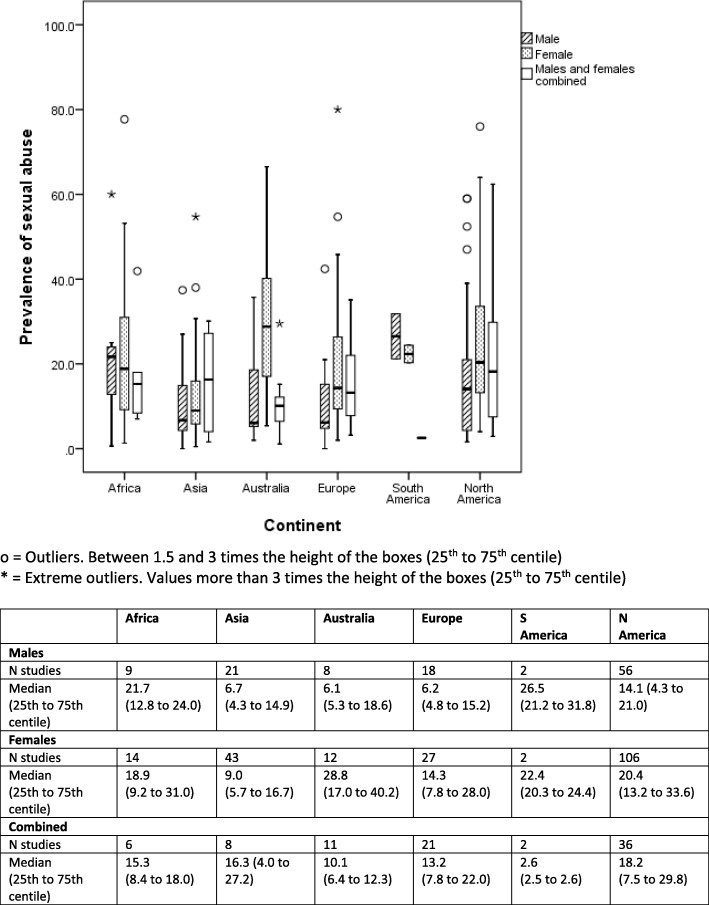
Self-reported lifetime prevalence of child sexual abuse (*n* = 287studies reporting 402 prevalence rates)

Figures 2 3, 4, and 5 show prevalence rates for each type of maltreatment. In addition, there were studies where form of maltreatment was not distinguished and these have been excluded from presentation. Approximately a third of all studies did not report the gender of participants (108, 32.0%), some studies included only female participants (*n* = 109, 32.3%), some had a mixture of males and females (*n* = 101, 30.8%), and a minority included males only (*n* = 17, 5.0%).

**Fig. 3:**
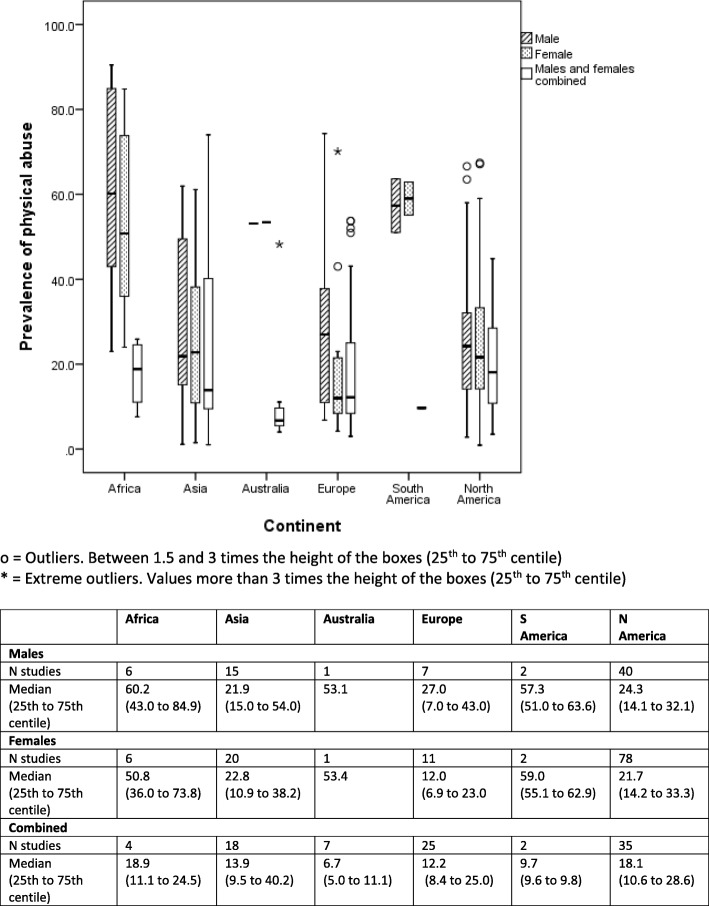
Self-reported lifetime prevalence of child physical abuse (*n* = 200 studies reporting 280 prevalence rates)

**Fig. 4:**
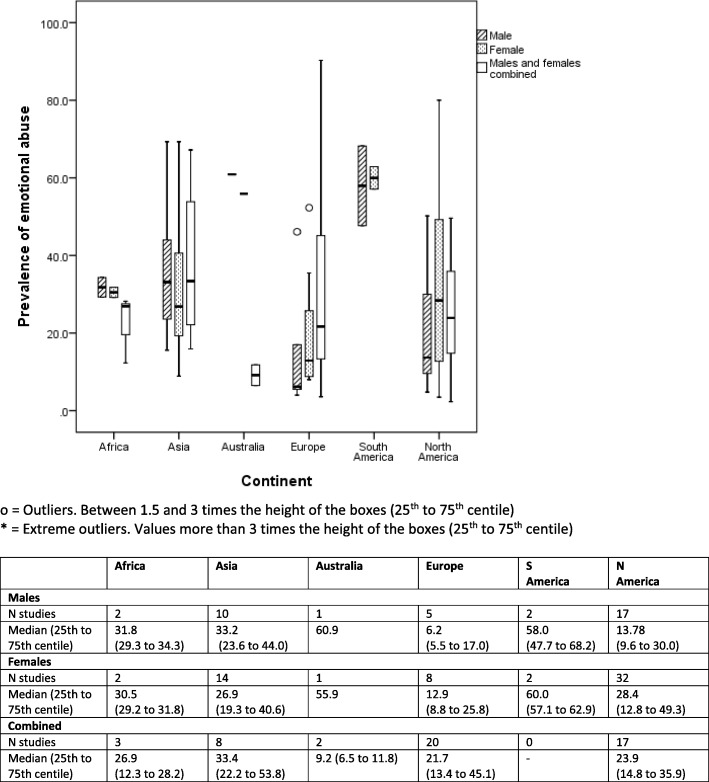
Self-reported lifetime prevalence of child emotional/psychological abuse (*n* = 105 studies reporting 146 prevalence rates)

**Fig. 5:**
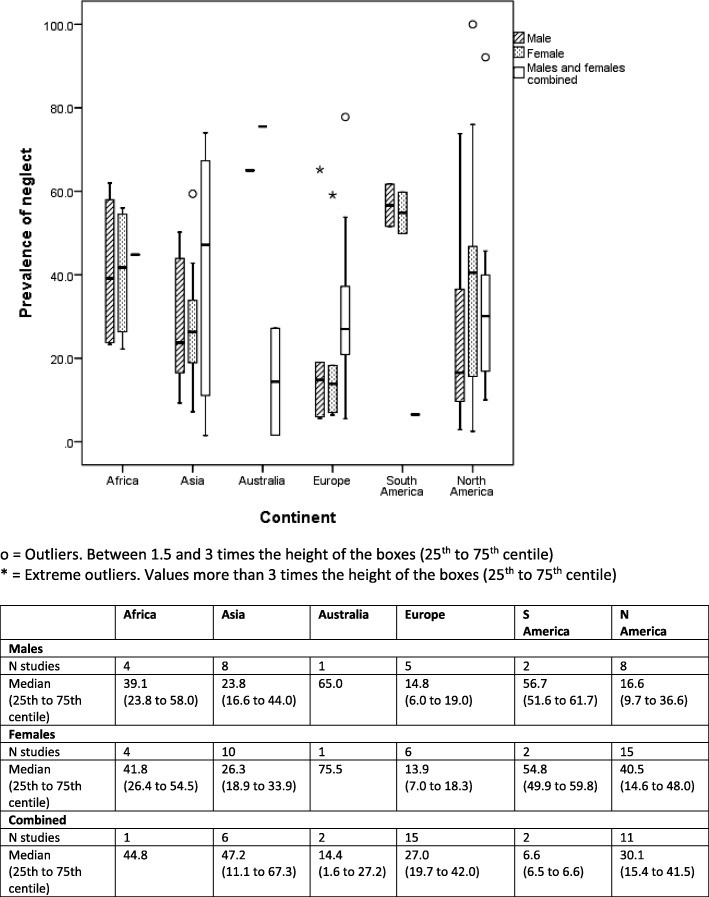
Self-reported lifetime prevalence of child neglect (*n* = 72 studies reporting 103 prevalence rates)

### Prevalence of sexual abuse

When assessing study samples, a single study may comprise separate combinations of continent and gender (i.e. one study may report data for four samples, boys and girls in two different countries). In this context the most commonly studied form of maltreatment was sexual abuse and half of all such study samples (171 of 337) were found in North America. The second largest set of study samples was found for Asia and in contrast the least in South America. Where gender was distinguished, prevalence rates were generally higher for female samples apart from South America (but which had only a small number of studies) and Asia. In the three continents with much higher numbers of studies (North America, Asia and Europe), median (25th to 75th centile) prevalence rates still varied considerably for girls: 20.4% (13.2% to 33.6%), 9.0% (5.7% to 16.7%) and 14.3% (7.8% to 28.0%) respectively and for boys: 14.1% (4.3% to 21.0%), 6.7% (4.3% to 14.9%) and 6.2% (4.8% to 15.2%) respectively. When excluding studies focusing on clinical / sub-group samples (additional file 5), median prevalence rates were generally similar apart for that for North American boys (median 6.5%, 25th to 75th centile, 4.0% to 16.0%).

### Prevalence of physical abuse

Median rates of physical abuse similarly varied across continent, especially in Africa, Australia and South America but these were based on a very small number of studies in each case. In North America, where most studies had been undertaken, median prevalence rates (25th to 75th centile) were similar for boys and girls at 24.3% (14.1% to 32.1%) and 21.7% (14.2% to 33.3%) respectively. Rates were similar (and for both genders) in Asia, which had the second highest number of studies. In European studies, physical abuse was much higher for boys (27.0%) than for girls (12.0%).

### Prevalence of emotional abuse

Studies of emotional abuse were less commonly found and only in North America and Asia were there more than ten studies for each gender category reported separately. Prevalence rates amongst girls (28.4%) in North America were twice that for boys (13.7%) although there were twice as many study samples for girls found. Prevalence rates in Europe were approximately half those reported in North America for both genders (boys: 6.2%, girls: 12.9%) and based on a smaller number of studies (boys *n* = 5, girls *n* = 8). In Asia, where there were more study samples involved median prevalence rates were higher for boys (33.2%) than for girls (26.9%). Prevalence rates elsewhere were high for both boys and girls but were based on a much smaller number of studies in each case. When reviewing non-clinical samples only, the rates of emotional abuse in North American girls was much lower (15.9%) but little different for boys (12.3%).

### Prevalence of neglect

There were fewer studies of neglect than for any other category of maltreatment, with North America providing the largest number for both boys (n = 8) and girls (*n* = 15). Prevalence rates were much higher for North American girls (40.5%) than for boys (16.6%). Prevalence rates in Asia were similar for boys (23.8%) and girls (26.3%), which was also the case in Europe but at a lower rate overall (boys: 14.8%, girls: 13.9%). There were only a very small number of studies across the remaining continents (Africa, Australia and South America) and prevalence rates were very high for each gender group.

### UK

There were 18 UK studies. Lifetime prevalence rates of self-reported maltreatment in childhood in UK literature varied considerably, prevalence of physical abuse ranged from 3.6% [21] to 32.6% [22]. Prevalence of sexual abuse ranged from 0.7% [9] to 27.8% [283. Prevalence of emotional or psychological abuse ranged from 4% [5] to 66.7% [23], and prevalence of neglect ranged from 5.6% [9] to 77.8% [23]. Finally, the prevalence of unspecified maltreatment ranged from 9.5% [24] to 48.4% [24].

## Discussion

We reviewed 337 study reports, which provided 343 prevalence rates, based on self-report from either adults or children. North American studies were most numerous across each category of abuse, whereas South American studies were least numerous. In approximately two-thirds of studies prevalence rates were available for either or both genders. Where differentiated, studies of girls were more common than for boys across all maltreatment categories. Prevalence rates were most commonly available for sexual abuse, then for physical abuse and least commonly for neglect. Median rates of sexual abuse were higher for girls than boys in the three continents with the highest number of studies (North America, Europe, Asia) and there were big differences between continents in actual rates (for example 20.4% and 14.3% for girls in North America and Europe respectively). Median rates of physical abuse were similar for boys and girls in all continents (for example 24.3% and 21.7% respectively in North America) apart from Europe and Africa where it was higher for boys (for example, 60.2 and 50.8 respectively for Africa, while rates varied considerably between continents for both girls and boys. Few studies of emotional abuse were found for Africa, Australia and South America and rates were much higher for girls than boys in North America and Europe but more similar in Asia (33.2% for boys, 26.9% for girls). Finally, a similar picture of study frequency was found for neglect and rates were much higher in North American girls (40.5%) compared to boys (16.6%) but similar across gender in both Europe and in Asia.

Pereda and colleagues [15] found substantial differences in prevalence of self-reported sexual abuse in their 2009 review of 65 studies. Their data suggested a ratio of 2.5 females for every one male victim. More recently, Stoltenborgh and colleagues [16] reported estimated prevalence for self-report studies of child sexual abuse in 2011 similarly across continents and by gender. They found gender made a substantial difference in difference in rates of self-reported abuse worldwide. While we did not statistically assess differences by gender, our findings bear that finding out. The paucity of studies in some geographical regions makes it more difficult to affirm such gender differences. The number of studies we retrieved where gender was not specified also confounds any potential differential effect of gender. The pattern of lower rates of sexual abuse Stoltenborgh found in Asia is also consistent with our findings, as was the highest rate of sexual abuse overall for Australian girls.

Considerable variation in lifetime prevalence rates of self-reported child maltreatment was found between studies, particularly between worldwide studies (between 0.0 and 100.0%), however, the variation in rates reported in UK based studies was still very large (between 0.7 and 77.8%). It is perhaps important to provide some context to the studies that reported the rather surprising extreme rates of 0.0% and 100.0%. Harkness and Monroe (2002) [25] found that all the females in their study reported that they had suffered neglect at some point, this was a clinical (depressed) sample, and so that may have had a bearing on the results. Khamis et al. (2000) [26] found that no males in their study had reported sex abuse, the respondents were boys aged 12–16 who were interviewed by school counsellors, it is possible that they therefore may have been reluctant to disclose a history of sex abuse due to discomfort or embarrassment. In both UK and worldwide studies the greatest difference in prevalence rates reported was for neglect. While some of this variation may reflect actual different experiences that children have, there are methodological differences that exist in the research that are likely to give rise to these variations [7, 9, 27]. We adopted a broad approach to inclusion for the review resulting in a heterogeneous sample of studies and prevalence rates.

### Study participants

The age of the participant at time of reporting may have an effect on prevalence rates. One of the most common methodological approaches for collecting maltreatment data involved the use of retrospective adult self-reports of childhood experiences [28]. Some researchers have raised concerns about the reliability and validity of retrospective recall in adult respondents, especially about childhood events and about events that are emotionally charged [29], what is known as recall bias [12, 30, 31]. Concerns include forgetting an experience that happened many years ago [32], while length of time since the abuse occurred may impact reliability [33], and adults maltreated as children may experience memory impairment related to the event [34]. Characteristics of the abuse may influence recall, including the type of abuse, the kinds of acts committed, or severity or chronicity of abuse [35]. It may be the case however that maltreatment is much more likely to be under-acknowledged rather than forgotten [36], and respondents may actively choose not to think about or disclose maltreatment experiences to avoid being reminded of them [37, 38].

Children are also asked to self-report maltreatment, and studies sometimes included both adults and children, and many of the methodological issues related to retrospective recall by adults can be problematic for children. Some researchers have been reluctant to question children directly about their experiences on account of ethical and procedural complications related to reporting requirements [39].

Comparison of prevalence rates from studies that collected self-reports from adults with those that involved children is problematic [11], for example, a study conducted in 2017 may include self-reported maltreatment as far back as the 1930s or 1940s for adults, but only as far back as the 1990s for children, the time lapse may have an effect, as well as social and legal changes in the definition and recognition of child maltreatment [36]. What individuals consider to be abusive behaviour may change between generations, for example, smacking a child was socially acceptable in the UK as recently as the 1980s [40], and still may be today. In principle however it may be possible to compare adult and child reports for time periods that coincide.

Gender of the participant may influence reporting, some evidence suggests that men may be less likely to reveal a history of maltreatment [33, 41]. The results of the current study seem to support this notion, particularly in relation to sexual abuse, however, the number of studies found concerning sexual abuse in men was relative low at 33% (115/345) compared to those concerning sexual abuse in women (56%, 195/345), it may be the case that there are true differences in prevalence rates between males and females [42]. It has been suggested that definitions of maltreatment do not capture the experiences of males adequately, specifically sexual abuse [15], or that fear of being labelled as weak or being flagged as homosexual might underestimate prevalence in males [43].

The population of study participants may affect prevalence rates [16], studies variously derived their samples from large samples of participants from the general population [9], clinical or service user samples, convenience samples such as university or college students, school pupils, or self-selecting volunteers. Prevalence estimates tended to be lower for samples drawn at random from general populations and convenience samples than those based on research with volunteers or service user samples [9, 43], for example Cawson et al. (2000) [44] found lower prevalence rates in all four types of maltreatment when using a population sample as compared to Fisher et al. (2011) who used individuals presenting to mental health services with psychosis [45]. University students may also be more aware of the study’s aims and thus more liable to response biases [16], while Goldman & Padayachi (2000) somewhat controversially suggested that university students may be a psychologically healthier group which may be associated with lower sexual abuse prevalence [43]. Drawing inferences from clinical samples can be problematic if the clinical setting from which the respondents are sampled is related to child protection intervention; it may be difficult to sort out causal order among the variables [11]. To demonstrate the impact that such variation can have on prevalence rates our additional figures showed results based on ‘non-clinical’ study samples. This did not always reduce the prevalence rates, although this was the general direction of effect. The study design, sampling framework adopted (for example, the application of staged and sub-group over-sampling) and the eligibility criteria applied could still exert a substantial effect of apparent prevalence rates even in non-clinical samples.

### Data collection mode

The measures used to collect data in self-report studies can be broadly divided into those that require the presence of a researcher presenting questions to a participant, and those that are self-administered. Method of data collection can artificially influence participant response, and some studies have shown that face-to-face interviews result in higher reporting rates compared to self-completed questionnaires [27]. Amodeo et al. (2006) found that the prevalence of sexual abuse in their sample was higher based on a combined questionnaire and interview rather than a questionnaire alone [46]. Face-to-face methods can also give opportunities for clarification and probe ambiguous responses, and remind participants of expectations for honesty [47, 48]. Face-to-face interviews have the advantage of allowing for greater rapport, participants may prefer this method [47], disclosure may be promoted [48] through understanding and support on the part of the interviewer while others have not reported such a difference [27]. It may also be the case however that interviewer presence may hamper disclosure if participants are reluctant to reveal sensitive information directly, may also cause participants to be more vulnerable to the effect of social desirability [11, 12]. Not everyone however, is equally prone to discomfort relating to sensitive questions, even at a young age [36].

### Definitions of child maltreatment

Participants’ ideas of what constitutes maltreatment can vary [5], and this may affect self-reported prevalence rates. Participants make a personal judgment about whether what took place was abusive if the questions asked are not specific [36, 49, 50]. Answers provided will therefore be influenced by participants’ subjective perceptions of abuse [16], which may be influenced by intergenerational changes in attitudes and cross-cultural differences, amongst other things. Previous studies have found that many people do not perceive childhood experiences such as ‘being whipped or beaten to the point of laceration’ as maltreatment, and there is a tendency to believe that discipline experienced as a child was normal [51, 52]. This however, should not affect responses to descriptive questions [5]; direct and specific questions tend to be used in validated measures, and are tested for internal consistency and pre-test reliability [9]. Age-appropriate questions that give behavioural descriptions of events help respondents to think about specific incidents and are preferred over questions that use legal terminology or ask respondents to label themselves as maltreated [53], and some have found that using broad questions are associated with lower prevalence rates of sexual abuse than more specific questions [54]. Furthermore, both the context and the number of questions asked can affect number of reports [27].

Some researchers specified an age range when asking participants about their maltreatment experiences, Bebbington et al. (2011) defined child sexual abuse as occurring before the age of 16 [36], and some did not. Diaz-Olavarrieta et al. (2001) asked participants if as a ‘child’ they experienced physical or sexual abuse [55], this may affect reported prevalence rates as one person’s idea of a ‘child’ may vary from another’s. When researchers defined child maltreatment as something that happens before the age of 16, those who were maltreated at ages 17 and 18 are missed. The definition of the perpetrator of the maltreatment may also affect prevalence rates, most studies do not specify details about the perpetrator, however, some focused narrowly on perpetrators as caregivers and family members, for example Annerbäck et al. (2010) [56]. It should also be noted that studies will under estimate infant and toddler abuse as the reporters may not be recall these events.

Some studies focused on one form of abuse, 34% (114/339) of the studies reviewed in this paper focused on sexual abuse only, with 56% (189/339) including more than one form of maltreatment. Although Bentley et al. (2017) reported that neglect was the most common reason for a child being subject to a protection plan or on the child protection register in the four UK countries [6], a disproportionate amount of studies have been conducted on the prevalence of sexual and physical abuse. Perhaps this is a reflection of perceived or actual seriousness of the various types of abuse, or possibly the understanding of what emotional abuse is or thresholds for neglect and whether neglect is always physical neglect or emotional neglect. The definitions used to assess the prevalence of abuse and neglect vary greatly between studies, and this may affect prevalence rates [30]. Radford et al. (2011) asked participants a series of very specific questions about experiences they may have had as a child [9], whereas Diaz-Olavarrieta et al. (2001) simply asked participants if they had experienced persistent physical/sexual abuse as a child [55], allowing participants to impose their own definition of abuse. Most studies, such as that by Diaz-Olavarrieta et al. (2001) [55] do not present their maltreatment definitions in enough detail in published papers [10].

Pereda et al. (2009) noted differences in definitions of what constitutes sexual abuse, including the age difference between the perpetrator and the victim, the age used to define childhood, and the type of sexual abuse [27]. Edgardh and Ormstad (2000) [57] and McCrann et al. (2006) [58] defined sexual abuse as when the perpetrator was at least five years older than the victim, this is often done to rule out sexual activity among peers [16]. There are also cultural and legal differences between countries in the age of consent to sexual intercourse which affects definitions [44]. The acts that constitute sexual abuse are a crucial part of a definition and would almost certainly affect prevalence rates, for example non-contact abuse such as exhibitionism can be more commonplace and may yield higher prevalence rates than contact abuse only [16].

Definitions of physical abuse may suffer from cultural preconceptions. As previously mentioned smacking is still legal in the UK but outlawed in some parts of Europe [40]. In spite of this, often too much is made of cultural differences, and there is a general consensus in many cultures about what constitutes maltreatment [40], cultural differences may therefore only play a small role in differences in reported rates of maltreatment.

Definitions of neglect vary greatly because recognition of neglect can be difficult; children who are victims of neglect experience multiple types of neglect and it is mostly persistent and rarely traceable to a single incident [59]. Definitions of neglect have been criticised for imposing middle-class values on lower-class families [60], and that they do not take cultural differences into account [59]. There has been debate on whether the focus of the definition should be around either caregiver behaviours, or of the experiences of the child, regardless of who is to blame [11]. Risk and protective factors can change with age and developmental ability; this can affect definitions [11]. Some researchers have purported that definitions of neglect should consider the frequency, duration, and severity of the neglect, the age of the child, and potential consequences to the child’s development [59, 61, 62]. Tonmyr et al. (2011) noted that emotional or psychological abuse can also have particularly ambiguous definitions [63].

Some forms of maltreatment overlap, for example, sexual abuse often also involves physical abuse, and all forms of maltreatment include an element of emotional or psychological abuse, this can complicate definitions [44].

Some of the reasons for differing prevalence rates described above are expected, for example, it’s unsurprising that there are variations in self-reports of different types of abuse and neglect, these expected reasons are less likely to represent error. Some of the differences in prevalence rates found however are more likely to represent error, for example, whether data collection is self-administered or requires the presence of an interviewer.

### Strengths and limitations

We have reviewed the literature and collated data on the lifetime prevalence of self-reported child maltreatment worldwide. PubMed, Ovid SP and grey literature from the NSPCC, UNICEF, The UK Government, and WHO from 2000 to 2017 were searched. These databases were selected as they were thought to likely contain literature on the prevalence of child maltreatment, and indeed yielded a large amount of articles on the subject. The authors recognise however that it is possible that other databases not utilised could have yielded additional papers. Literature that were not in the English language were excluded, this was due to budget restriction on translation work as this review was part of a PhD. All four types of child maltreatment were included in this review, and studies which did not specify the type were also included. Including all types of child maltreatment in the same review has not been done for some time and this is a strength of the current piece of work. For some studies no upper age limit was provided, contacting the authors of these papers was not justifiable given the current resources and so the authors assumed the upper age limit of 100 for those studies. The authors planned to conduct a meta-analysis on the prevalence reported rates however, studies varied considerably in the data they collected, the tools to collect the data, and the populations included. It was therefore not possible to form sufficiently large groups to warrant a meta-analysis.

Although a portion of all titles and abstracts were triple-screened against the inclusion/exclusion criteria by three additional reviewers, just a single reviewer was responsible for reviewing all the other abstracts, however, reviewer agreement was very high and so we believe that the review process was completed systematically.

## Conclusions

This review focused on the lifetime prevalence rates of maltreatment observed through respondent self-report. We found differences by gender and geography which are broadly consistent with previous reviews of child sexual abuse. In addition, we have expanded the focus to include other categories of maltreatment. The different number of studies across categories of maltreatment and across settings makes it harder to have similar levels of confidence about summary rates of prevalence, especially in Africa and South America. The lack of distinction by gender in many studies is concerning given the sizeable differences observed here and in previous reviews between boys and girls. Methodological differences between the studies may go some way towards explaining the differences found in prevalence rates. Methods and techniques for collecting data about experiences of maltreatment have advanced in recent years [9], and further research is required to optimise use of data from a variety of sources.

Recommendations for future work include, given the range of methodological differences in studies observed, that researchers may need to be more precise when selecting studies to include in a review such as this one, for example, by excluding studies that have used broad, non-specific labels of maltreatment which require a high degree of interpretation by the respondent. This may be a way to arrive at more useful rates of child maltreatment which will allow better comparisons between studies.

### Additional files

Additional file 1: Inclusion and exclusion criteria applied to the literature. (DOCX 12 kb)

Additional file 2: Table containing data for each of the studies included in review. (DOCX 73 kb)

Additional file 3: References for table (additional file 2) containing data for each of the studies included in review. (DOCX 47 kb)

Additional file 4: Prevalence of abuse by type and population. (DOCX 28 kb)

Additional file 5: Prevalence of maltretment by continent and gender - non-clinical samples only. (DOCX 81 kb)

## Appendix 1: Search strategy

### Search terms defined

Maltreatment to include (HM Gov, 2013):

Physical abuse: ‘A form of abuse which may involve hitting, shaking, throwing, poisoning, burning or scalding, drowning, suffocating or otherwise causing physical harm to a child. Physical harm may also be caused when a parent or carer fabricates the symptoms of, or deliberately induces, illness in a child’.

Emotional abuse: ‘The persistent emotional maltreatment of a child such as to cause severe and persistent adverse effects on the child’s emotional development’.

Sexual abuse: ‘Involves forcing or enticing a child or young person to take part in sexual activities, not necessarily involving a high level of violence, whether or not the child is aware of what is happening’.

Neglect: ‘The persistent failure to meet a child’s basic physical and/or psychological needs’.

*Lifetime prevalence and period prevalence* (Fallon et al, 2010; Stoltenborgh et al 2011):

Lifetime prevalence of maltreatment: the number of individuals having experienced maltreatment at some point during childhood.

Period prevalence of child maltreatment: the number of individuals having experienced maltreatment at some point during a specified period of time, for example, the past year.

### Search terms list – keywords

measur*

quantify*

comput*

estimat*

evaluat*

assess*

confirm*

child*

young pe* (people/person)

maltreat*

abuse*

neglect*

### Medical Subject heading (MeSH) Terms

abuse, child (MeSH)

grouped search terms

(measur* OR quantify* OR comput* OR estimat* OR evaluat* OR assess* OR confirm*) AND (maltreat* OR abuse* OR neglect*) AND (child* OR young pe*)

### Literature sources

- published research literature from the following databases:

http://www.ncbi.nlm.nih.gov/pubmed

www.thecochranelibrary.com

wok.mimas.ac.uk (Web of Science)

OvidSP (PsychInfo from 2002 only and Medline)

- policy and practice literature – UK Government specifically:

https://www.gov.uk/government/publications

- Charity publications – NSPCC, Action for Children:
  http://www.nspcc.org.uk/Inform/publications/
  www.actionforchildren.org.uk/policy-research/publications-and-briefings
- Use Web of Science or Google scholar to search for citations of articles and by authors important in the field:

scholar.google.co.uk

- Cardiff Child Protection Systematic Reviews:

http://www.core-info.cardiff.ac.uk/

### Inclusion and exclusion criteria:

**Table:**

Inclusion	Exclusion
Initial stage	
Child maltreatment (sexual, physical, emotional/psychological abuse and neglect)	
Lifetime prevalence	Period prevalence
Self-report	Data collected through routine sources or proxy report only (e.g. parents report)
English language	Not English language (literature was not translated as this was part of a PhD an included budget restrictions)
Systematic reviews as well as individual studies	Anything that is not a study or does not direct the reader to other studies
Maltreatment occurred when victim was under 18	Maltreatment occurred when victim was over 18
Published from 01/01/2000 onwards	Before 01/01/2000
Final stage	
As above in intitial stage	As above in initial stage
	Between-peer maltreatment such as bullying and teen partner abuse
	Studies that did not report either a percentage or a number (where percentage could be derived) of the prevalence of child maltreatment

### Search strategy

**Table:**

Search database/website	Search terms used	Date search performed	Number of returns
PubMed	((measur*[Title/Abstract] OR quantify*[Title/Abstract] OR comput*[Title/Abstract] OR estimat*[Title/Abstract] OR evaluat*[Title/Abstract] OR assess*[Title/Abstract] OR confirm*[Title/Abstract]) AND (maltreat*[Title/Abstract] OR abuse*[Title/Abstract] OR neglect*[Title/Abstract]) AND (child*[Title/Abstract] OR young pe*[Title/Abstract]))	1^st^ search: 01/01/2000 - 28/05/2014 2^nd^ search: 28/05/2014 - 15/03/2017	1^st^ search: 8532 2^nd^ search: 1884
PubMed MeSH terms	Child abuse/epidemiology [mh]	30/05/2014	979
Ovid SP	((measur* or quantify* or comput* or estimat* or evaluat* or assess* or confirm*) and (maltreat* or abuse* or neglect*) and (child* or young pe*)).tw.	1^st^ search: 01/01/2000 - 05/06/2014 2^nd^ search: 05/06/2014 - 15/03/2017	1^st^ search: 18401 2^nd^ search: 14563
NSPCC	Searched through all literature on website http://www.nspcc.org.uk/Inform/publications/	1^st^ search: 18/06/2014 2^nd^ search: 15/03/2017	N/A
UK Government	Searched through all literature on website https://www.gov.uk/government/publications using search terms ‘child abuse’	1^st^ search: 18/06/2014 2^nd^ search: 15/03/2017	N/A
WHO	Searched through all literature on website http://www.who.int/publications/en/	1^st^ search: 24/06/2014 2^nd^ search: 15/03/2017	N/A
UNICEF	Searched through all literature on website http://www.unicef.org/publications/	1^st^ search: 24/06/2014 2^nd^ search: 15/03/2017	N/A
