# Supplementary material for: Establishing the international prevalence of self-reported child maltreatment: a systematic review by maltreatment type and gender
Source: BMC Public Health. 2018 Oct 10;18:1164. doi: 10.1186/s12889-018-6044-y (PMC6180456; doi:10.1186/s12889-018-6044-y)
Supplement: Supplementary file 1 — Inclusion and exclusion criteria applied to the literature. (DOCX 12 kb) [file 12889_2018_6044_MOESM1_ESM.docx]

Additional file 1

**Table 1. Inclusion and exclusion criteria applied to the literature**

| **Inclusion** | **Exclusion** |
| --- | --- |
| **Initial stage** | |
| Child maltreatment (sexual, physical, emotional/psychological abuse and neglect) |  |
| Lifetime prevalence | Period prevalence |
| Self-report | Data collected through routine sources or proxy report only (e.g. parents report) |
| English language | Not English language |
| Systematic reviews as well as individual studies | Any publication that is not a study or does not direct the reader to other studies |
| Maltreatment occurred when victim was less than 18 years of age | Maltreatment occurred when victim was older than 18 years of age |
| Published from 01/01/2000 onwards | Before 01/01/2000 |
| **Final stage** | |
| As above in initial stage | As above in initial stage |
|  | Peer to -peer maltreatment such as bullying and teen partner abuse |
|  | Studies that did not report either a percentage or the frequency (where percentage could be derived) of child maltreatment |
