# Supplementary material for: Establishing the international prevalence of self-reported child maltreatment: a systematic review by maltreatment type and gender
Source: BMC Public Health. 2018 Oct 10;18:1164. doi: 10.1186/s12889-018-6044-y (PMC6180456; doi:10.1186/s12889-018-6044-y)
Supplement: Supplementary file 2 — Table containing data for each of the studies included in review. (DOCX 73 kb) [file 12889_2018_6044_MOESM2_ESM.docx]

**Additional file 2. Table containing data for each of the studies included in review**

| **Number** | **Author** | **Country** | **age as seen in paper** | **Age range inferred** | **Population** | **Total N** | **mode of completion** | **Type of maltreatment, gender, %** | **Maltreatment descriptions and calculations** |
| --- | --- | --- | --- | --- | --- | --- | --- | --- | --- |
| 1 | Aaron & Hughes (2007) | US | 18-83 | 18-83 | women who identified as lesbian | 416 | interview | sexual,female,31.0% | child sexual abuse |
| 2 | Aberle et al (2007) | Croatia | 14-18 | 14-18 | school pupils | 2140 | self-completed | sexual,male,6.0% sexual,female,3.3% physical,nogender,12.5% emo/psych,nogender,90.3% | Physical and emotional abuse and possible sexual molestation. Calculated for this review as: for physical abuse: frequency of physical punishment = anything over 'never', for emotional abuse: the highest % taken in a list of 'types of punishment' (harassment) |
| 3 | Afifi et al (2003) | Egypt | 12-18 | 12-18 | school pupils | 555 | self-completed | sexual,nogender,7.0% physical,nogender,7.6% emo/psych,nogender,12.3% | sexual, physical, emotional abuse |
| 4 | Afifi et al (2006) | US | 15-54 | 15-54 | population sample | 5877 | self-completed | unspecified,nogender,16.5% | Physical and sexual child abuse, BUT results do not separate types so coded as type of abuse 'unsure' |
| 5 | Afifi et al (2012) | US | 20 and above | 20-100 | population sample | 34653 | interview | physical,nogender,5.9% | physical punishment |
| 6 | Akyuz et al (2005) | Turkey | 18-65 | 18-65 | population sample | 628 | self-completed | sexual,female,2.5% physical,female,24.5% emo/psych,female,21.5% neglect,female,33.9% | sexual, physical, emotional abuse, neglect |
| 7 | Alami and Kadri (2004) | Morocco | 20 and over | 20-100 | Women randomly selected by using systematic sampling from eight jurisdictions in Casablanca | 728 | interview | sexual,female,9.2% | Child sexual abuse |
| 8 | Alikasifoglu et al (2006) | Turkey | mean age 16.3, school pupils 9th to 11th grade | 15-17 | school pupils | 1871 | self-completed | sexual,female,13.4% | sexual abuse in female adolescents |
| 9 | Allard (2009) | Japan and US | 18-28 | 18-28 | university/college students | 79 | self-completed | sexual,nogender,20.5% physical,nogender,9.5% emo/psych,nogender,41.9% neglect,nogender,11.1% | sexual, physical, emotional abuse and neglect |
| 10 | Almeida et al (2011) | Australia | 60-101 | 60-101 | patients who had been in contact with GP in last 12 months | 20677 | self-completed | sexual,nogender,9.5% physical,nogender,11.1% | Early sexual and physical abuse |
| 11 | Ammerman et al (2012) | US | 16 and over | 16-100 | mothers participating in home visitation programs | 90 | self-completed | sexual,female,51.1% physical,female,57.8% emo/psych,female,80.0% neglect,female,73.3% | sexual, physical, emotional, emotional and physical neglect. Highest % taken for emotional and physical neglect |
| 12 | Amodeo et al (2006) | US | 21-65 | 21-65 | population sample | 290 | self-completed and interview | sexual,female,27.9% | child sexual abuse |
| 13 | Angst et al (2011) | Switzerland | 27-30 | 27-30 | population sample | 591 | self-completed | sexual,male,4.8% sexual,female,18.7% | childhood sexual trauma |
| 14 | Annerbäck et al (2010) | Sweden | pupils in grades 7 (ca. 13 years old) and 9 (ca. 15 years old) in compulsory school and grade 2 (ca. 17 years old) in upper secondary school | 13-18 | School pupils | 8494 | self-completed | physical,nogender,15.2% | child physical abuse |
| 15 | Ansara et al (2005) | Canada | 19-42 | 19-42 | women post-childbirth | 253 | interview | sexual,female,14.1% physical,female,6.5% emo/psych,female,3.5% | childhood sexual, physical, emotional abuse |
| 16 | Appel et al (2011) | Germany | 29-89 | 29-89 | population sample | 2157 | self-completed | sexual,nogender,3.6% physical,nogender,4.5% emo/psych,nogender,3.6% neglect,nogender,16.2% | sexual, physical, emotional, emotional and physical neglect. Highest % taken for emotional and physical neglect |
| 17 | Arata et al (2005) | US | 17-61 | 17-61 | university/college students | 384 | self-completed | sexual,nogender,4.4% physical,nogender,4.4% emo/psych,nogender,2.3% neglect,nogender,15.4% | sexual, physical, emotional abuse and neglect. |
| 18 | Ariga et al (2008) | Japan | 16-19 | 16-19 | female juvenile offenders | 64 | interview | sexual,female,54.7% unspecified,female,32.8% | sexual, physical, psychological abuse, does not split physical and psychological abuse so listed as unsure |
| 19 | Arreola et al (2008) | US | 18 and over | 18-100 | gay and bisexual men | 2506 | interview | sexual,male,21.0% | forced sex before the age of 18 |
| 20 | Arreola et al (2009) | US | mean age 31.2 | 31.2 | Latino gay and bisexual men | 912 | interview | sexual,male,15.8% | childhood sexual abuse |
| 21 | Arreola et al (2005) | US | over 18 | 18-100 | Adult men who have sex with men | 2692 | interview | sexual,male,13.0% | childhood sexual abuse |
| 22 | Aslund et al (2007) | Sweden | 15-18 | 15-18 | school pupils | 5048 | self-completed | sexual,male,11.9% sexual,female,29.0% | sexual abuse |
| 23 | Aspelmeier et al (2007) | US | 18-21 | 18-21 | university/college students | 324 | self-completed | sexual,female,37.7% | childhood sexual abuse |
| 24 | Audu et al (2009) | Nigeria | under 18 | 7-18 | Girls engaged in economic activity | 316 | interview | sexual,female,77.7% | child sexual assault |
| 25 | Baccini et al (2003) | Italy | 17-72 | 17-72 | gastrointestinal out-patients | 260 | self-completed | sexual,nogender,17.0% physical,nogender,25.0% | childhood sexual and physical abuse |
| 26 | Back et al (2003) US | US | 17-47 | 17-24 | university/college students | 65 | self-completed | sexual,female,15.4% physical,nogender,38.5% | child sexual and physical abuse |
| 27 | Back et al (2003) Singapore | Singapore | 18-21 | 18-21 | university/college students | 88 | self-completed | sexual,female,4.5% physical,nogender,62.5% | child sexual and physical abuse |
| 28 | Bailey et al (2012) | Australia | 20-73 | 20-73 | adults with current depression and alcohol use problems | 221 | self-completed | sexual,male,21.3% sexual,female,46.8% | sexual assault. Various questions used to measure, highest % taken |
| 29 | Balsam et al (2010) | US | 18-74 | 18-74 | Lesbian, gay and bisexual adults | 669 | self-completed | sexual,male,32.0% sexual,female,42.4% physical,male,35.3% physical,female,37.5% emo/psych,male,50.2% emo/psych,female,60.8% | sexual, physical, emotional abuse |
| 30 | Bandelow et al (2005) | Germany | mean ages 30.2 for patients and 32.3 for controls | 31.3 | Patients with borderline personality disorder and healthy controls | 175 | self-completed | sexual,nogender,30.9% physical,nogender,50.9% emo/psych,nogender,11.4% | sexual abuse, father/mother beats child, father beats mother. Numerous questions asked and so highest % taken. |
| 31 | Banerjee et al (2008) | India | 8-14 | 8-14 | Children employed as whole time workers | 330 | interview | sexual,nogender,3.4% physical,nogender,18.8% emo/psych,nogender,19.9% | sexual abuse, beating, and rebukes or mental assaults |
| 32 | Banou et al (2009) | US | 26-77 | 26-77 | cancer patients | 64 | interview | unspecified,female,59.4% | sexual and physical abuse, and witness to violence. BUT results do not separate types so coded as type of abuse 'unsure' |
| 33 | Barney (2004) | US | 12-19 | 12-19 | gay and heterosexual American Indian or Alaska Native males | 5602 | self-completed | sexual,male,2.5% physical,male,7.6% | sexual and physical abuse |
| 34 | Bebbington et al (2011) | England | 16 and over | 16-100 | population sample | 7353 | self-completed | sexual,male,5.3% sexual,female,11.1% | child sexual abuse |
| 35 | Bensley et al (2000) | US | 18 and older | 18-100 | population sample | 3473 | interview | sexual,male,11.4% sexual,female,13.8% physical,male,9.4% physical,female,4.4% | sexual and physical abuse |
| 36 | Bensley et al (2003) | US | 18 and over | 18-100 | population sample | 3527 | interview | sexual,female,9.6% physical,female,2.0% emo/psych,female,6.1% | physically or sexually assaulted or witnessed interparental violence in childhood |
| 37 | Berliner et al (2001) | US | 18 - 96 | 18-96 | population sample | 1325 | interview | sexual,female,18.0% | sexual assault experiences - child rape or molestation. Highest % was taken as measure |
| 38 | Bifulco et al (2000) | UK | 20-45 | 20-45 | sister pairs | 198 | interview | unspecified,female,40.4% | sexual and physical abuse and neglect. BUT results do not separate types so coded as type of abuse 'unsure' |
| 39 | Birdthistle et al (2008) | Zimbabwe | 15-19 | 15-19 | population sample | 863 | interview | sexual,female,52.2% | participant answered yes to 'ever forced to have sex' |
| 40 | Blain et al (2012) | US | 19-63 | 19-63 | gay and bisexual men reporting compulsive sexual behaviour symptoms | 182 | self-completed | sexual,male,39.0% | child sexual abuse |
| 41 | Bohn et al (2013) | Germany | 23-69 | 23-69 | patients diagnosed with fibromyalgia syndrome | 117 | self-completed | sexual,nogender,28.2% physical,nogender,24.0% emo/psych,nogender,47.9% neglect,nogender,53.8% | sexual, physical, emotional, emotional and physical neglect. Highest % taken for emotional and physical neglect |
| 42 | Bonomi et al (2008) | US | 18-64 | 18-64 | insured women | 3568 | interview | sexual,female,19.4% physical,female,6.4% | sexual and physical abuse |
| 43 | Boynton-Jarrett et al (2012) | US | 21-69 | 21-69 | self-identified black women | 33298 | self-completed | unspecified,female,26.9% | sexual and physical abuse. BUT results do not separate types so coded as type of abuse 'unsure'. Highest % taken for various types and severity of abuse |
| 44 | Bradley et al(2008) | US | 18-81 | 18-81 | patients with and without major depressive disorder | 200 | interview | unspecified,nogender,34.0% | moderate to severe child abuse |
| 45 | Brand et al (2010) | US | mean age 34 | 34 | postpartum women with history of major depressive disorder | 126 | self-completed | sexual,female,22.2% physical,female,7.1% | sexual and physical abuse |
| 46 | Brennan et al (2007) | US | at least 18 years of age | 18-100 | gay and bisexual men | 862 | self-completed | sexual,male,15.5% | childhood sexual abuse |
| 47 | Brezo et al (2008) | Canada | 19-24 | 19-24 | school pupils | 1684 | interview | sexual,male,2.7% sexual,female,18.0% physical,male,26.3% physical,female,14.3% | contacts sexual abuse and physical abuse |
| 48 | Briere and Elliott (2003) | US | 18-90 | 18-90 | population sample | 935 | self-completed | sexual,male,14.2% sexual,female,32.3% physical,male,22.2% physical,female,19.5% | childhood physical and sexual abuse |
| 49 | Brodsky et al (2001) | US | mean age 35.4 for abused and 37.2 for non-abused | 36.3 | depressed adult inpatients | 136 | self-completed | unspecified,nogender,38.0% | sexual and physical abuse. BUT results do not separate types so coded as type of abuse 'unsure'. |
| 50 | Brooker et al (2001) | UK | 18-24 | 18-24 | random probability sampling throughout UK | 2869 | interview | sexual,male,2.5% sexual,female,4.0% physical,male,27.0% physical,female,23.0% emo/psych,male,17.0% emo/psych,female,16.0% neglect,male,19.0% neglect,female,16.0% | Physical maltreatment, physical neglect, emotional and psychological maltreatment and sexual abuse |
| 51 | Brown et al (2005) | US | at least 18, young adults | 18-100 | population sample | 642 | self-completed | sexual,nogender,48.2% physical,nogender,11.6% | sexual and physical abuse |
| 52 | Brown et al (2013) | UK | 19-51 | 19-51 | systematically enriched for risk factors for depressive disorders | 273 | interview | physical,female,11.0% emo/psych,female,35.5% | mother’s lack of affection or rejection, and father’s physical abuse |
| 53 | Cawson et al (2000) | UK | 18-24 | 18-24 | population sample | 2869 | self-completed | sexual,nogender,11.0% physical,nogender,14.0% emo/psych,nogender,6.0% neglect,nogender,6.0% | sexual, physical, emotional/psychological, neglect |
| 54 | Champion et al (2004) | Mexico | 14-19 | 14-19 | women attending a rural health clinic | 106 | self-completed | sexual,female,24.0% physical,female,29.0% emo/psych,female,63.0% | Psychological abuse, physical abuse, sexual abuse |
| 55 | Chapman et al (2004) | US | mean age 56.6 | 56.6 | health maintenance organisation members in a primary care clinic | 9460 | self-completed | sexual,male,6.9% sexual,female,13.2% physical,male,14.7% physical,female,15.4% emo/psych,male,5.1% emo/psych,female,7.5% | sexual, physical, emotional abuse, battered mother. Took the highest % out of emotional abuse and battered mother |
| 56 | Chartier et al (2007) | US | 15-98 | 15-98 | population sample | 9953 | interview | sexual,male,4.3% sexual,female,12.8% physical,male,31.2% physical,female,21.1% | sexual and physical abuse |
| 57 | Chartier et al (2009) | Canada | 15-64 | 15-64 | population sample | 8116 | self-completed | sexual,nogender,9.0% physical,nogender,26.0% | sexual and physical abuse |
| 58 | Chen et al (2004) | China | 11-12 | 16-18 | school pupils | 2300 | self-completed | sexual,male,10.5% sexual,female,16.7% | child sexual abuse |
| 59 | Chen et al (2006) | China | 16-23 | 16-23 | school pupils | 351 | self-completed | sexual,female,21.9% | child sexual abuse |
| 60 | Cheng-Fang et al (2008) | Taiwan | Junior high school pupils | 12-15 | school pupils | 1684 | self-completed | sexual,male,3.0% sexual,female,2.0% physical,male,21.9% physical,female,22.5% | child physical and sexual abuse |
| 61 | Clemmons et al (2003) | US | 18-49 | 18-49 | Latina university students | 112 | self-completed | sexual,female,38.4% physical,female,10.7% emo/psych,female,33.9% | sexual, physical emotional abuse and witness violence between parents. Took the highest % for emotional abuse or witnessing violence |
| 62 | Cohen et al (2001) | US | mean age 22 | 22 | population sample | 664 | self-completed | sexual,nogender,2.9% physical,nogender,3.5% | sexual and physical abuse |
| 63 | Cohen et al (2006) US | US | 18-70 | 18-70 | healthy volunteers | 1659 | self-completed | sexual,nogender,5.3% physical,nogender,4.7% emo/psych,nogender,14.8% | sexual, physical, emotional abuse, domestic violence in the home. Highest % taken from domestic violence and emotional abuse |
| 64 | Cohen et al (2006) UK & Amsterdam | UK & Amsterdam | 18-70 | 18-70 | healthy volunteers | 1659 | self-completed | sexual,nogender,7.7% physical,nogender,7.7% emo/psych,nogender,16.5% | sexual, physical, emotional abuse, domestic violence in the home. Highest % taken from domestic violence and emotional abuse |
| 65 | Cohen et al (2006) Australia | Australia | 18-70 | 18-70 | healthy volunteers | 1659 | self-completed | sexual,nogender,4.2% physical,nogender,5.0% emo/psych,nogender,11.8% | sexual, physical, emotional abuse, domestic violence in the home. Highest % taken from domestic violence and emotional abuse |
| 66 | Coid et al (2003) | UK | 16-85 | 16-85 | patients in a primary care clinic | 1207 | self-completed | sexual,female,11.0% physical,female,12.0% | childhood sexual and physical abuse |
| 67 | Collishaw et al (2007) | UK | 42-46 | 42-46 | adults recruited when they were school pupils | 378 | interview | sexual,nogender,7.8% physical,nogender,4.7% | repeated or very severe sexual abuse, physical abuse |
| 68 | Collin-Vezina et al (2005) | Canada | 23-51 | 23-51 | mothers referred to or delegated responsibility to Youth Protection Services | 93 | self-completed | sexual,female,56.8% | child sexual abuse |
| 69 | Comijs et al (2013) | Netherlands | 60-93 | 60-93 | depressed and non-depressed persons | 510 | interview | sexual,nogender,18.0% physical,nogender,12.2% emo/psych,nogender,21.0% neglect,nogender,32.4% | sexual, physical, psychological abuse and emotional neglect |
| 70 | Cong et al (2012) | China | 30-60 | 30-60 | women with and without recurrent major depression | 4508 | interview | sexual,female,5.7% | child sexual abuse |
| 71 | Conroy et al (2009) | Australia | mean 36.5 and 34.7 | 35.6 | participants with and without a history of opioid pharmacotherapy | 1313 | interview | sexual,male,35.7% sexual,female,66.5% physical,male,53.1% physical,female,53.4% emo/psych,male,60.9% emo/psych,female,55.9% neglect,male,65.0% neglect,female,75.5% | sexual, physical, emotional abuse and neglect. % reported participants with and without a history of opioid pharmacotherapy, calculated the numbers these should be, added together and divided by total to give % |
| 72 | Corliss et al (2002) | US | 25-74 | 25-74 | population sample | 3032 | self-completed | physical,male,37.4% physical,female,31.1% emo/psych,male,37.0% emo/psych,female,37.4% | Emotional maltreatment, any physical maltreatment |
| 73 | Cyr et al (2013) | Canada | 12-17 | 12-17 | population sample | 2801 | interview | sexual,nogender,47.7% physical,nogender,41.6% emo/psych,nogender,35.9% neglect,nogender,30.1% | any sexual victimisation or any sexual assault (highest % taken). Physical, psychological/emotional abuse and neglect. For all these, n for two age groups calculated and then added together and divided by 2. |
| 74 | Dalenberg & Palesh (2004) | Russia | 15-55 | 15-55 | university/college students | 301 | self-completed | sexual,nogender,5.7% physical,nogender,11.9% | Child physical trauma, child sexual abuse |
| 75 | Danese et al (2008) | New Zealand | 26 years | 26 | population sample | 1000 | self-completed | sexual,nogender,12.0% physical,nogender,4.0% | sexual and physical abuse |
| 76 | De Von Figueroa-Moseley (2004) | US | 18-74 | 18-74 | university/college students | 296 | self-completed | sexual,female,29.4% | sexual abuse in childhood |
| 77 | Decker et al (2007) | US | 14-17 | 14-17 | school pupils | 5919 | self-completed | sexual,female,15.0% | Sexual Violence Against Adolescent Girls |
| 78 | Decker et al (2010) | Thailand | 14-17 | 14-17 | female sex workers | 815 | interview | unspecified,female,25.0% | physical or sexual violence victimisation, does not split physical and sexual abuse so listed as unsure |
| 79 | Dennis et al (2009) | US | mean age 40 | 40 | women with and without PTDS and major depression | 148 | self-completed | sexual,female,45.3% physical,female,45.9% | childhood sexual trauma, childhood violence |
| 80 | Deyessa et al (2009) | Ethiopia | 15-49 | 15-49 | married women living in the Butajira Rural Health Programme area | 1943 | self-completed | sexual,female,8.5% | childhood sexual abuse |
| 81 | Diaz et al (2002) | US | 10-18 | 10-18 | school pupils | 3575 | self-completed | sexual,female,5.0% physical,female,8.0% | physical and sexual abuse |
| 82 | Diaz-Olavarrieta et al (2001) | Mexico | 17-85 | 17-85 | Nurses and nurses aids | 1150 | self-completed | sexual,female,5.0% physical,female,10.7% | sexual and physical abuse during childhood |
| 83 | Dibble et al (2007) | US | 40-77 | 40-77 | women who identified as lesbian and were Asian, Native Hawaiian, or other Pacific Islander | 29 | self-completed | sexual,female,34.7% physical,female,28.6% | sexual and physical abuse |
| 84 | Dietz (2009) | US | 50 years and older | 50-100 | homeless adults | 862 | interview | sexual,nogender,4.0% physical,nogender,10.0% nogender,6.0% | sexual and physical abuse and neglect |
| 85 | Dolezal & Carballo-Dieguez (2002) | US | 18-54 | 18-54 | Latino men who have sex with men | 307 | interview | sexual,male,59.0% | participant considers a childhood sexual experience as sexual abuse |
| 86 | Dong et al (2004) | US | mean age 55 for women and 57 for men | 18-100 | adult members of a health plan | 8629 | self-completed | sexual,nogender,21.0% physical,nogender,26.4% emo/psych,nogender,24.1% neglect,nogender,14.8% | sexual abuse, physical abuse, emotional abuse, physical and emotional neglect, witnesses domestic violence. Highest % taken for physical and emotional neglect, and emotional abuse and witnessed domestic violence. |
| 87 | Draper et al (2008) | Australia | 60 and older | 60-100 | older patients recruited though their GP | 22251 | self-completed | sexual,nogender,6.5% physical,nogender,6.7% | sexual and physical abuse |
| 88 | Dube et al (2001) | US | mean age 56 | 56 | health maintenance organisation members | 17337 | self-completed | sexual,male,16.0% sexual,female,24.7% physical,male,27.0% physical,female,29.9% emo/psych,male,13.7% emo/psych,female,11.5% | sexual, physical, emotional abuse and battered mother. Highest % taken for emotional abuse of battered mother |
| 89 | Dube et al (2003) | US | mean age 55 and 57 | 56 | health maintenance organisation members | 8613 | self-completed | sexual,male,17.1% sexual,female,24.3% physical,male,27.9% physical,female,25.1% emo/psych,male,7.8% emo/psych,female,12.2% neglect,male,12.4% neglect,female,16.7% | sexual, physical, emotional abuse and emotional and physical neglect. Highest % taken for emotional and physical neglect |
| 90 | Duke et al (2010) | US | 10-19 | 10-19 | school pupils | 136549 | self-completed | sexual,male,2.7% sexual,female,7.4% physical,male,9.1% physical,female,11.6% emo/psych,male,9.9% emo/psych,female,13.3% | sexual and physical abuse and witnessing physical abuse by family member on another family member. Two sexual abuse questions, highest number taken |
| 91 | Duncan (2000) | US | 17-20 | 17-20 | university/college students | 210 | self-completed | sexual,nogender,20.0% physical,nogender,11.0% emo/psych,nogender,18.0% | sexual, physical, psychological abuse |
| 92 | Dunkle et al (2004) | South Africa | 16-44 | 16-44 | women attending antenatal clinics | 1395 | self-completed | sexual,female,8.0% | child sexual assault |
| 93 | Dunne et al (2003) | Australia | 18-59 | 18-59 | population sample | 1784 | interview | sexual,male,15.9% sexual,female,33.6% | sexual abuse in childhood. Took highest % in list of experiences. |
| 94 | Duran et al (2004) | US | 18-45 | 18-45 | women attended outpatient ambulatory services at a hospital | 234 | self-completed | sexual,female,23.1% physical,female,17.5% emo/psych,female,26.1% neglect,female,22.6% | sexual, physical, emotional abuse, neglect. Took highest % out of 3 categories of maltreatment severity. |
| 95 | Dussich & Maekoya (2007) | Japan, South Africa, US | mean ages 18.5 for Japan, 19.3 For South Africa, 20.6 for US | 19.5 | university/college students | 852 | self-completed | physical, male, Japan 56.6%  phys, male, S Africa 72.3%  phys, male, US 63.5%  phys, female, Japan 58.0%  phys, female, S Africa 65.5%  phys, female, US 54.7% | physical child abuse |
| 96 | Edgardh and Ormstad (2000) | Sweden | 17 | 17 | School pupils and non-attenders | 2153 | self-completed | sexual,male,4.0% sexual,female,28.0% | sexual abuse |
| 97 | Edwards et al (2003) | US | 19-97 | 19-97 | adults in a health maintenance organization (HMO) | 8667 | self-completed | sexual,male,17.5% sexual,female,25.0% physical,male,21.0% physical,female,19.7% emo/psych,male,12.3% emo/psych,female,15.9% | Sexual abuse, physical abuse, witness to maternal battery, |
| 98 | Eisenberg et al (2007) | US | 11-12, 14-15, 17-18 | 11-18 | school pupils | 124881 | self-completed | sexual,male,4.0% sexual,female,9.7% | sexual abuse |
| 99 | Enns et al (2006) | Netherlands | 18-64 | 18-64 | population sample | 7076 | interview | sexual,nogender,6.9% physical,nogender,8.9% emo/psych,nogender,12.9% neglect,nogender,24.8% | sexual, physical, psychological abuse and neglect. % taken for measures taken at baseline |
| 100 | Eskin et al (2005) | Turkey | 17-43 | 17-43 | university/college students | 1262 | self-completed | sexual,nogender,28.1% | childhood sexual abuse |
| 101 | Evans-Campbell et al (2006) | US | 18-77 | 18-77 | population sample of American Indian/Alaska Natives | 112 | interview | physical,female,28.2% | physical abuse |
| 102 | Evren & Evren (2005) | Turkey | mean age 28.5 for patients with substance-dependence and 40.6 for patients without substance dependence | 34.6 | males with substance dependence with and without self-mutilation | 136 | interview | sexual,male,7.3% physical,male,31.6% emo/psych,male,23.6% neglect,male,43.4% | sexual, physical, emotional abuse and neglect. Numbers reported for with and without suicide ideation, added these together |
| 103 | Evren et al (2006) | Turkey | over 18 | 18-100 | adults with substance dependence | 132 | self-completed | sexual,nogender,12.1% physical,nogender,40.2% emo/psych,nogender,32.6% neglect,nogender,36.4% | sexual, physical, emotional abuse, physical or emotional neglect |
| 104 | Everson et al (2008) | US | 12-13 | 12-13 | At-risk children involved in LONGSCAN (longitudinal study of child maltreatment [Runyan et al, 1998]) | 350 | self-completed | sexual,nogender,9.0% physical,nogender,21.0% emo/psych,nogender,39.0% | Physical, Sexual, and Psychological childhood Abuse |
| 105 | Fakhari et al (2012) | Iran | mean age 14.9 | 14.9 | school pupils | 399 | self-completed | sexual,female,10.7% physical,female,22.5% emo/psych,female,24.5% neglect,female,25.4% | sexual and physical abuse, abusive language, humiliation, discrimination, unkindness, neglect. Abusive language, humiliation, discrimination, unkindness taken as emotional/psychological abuse, highest % taken. Sexual abuse reported as inside and outside the home, highest % taken. Various frequencies provided, highest % taken when over 'seldom' (no 'never' option) |
| 106 | Fanslow et al (2007) | New Zealand | 18-64 | 18-64 | population sample | 2855 | interview | sexual,female,28.2% | child sexual abuse |
| 107 | Feldman & Meyer (2007) | US | mean age 33 | 33 | gay and biexual men | 193 | interview | sexual,male,34.0% physical,male,33.0% | sexual and physical abuse |
| 108 | Feng et al (2015) | Taiwan | 12-18 | 12-18 | school pupils | 5236 | self-completed | sexual,male,21.8% sexual,female,17.7% physical,male,61.9% physical,female,61.1% emo/psych,male,69.3% emo/psych,female,69.3% neglect,male,50.2% neglect,female,59.4% | sexual, physical, emotional abuse and neglect. |
| 109 | Fergusson et al (2000) | New Zealand | 18 and 21 | 18-21 | Participants form a birth cohort study | 980 | interview | sexual,male,6.1% sexual,female,30.4% | child sexual abuse |
| 110 | Fergusson et al (2008) | New Zealand | 18-25 | 18-25 | young adults in birth cohort | 1265 | self-completed | sexual,nogender,6.4% | child sexual abuse. Contact and non-contact CSA, highest % taken |
| 111 | Figueiredo et al (2004) | Portugal | 22-84 | 22-84 | Parents and grandparents of school pupils | 932 | self-completed | sexual,male,2.6% sexual,female,2.7% physical,male,74.3% physical,female,70.1% | physical abuse and sexual abuse |
| 112 | Fillingim & Edwards (2005) | US | mean age 22.1 for females and 23 for males | 22.6 | university/college students | 110 | self-completed | sexual,male,7.3% sexual,female,19.1% physical,male,4.5% physical,female,0.9% | sexual and physical childhood abuse. |
| 114 | Finkelhor et al (2014) | US | 15-17 | 15-17 | population sample | 2293 | interview | sexual,male,5.1% sexual,female,26.6% | child sexual abuse |
| 114 | Finkelhor et al (2015) | US | 14-17 | 14-17 | population sample | 4000 | interview | physical,nogender,18.1% emo/psych,nogender,23.9% neglect,nogender,18.4% | physical and emotional abuse by a caregiver, and neglect. |
| 115 | Fisher et al (2011) | UK | 16-64 | 16-64 | individuals presenting to mental health services with psychosis | 157 | self-completed | sexual,nogender,19.2% physical,nogender,25.5% emo/psych,nogender,23.6% neglect,nogender,19.7% | sexual and physical abuse, paternal antipathy (taken as emotional abuse), and paternal neglect |
| 116 | Fisher et al (2013) | UK | 20-82 | 20-82 | individuals with and without recurrent unipolar depression | 455 | self-completed | sexual,nogender,12.7% physical,nogender,8.4% emo/psych,nogender,21.1% neglect,nogender,25.1% | sexual, physical, emotional abuse, physical or emotional neglect. Moderate and severe maltreatment measured so highest % taken, and highest % for physical and emotional neglect |
| 117 | Flynn O'Brien et al (2016) | Haiti | 13-24 | 13-24 | population sample | 2916 | interview | physical,male,66.6% physical,female,67.4% | childhood physical violence |
| 118 | Fogarty et al (2008) | US | 18 and over | 18-100 | population sample | 7918 | self-completed | unspecified,female,16.0% | sexual and physical abuse. BUT results do not separate types so coded as type of abuse 'unsure'. |
| 119 | Fricker et al (2003) | US | 18-47 | 18-47 | university/college students | 236 | self-completed | sexual,male,39.0% sexual,female,29.0% | Sexual abuse. |
| 120 | Friedman et al (2002) | US | mean age 39 | 39 | Patients recruited through a general psychiatry outpatients clinic | 201 | interview | sexual,nogender,27.5% physical,nogender,31.0% | sexual and physical abuse |
| 121 | Friedman et al (2008) | US | 18-40 | 18-40 | gay and bisexual men | 1383 | interview | physical,male,27.8% | Parental physical abuse. Various gay-related development stages reported, highest % taken. |
| 122 | Fuemmeler et al (2009) | US | mean age 22 | 22 | population sample | 15197 | self-completed | sexual,male,4.8% sexual,female,4.1% physical,male,13.6% physical,female,15.6% male,4.8% female,5.3% | sexual and physical abuse and neglect. |
| 123 | Fujiwara et al (2010) (b) | Japan | 19-56 | 19-56 | mothers who are experiencing family problems and are in mother-child home facilities | 421 | self-completed | unspecified,female,45.6% | childhood abuse, does not specify type. |
| 124 | Fujiwara et al (2011) | Japan | 20 and above | 20-100 | population sample | 1722 | self-completed | sexual,nogender,0.5% physical,nogender,7.5% neglect,nogender,1.5% | sexual and physical abuse and neglect |
| 125 | Fuller-Thompson et al (2010) | Canada | 12 and over | 12-100 | population sample | 13089 | self-completed | physical,nogender,7.4% | physical abuse |
| 126 | Gagne et al (2005) | Canada | 14-20 | 14-20 | school pupils | 622 | self-completed | sexual,female,29.0% | sexual abuse. Took highest % out of 2 categories |
| 127 | Gallagher et al (2002) | UK | 9-16 | 9-16 | school pupils | 2420 | self-completed | sexual,nogender,22.0% | child sexual abuse |
| 128 | Gamble et al (2007) | US | 50-84 | 50-84 | patients with depression | 187 | self-completed | sexual,nogender,46.6% | childhood sexual abuse |
| 129 | Garcia et al (2002) | US | 18-30 | 18-30 | University students, gay/bisexual/lesbian and heterosexual | 138 | self-completed | sexual,male,16.4% sexual,female,45.8% physical,male,18.2% physical,female,32.5% emo/psych,male,45.5% emo/psych,female,63.9% | sexual, physical, emotional abuse. Highest % taken from a range of questions on each of these. Added together n for lesbian/bisexual/gay and homosexual to give total |
| 130 | Garcia-Moreno et al (2005) | Bangladesh, Brazil, Ethiopia, Japan, Namibia, Peru, Samoa, Serbia and Montenegro, Thailand, United Republic of Tanzania | 15-49 | 15-49 | population sample | 24097 | self-completed | sexual, female. Bangladesh 7.0%, Peru 19.0%, Brazil 12.0%, Ethiopia 7.0%, Japan 14.0%, Namibia 21.0%, Samoa 2.0%, Serbia and Montinegro 4.0%, Thailand 9.0%, Tanzania 11.0% | asked directly whether anyone had ever touched them sexually, or made them do something sexual that they did not want to before the age of 15 years. |
| 131 | Gaudiano & Zimmerman (2010) | US | 18-79 | 18-79 | adult outpatients diagnosed with Major Depressive Disorder | 623 | interview | sexual,nogender,36.0% physical,nogender,38.5% emo/psych,nogender,40.0% neglect,nogender,38.4% | sexual, physical, emotional abuse, physical neglect |
| 132 | Gault-Sherman et al (2009) | Iceland | 16-29 | 16-20 | school pupils | 8618 | self-completed | sexual,male,6.3% sexual,female,17.6% | sexual abuse. |
| 133 | Gerke et al (2006) | US | 16-53 | 16-53 | university/college students | 417 | self-completed | sexual,female,19.0% physical,female,53.0% emo/psych,female,78.0% neglect,female,76.0% | sexual, physical, emotional abuse, physical or emotional neglect. Highest % taken for physical or emotional neglect |
| 134 | Gibb et al (2003) | US | mean 40.59 | 40.6 | psychiatric outpatients | 552 | interview | sexual,nogender,6.9% physical,nogender,11.2% emo/psych,nogender,34.8% | sexual, physical, emotional abuse |
| 135 | Gladstone et al (2004) | Australia | 17-68 | 17-68 | women with depressive disorders | 126 | self-completed | sexual,female,29.4% | childhood sexual abuse |
| 136 | Goodwin & Stein (2004) | US | 15-54 | 15-54 | population sample | 5877 | self-completed | sexual,male,3.5% sexual,female,17.2% physical,male,11.8% physical,female,9.6% neglect,male,2.9% neglect,female,2.5% | sexual and physical abuse and neglect |
| 137 | Goodwin et al (2003) | US | 25-74 | 25-74 | population sample | 3032 | self-completed | unspecified,nogender,15.8% | childhood abuse, does not specify type. |
| 138 | Goodwin et al (2005) | New Zealand | 18-21 | 18-21 | birth cohort sample | 983 | self-completed | sexual,nogender,11.4% physical,nogender,6.0% | childhood sexual and physical abuse |
| 139 | Gratz et al (2002) | US | 18-49 | 18-49 | university/college students | 133 | self-completed | sexual,male,14.0% sexual,female,30.0% physical,male,21.0% physical,female,29.0% neglect,male,7.0% neglect,female,3.0% | sexual and physical abuse and physical neglect |
| 140 | Grayson et al (2005) | US | 25-75 | 25-75 | population sample | 1327 | interview | sexual,nogender,24.0% | childhood sexual abuse |
| 141 | Green et al (2010) | US | 18 and over | 18-100 | population sample | 5692 | interview | sexual,nogender,6.0% physical,nogender,8.4% nogender,5.6% | sexual and physical abuse and neglect |
| 142 | Groleau et al (2012) | Canada | mean age 24.95 for bulemic women and 23.91 for normal eaters | 25.4 | women with and without a bulimic eating disorder | 315 | interview | sexual,female,17.1% physical,female,33.3% emo/psych,female,68.3% | sexual, physical and emotional abuse |
| 143 | Grote et al (2012) | US | 18 years or older | 18-100 | pregnant, depressed, nontreatment seeking women on low incomes | 53 | self-completed | sexual,female,17.3% physical,female,38.5% emo/psych,female,40.5% neglect,female,40.5% | sexual, physical, emotional abuse, physical or emotional neglect. Highest % taken for physical or emotional neglect |
| 144 | Gunn et al (2008) | Australia | 18-76 | 18-76 | Adult patients with depressive symptoms | 789 | self-completed | sexual,nogender,29.5% physical,nogender,48.2% | sexual and physical abuse |
| 145 | Gunnlaugsson et al (2011) | Iceland | 14-15 | 14-15 | school pupils | 3515 | self-completed | emo/psych,nogender,51.2% | Witnessed adult physical violence in the home |
| 146 | Gwadz et al (2007) | US | 16-23 | 16-23 | youth who are homeless or at risk for homelessness | 85 | interview | sexual,male,15.0% sexual,female,44.0% physical,male,29.0% physical,female,43.0% emo/psych,male,15.0% emo/psych,female,35.0% neglect,male,51.0% neglect,female,45.0% | sexual, physical, emotional abuse, emotional and physical neglect. Highest number taken from emotional and physical neglect. |
| 147 | Hamburger et al (2008) | US | pupils in grades 7, 9, and 11/12 | 12-18 | school pupils | 3559 | self-completed | sexual,male,6.1% sexual,female,11.5% physical,male,21.2% physical,female,23.4% emo/psych,male,26.3% emo/psych,female,38.1% | sexual and physical abuse and witnessing domestic violence between parents/guardians. Witnessing domestic violence between parents/guardians taken as emotional/psychological abuse |
| 148 | Hamelin et al (2009) | New Caledonia | 18-54 | 18-54 | population sample | 1099 | interview | sexual,female,11.6% | childhood sexual abuse |
| 149 | Handa et al (2008) | Japan | 29-56 | 29-56 | outpatients at the Department of Psychosomatic Medicine in a hospital with a psychiatric disorder | 518 | self-completed | physical,nogender,13.7% | childhood physical abuse |
| 150 | Hanson et al (2001) | US | 18 and over | 18-100 | population sample | 4008 | interview | sexual,female,7.5% physical,female,1.7% | child rape and child aggravated assault |
| 151 | Harkness & Monroe (2002) | US | 18-70 | 18-70 | Women with major depression | 76 | interview | sexual,female,52.6% physical,female,67.1% emo/psych,female,30.3% neglect,female,100.0% | sexual, physical, psychological abuse and neglect. Non-severe and severe reported, total taken. |
| 152 | Harkness et al (2012) | Canada | 18-60 | 18-60 | outpatients with major depressive disorder | 203 | interview | unspecified,nogender,44.8% | severe child maltreatment |
| 153 | Harrison & Narayan (2003) | US | 14-15 | 14-15 | school pupils | 50168 | self-completed | sexual,male,9.6% sexual,female,13.2% physical,male,9.6% physical,female,13.2% | victim of physical abuse at home, victim of sexual abuse |
| 154 | Hasnain & Kumar (2006) | India | adult women | 18-100 | University/college students | 150 | self-completed | sexual,female,38.0% | child sexual abuse |
| 155 | Hegarty et al (2004) | Australia | 16-50 | 16-50 | Women who presented at GP clinics who had ever been in an intimate relationship as an adult | 1210 | self-completed | unspecified,female,27.2% | Abused as child, does not specify type of abuse. |
| 156 | Heidt et al (2005) | US | 18-77 | 18-77 | people who attended gay, lesbian, bisexual, and transgendered community organisations and events | 342 | self-completed | sexual,nogender,30.7% | child sexual abuse |
| 157 | Helweg-Larsen and Bøving Larsen (2006) | Denmark | 15-16 | 15-16 | school pupils | 5829 | self-completed | sexual,male,6.7% sexual,female,15.8% | unlawful early sexual experiences |
| 158 | Henny et al (2007) | US | 19-63 | 19-63 | HIV-seropositive homeless or unstably housed adults | 644 | interview | sexual,male,32.8% sexual,female,52.4% physical,male,50.5% physical,female,57.0% | sexual and physical abuse |
| 159 | Hester et al (2009) | China and UK | no definitive age range, University students | 18-100 | university/college students | 979 | self-completed | physical, male, China 60.0%  physical, male, UK 43.0%  physical, fem, China 50.0%  physical, female, UK 43.3% | physical punishment. |
| 160 | Hetzel et al (2005) | US | 18-221 | 18-21 | university/college students | 467 | self-completed | sexual,female,8.4% physical,female,15.8% | sexual, physical abuse |
| 161 | Hill et al (2000) | UK | 25-36 | 25-36 | socio-economically representative sample of women from GP surgeries | 862 | self-completed | sexual,female,17.5% | child sexual abuse. Took highest% from a range of questions. |
| 162 | Hillis et al (2000) | US | 19 to over 65 | 19-65 | health maintenance organisation members | 5032 | self-completed | sexual,nogender,24.5% physical,nogender,28.6% emo/psych,nogender,13.9% | sexual, physical, emotional abuse and battered mother. Highest % taken for emotional abuse of battered mother |
| 163 | Hovens et al (2010) | Netherlands | 18-65 | 18-65 | participants with and without anxiety and depression | 1931 | interview | sexual,nogender,11.1% physical,nogender,8.5% emo/psych,nogender,13.8% neglect,nogender,22.1% | sexual, physical, psychological abuse and emotional neglect. Highest number taken for frequency |
| 164 | Hovens et al (2012) | Netherlands | 18-65 | 18-65 | individuals with and without a depressive or anxiety disorder | 1209 | interview | sexual,nogender,13.2% physical,nogender,10.5% emo/psych,nogender,18.2% neglect,nogender,27.3% | sexual, physical, psychological abuse and emotional neglect. Highest number taken for frequency |
| 165 | Howard et al (2005) | US | 9th - 12th grade | 14-18 | school pupils | 13601 | self-completed | sexual,male,5.1% sexual,female,10.2% | forced sex |
| 166 | Huang et al (2011) | US | mean age 21.8 | 21.8 | population sample | 4882 | interview | sexual,male,4.4% sexual,female,4.3% physical,male,15.2% physical,female,13.4% male,21.3% female,20.2% | sexual and physical abuse and neglect |
| 167 | Hughes et al (2000) | US | 10-86 | 20-86 | lesbian and heterosexual women | 829 | self-completed | sexual,female,13.6% | childhood sexual abuse |
| 168 | Hughes et al (2001) | US | 18 and over | 18-100 | Lesbian and heterosexual women | 120 | interview | sexual,female,57.5% | child sexual abuse. Reported both Wyatt measure and self-perception, taken highest % |
| 169 | Hussey et al (2006) | US | pupils in grades 7-12 | 12-18 | school pupils | 15197 | interview | sexual,nogender,4.5% physical,nogender,28.4% nogender,41.5% | Supervision neglect, physical assault, physical neglect, and contact sexual abuse |
| 170 | Jewkes et al (2002) | South Africa | 15-49 | 15-49 | population sample | 11735 | interview | sexual,female,1.3% | rape before the age of 15 |
| 171 | Jewkes et al (2010) | South Africa | 15-26 | 15-26 | population sample | 2782 | interview | sexual,male,12.8% sexual,female,23.9% physical,male,84.9% physical,female,73.8% emo/psych,male,34.3% emo/psych,female,31.8% neglect,male,24.2% neglect,female,22.2% | sexual, physical, emotional abuse, emotional negelct. Various frequency taken so highest reported |
| 172 | Jirapramukpitak et al (2005) | Thailand | 16-25 | 16-25 | population sample | 202 | interview | sexual,male,4.9% sexual,female,6.5% physical,male,15.3% physical,female,9.0% emo/psych,male,34.0% emo/psych,female,32.2% | sexual, physical, emotional abuse and witness of maternal battering. Took highest % of emotional abuse or witness of maternal battering. |
| 173 | Jirapramukpitak et al (2011) | Thailand | 16-25 | 16-25 | population sample | 1052 | self-completed | nogender,16.7% physical,nogender,11.6% | physical abuse and exposure to domestic violence |
| 174 | Johnson et al (2006) | US | 18-66 | 18-66 | incarcerated men | 100 | self-completed | sexual,male,59.0% | sexual abuse |
| 175 | Johnstone et al (2009) | New Zealand | mean age 35.5 | 35.5 | depressed patients | 195 | interview | sexual,nogender,12.3% | childhood sexual abuse. Highest % taken for severity of abuse |
| 176 | Johnstone et al (2013) | New Zealand | mean age 32 | 32 | outpatients with depression | 159 | interview | sexual,nogender,10.1% | sexual abuse. Highest % taken for severity of abuse |
| 177 | Joyce et al (2003) | New Zealand | 18 and over | 18-100 | depressed outpatients | 180 | interview | neglect,nogender,27.2% unspecified,nogender,25.0% | childhood neglect and childhood abuse (abuse therefore recorded as unsure as it does not specify type). Highest % taken for severity of abuse and neglect |
| 178 | Ju & Lee (2010) | Republic of Korea | 9-12 | 9-12 | children who had been removed from their families | 357 | interview | physical,nogender,34.7% emo/psych,nogender,24.4% | physical and emotional abuse |
| 179 | Jumaian (2001) | Jordan | 18-20 | 18-20 | University/college students | 100 | self-completed | sexual,male,27.0% | child sexual abuse |
| 180 | Jun et al (2008) | US | 25-42 | 25-42 | Nurses | 68505 | self-completed | sexual,female,14.9% physical,female,25.7% | sexual and physical abuse. Various numbers of severity reported, highest taken |
| 181 | Karayianni et al (2017) | Cyprus | 15-25 | 15-25 | school pupils and university and college students | 1852 | self-completed | sexual,male,21.0% sexual,female,80.0% | sexual abuse in childhood |
| 182 | Keeshin & Campbell (2011) | US | 18-23 | 18-23 | homeless adults | 64 | interview | sexual,male,3.0% sexual,female,4.0% physical,male,20.0% physical,female,31.0% | sexual and physical abuse. Sexual and physical abuse only and combined reported, % for the sexual and physical abuse only taken |
| 183 | Kendler et al (2000) | US | 17-55 | 17-55 | female adult twins | 1411 | self-completed | sexual,male,21.0% | sexual abuse. Took highest % from a range of questions |
| 184 | Kenny & McEachern (2000) | US | 19-57 | 19-57 | university/college students | 164 | self-completed | sexual,female,18.0% | childhood sexual abuse |
| 185 | Kerr et al (2009) | Canada | 14-26 | 14-26 | high-risk youth | 560 | interview | sexual,nogender,26.8% physical,nogender,40.7% emo/psych,nogender,49.6% neglect,nogender,45.7% | sexual, physical, emotional abuse and emotional and physical neglect. Highest % taken for emotional and physical neglect |
| 186 | Khamis (2000) | Palestine | 12-16 | 12-16 | school pupils | 1000 | interview | sexual,male,0.0% sexual,female,0.9% physical,nogender,14.1% | sexual abuse, physical abuse |
| 187 | Kilpatrick et al (2000) | US | 12-17 | 17-17 | population sample | 4023 | interview | sexual,nogender,8.0% physical,nogender,22.0% | sexual and physical assault |
| 188 | Kim and Kim (2005) | Korea | 12-18 | 12-18 | school pupils | 1672 | self-completed | sexual,male,0.5% sexual,female,10.2% | Sexual abuse and incest. Incest was defined as a clear and conscious memory by the victim of at least one incident of unwanted sexual penetration of a bodily orifice by an older blood relative occurring either by threat or force. |
| 189 | Kim et al (2009) | Republic of Korea | average age 42.39 | 42.4 | population sample | 1079 | interview | physical,female,23.1% emo/psych,female,31.3% | verbal and physical violence by parents |
| 190 | King et al (2004) | South Africa | 12 - 18 and over | 12-18 | school pupils | 939 | self-completed | sexual,nogender,8.4% | attempted and actual rape. Took the highest % |
| 191 | Kitamura et al (2000) | Japan | 18-91 | 18-91 | population sample | 220 | interview | physical,male,14.6% physical,female,9.7% emo/psych,male,20.8% emo/psych,female,14.5% | Scolding, slapping, punching, hitting, burning. Scolding taken as psychological abuse, rest of items taken as physical abuse. Many items, took highest number. |
| 192 | Kong & Bernstein (2009) | Korea | 14-36 | 14-36 | patients with eating disorders | 74 | self-completed | sexual,nogender,30.1% physical,nogender,53.4% emo/psych,nogender,65.8% neglect,nogender,74.0% | sexual, physical, emotional abuse, physical or emotional neglect. Highest % taken for physical or emotional neglect |
| 193 | Kounou et al (2013) | Togo | 18-65 | 18-65 | individuals with and without major depressive disorder | 181 | interview | sexual,nogender,41.9% physical,nogender,25.9% emo/psych,nogender,28.2% neglect,nogender,44.8% | sexual, physical, emotional abuse, physical or emotional neglect. Highest % taken for physical or emotional neglect. n reported for with and without MD separately, added these together. |
| 194 | Kraaij & de Wilde (2001) | Netherlands | 65 and over | 65-100 | population sample | 194 | self-completed | sexual,nogender,3.2% physical,nogender,5.3% emo/psych,nogender,22.3% | sexual and physical abuse and emotional abuse or neglect. Emotional abuse or neglect therefore recorded as emotional/psychological abuse. |
| 195 | Kvam (2004) | Norway | 18-65 | 18-65 | Adult deaf Norwegians | 302 | self-completed | sexual,male,42.4% sexual,female,45.8% | sexual abuse |
| 196 | Leeners et al (2006) | Germany | mean age 31.6 for those who experienced CSA and 32 for those who did not | 31.8 | Women who had recently given birth | 226 | self-completed | sexual,female,11.5% | child sexual abuse |
| 197 | Lehavot et al (2009) | US | 18 years or older | 18-100 | Lesbian, bisexual, and two-spirit women | 152 | self-completed | sexual,female,76.0% | child sexual contact |
| 198 | Lepisto et al (2011) | Finland | 14-17 | 14-17 | school pupils | 1393 | self-completed | physical,nogender,43.1% emo/psych,nogender,42.3% | Witnessing domestic violence, parental expressions of symbolic aggression, parental mild violence, parental severe violence. Witnessing domestic violence and parental expressions of symbolic aggression taken to be emotional/psychological abuse, highest %taken. Parental mild and severe violence taken to be physical abuse, highest % taken. |
| 199 | Lewis et al (2003) | US | mean 21.53 | 21.5 | university/college students | 255 | self-completed | sexual,female,64.0% | sexual abuse |
| 200 | Li et al (2012) | Taiwan | 15-24 | 15-24 | school pupils and university and college students | 4084 | self-completed | sexual,male,4.3% sexual,female,6.2% | child sexual abuse |
| 201 | Libby et al (2005) | US | 15-54 | 15-54 | Participants from American Indian Tribes | 3084 | self-completed | sexual,male,1.6% sexual,female,7.3% physical,male,6.7% physical,female,8.1% | sexual and physical abuse. n provided for two tribes, added and divided by 2 |
| 202 | Logan et al (2009) | US | 7th grade | 12-13 | high-risk youth | 1484 | self-completed | physical,nogender,18.9% | early physical abuse |
| 203 | Lu et al (2008) | US | mean age 42.87 | 42.9 | adults with major mood disorders | 254 | interview | sexual,male,28.0% sexual,female,58.0% physical,male,58.0% physical,female,54.0% | sexual and physical abuse |
| 204 | Lutenbacher et al (2000) | US | 16-41 | 16-41 | low-income single mothers with a young child | 59 | interview | sexual,female,15.0% physical,female,31.0% | sexual and physical abuse |
| 205 | MacMillan et al (2001) | US | 15-64 | 15-64 | population sample | 7016 | self-completed | sexual,male,4.3% sexual,female,12.4% physical,male,29.9% physical,female,21.2% | sexual and physical abuse |
| 206 | Madu & Peltzer (2001) | South Africa | 14-30 | 14-30 | school pupils | 414 | self-completed | sexual,male,60.0% sexual,female,53.2% | child sexual abuse |
| 207 | Madu (2003) | South Africa | 15-47 | 15-47 | university/college students | 722 | self-completed | sexual,male,21.7% sexual,female,23.7% physical,nogender,14.5% emo/psych,nogender,26.9% | sexual, physical, emotional abuse. Highest % taken from a range of questions on each of these |
| 208 | Mahram et al (2013) | Iran | 9 to 13 and over | 9-13 | school pupils | 1028 | self-completed | physical,male,19.9% physical,female,15.8% emo/psych,male,32.3% emo/psych,female,29.2% neglect,male,20.2% neglect,female,18.4% | physical and emotional abuse and neglect |
| 209 | Mamun et al (2007) | Australia | 21 | 21 | singletons whose mothers were enrolled the first antenatal visit | 2571 | self-completed | sexual,nogender,15.2% | child sexual abuse. Took highest % from a range of questions. |
| 210 | Mann et al (2005) | US | 17 or older | 17-100 | First-degree relatives of persons with mood disorder who attempt suicide | 457 | interview | sexual,nogender,9.6% physical,nogender,17.8% | sexual and physical abuse |
| 211 | Martin et al (2004) | Australia | year 8,9, 10high school | 12-15 | school pupils | 2475 | self-completed | sexual,male,2.0% sexual,female,5.4% | sexual abuse |
| 212 | Martsolf (2004) | Haiti | 18 or older | 18-100 | individuals seated in a waiting area in a hospital | 258 | interview | sexual,male,52.4% sexual,female,20.6% physical,male,52.4% physical,female,21.8% emo/psych,male,31.0% emo/psych,female,14.1% neglect,male,73.8% neglect,female,42.4% | sexual, physical, emotional abuse, physical or emotional neglect. Highest % taken for physical or emotional neglect |
| 213 | Masho & Amhed (2007) | US | 18 and over | 18-100 | population sample | 1769 | self-completed | sexual,female,20.8% | sexual assault under 18 |
| 214 | Matsumoto et al (2004) | Japan | 15-34 | 15-34 | outpatients with habitual self-mutilation or general psychiatric outpatients or controls with no clinical issues | 65 | self-completed | physical,female,41.5% | child physical abuse |
| 215 | Matsumoto et al (2009) | Japan | 15-17 | 15-17 | juvenile adolescents and school pupils | 632 | self-completed | sexual,nogender,4.6% | sexual abuse. % presented for juvenile and non-juvenile adolescents and these added together and divided by two |
| 216 | Matsuura et al (2009) | Japan | 15-19 | 15-19 | juvenile females | 91 | self-completed | sexual,female,8.6% physical,female,27.2% emo/psych,female,23.8% | sexual, physical, psychological abuse |
| 217 | Matthews et al (2002) | US | average age 43 | 43 | lesbian and heterosexual women | 829 | self-completed | sexual,female,25.3% | childhood sexual abuse. |
| 218 | May-Chahal and Cawson (2005) | UK | 18-24 | 18-24 | population sample | 2869 | self-completed and interview | sexual,male,6.0% sexual,female,15.0% physical,male,15.0% physical,female,12.0% emo/psych,male,4.0% emo/psych,female,8.0% neglect,male,6.0% neglect,female,7.0% | physical abuse to an intermediate degree, emotional abuse, absence of care, absence of supervision, and sexual abuse involving contact |
| 219 | Mazzeo et al (2008) | US | mean age 20.15 for African-Americans and 19.59 for Europeans | 19.9 | university/college students | 604 | self-completed | sexual,female,16.1% physical,female,54.9% emo/psych,female,74.2% neglect,female,45.6% | sexual, physical, emotional abuse, physical or emotional neglect. % reported for European-American and African-American participants separately, added these together and divided by 2. Highest % taken for physical or emotional neglect |
| 220 | Mbagaya et al (2013) | Kenya, Zambia, Netherlands | 18-40 | 18-40 | university/college students | 862 | self-completed | physical, male, Kenya 48.0%  phys, male, Zambia 43.0%  phys, no gend, N’lands 3.0%  phys, female, Kenya 36.0%  phys, female, Zambia 36.0%  neglect, male, Kenya 62.0% neg, male, Zambia 54.0%  neg, no gend, N’lands 42.0%  neg, female, Kenya 56.0%  neg, female, Zambia 53.0% | physical abuse and neglect. |
| 221 | McCrann et al (2006) | Tanzania | 20-53 | 20-53 | university/college students | 486 | self-completed | sexual,male,25.0% sexual,female,31.0% | child sexual abuse |
| 222 | McNutt et al (2002) | US | 18-44 | 18-44 | women who attended primary care centres | 557 | interview | sexual,female,25.3% physical,female,21.5% | childhood physical and sexual abuse |
| 223 | Melander & Tyler (2010) | US | mean 21.45 | 21.5 | homeless youth or youth with a history of homelessness | 172 | interview | sexual,nogender,47.1% | sexual abuse |
| 224 | Menard et al (2004) | US | 18-24 | 18-24 | young adults, recruited to the study when they were school pupils | 1715 | self-completed | sexual,male,15.3% sexual,female,18.4% physical,male,26.3% physical,female,21.5% emo/psych,male,27.4% emo/psych,female,26.5% | sexual, physical, emotional abuse |
| 225 | Messman-Moore & Brown (2004) | US | 18-22 | 18-22 | university/college students | 944 | self-completed | sexual,female,8.9% physical,female,4.2% emo/psych,female,8.6% | sexual, physical, emotional abuse |
| 226 | Messman-Moore (2000) | US | mean age 19.74 | 19.7 | university/college students | 648 | self-completed | sexual,female,20.1% | child sexual abuse |
| 227 | Mimiaga et al (2009) | US | 16 years or older | 16-100 | men who sleep with men | 4295 | self-completed | sexual,nogender,39.7% | child sexual abuse |
| 228 | Molnar et al (2001) | US | 15-54 | 15-54 | population sample | 5877 | interview | sexual,male,2.5% sexual,female,13.5% | child sexual abuse |
| 229 | Moran et al (2004) | US | 15-18 | 15-18 | School pupils | 2164 | self-completed | sexual,nogender,5.5% physical,nogender,10.6% emo/psych,nogender,9.5% | emotional, sexual and physical abuse |
| 230 | Morris & Balsam (2003) | US | 15-83 | 15-83 | lesbian and bisexual women | 2431 | self-completed | sexual,female,39.3% physical,female,30.8% | sexual assault and physical abuse |
| 231 | Moskvina et al (2007) | UK | 20-82 | 20-82 | adults with unipolar depression | 324 | self-completed | sexual,male,15.7% sexual,female,24.7% physical,male,32.6% physical,female,20.0% emo/psych,male,46.1% emo/psych,female,52.3% neglect,male,65.2% neglect,female,59.1% | sexual, physical, emotional abuse, physical or emotional neglect. Highest % taken for physical or emotional neglect |
| 232 | Mowlds et al (2010) | UK | 22-74 | 22-74 | patients with bipolar disorder | 52 | interview | unspecified,male,9.5% unspecified,female,48.4% | sexual and physical abuse. BUT results do not separate types so coded as type of abuse 'unsure'. |
| 233 | Mullings et al (2000) | US | average age 32 | 32 | female prisoners | 500 | interview | sexual,female,26.0% | sexually mistreated, abused, or raped while growing up |
| 234 | Ndetei et al (2007) | Kenya | 12-26 | 12-26 | school pupils | 1110 | self-completed | sexual,nogender,16.5% physical,nogender,23.2% | physical abuse, sexual abuse |
| 235 | Nduna et al (2013) | South Africa | 15-26 | 15-26 | population sample | 2783 | interview | sexual,male,22.4% sexual,female,24.9% physical,male,90.5% physical, female,84.8% emo/psych,male,29.3% emo/psych,female,29.2% neglect,male,23.3% neglect,female,30.5% | Sexual, physical, emotional abuse, emotional neglect. Paper reports % for each item in scale, highest % taken for each type of abuse. n shown for depressed and not depressed, added together and divided by 2. |
| 236 | Nelson et al (2002) | Australia | mean age 29.9 | 29.9 | twins | 1991 | interview | sexual,male,5.4% sexual,female,16.7% | sexual abuse |
| 237 | Nelson et al (2006) | Australia | mean age 29.9 | 29.9 | twins | 6050 | interview | sexual,male,6.0% sexual,female,17.4% | child sexual abuse |
| 238 | Nemeroff et al (2003) | US | 18-75 | 18-75 | participants with chronic major depressive disorder | 681 | self-completed | sexual,nogender,16.4% physical,nogender,43.5% neglect,nogender,10.0% | sexual and physical abuse and neglect. n provided for participants in various drug and psychotherapy groups, added and divided by 3. |
| 239 | Nguyen et al (2009) | Vietnam | 12-18 | 12-18 | school pupils | 2591 | self-completed | sexual,male,21.0% sexual,female,18.5% physical,male,54.0% physical,female,41.6% emo/psych,male,36.3% emo/psych,female,42.5% neglect,male,24.9% neglect,female,33.4% | sexual, physical, emotional abuse and neglect |
| 240 | Nichols & Harlow (2004) | US | 36-45 | 36-45 | depressed and non-depressed women | 722 | self-completed | sexual,female,6.0% physical,female,17.0% | sexual and physical abuse |
| 241 | Nickel et al (2004) | Germany | mean age 41 | 41 | inpatients at a clinic for psychosomatic medicine and psychotherapy | 936 | self-completed | sexual,nogender,26.7% | child sexual abuse |
| 242 | Nicolaidis et al (2004) | US | 25-60 | 25-60 | women presenting at medical clinics | 174 | interview | sexual,female,39.0% physical,female,44.0% | sexual and physical abuse. Various sexual abuse types reported, highest % taken |
| 243 | Nicolaidis et al (2009) | US | 18-92 | 18-92 | women presenting at medical clinics | 380 | self-completed | sexual,female,29.0% physical,female,23.0% | sexual and physical childhood abuse |
| 244 | Niederberger (2002) | Switzerland | 20-40 | 20-40 | population sample | 890 | self-completed | sexual,female,39.8% | sexual abuse |
| 245 | Oaksford and Frude (2001) | Wales | 18-41 | 18-41 | university/college students | 213 | self-completed | sexual,female,13.0% | child sexual abuse |
| 246 | O'Leary et al (2003) | US | 18 or older | 18-100 | HIV positive men who have sex with men | 456 | self-completed | sexual,male,14.9% | child sexual abuse |
| 247 | Olsson et al (2000) | Nicaragua | 25-44 | 25-44 | population sample | 367 | self-completed | sexual,male,20.0% sexual,female,26.0% | sexual abuse in childhood |
| 248 | Oquendo et al (2005) | US | 25-79 | 25-79 | patients with and without PTSD | 221 | interview | sexual,nogender,30.5% physical,nogender,38.7% | sexual and physical abuse. Highest % taken for severity of abuse. n for patients with and without PTSD added and divided by 2 |
| 249 | Orozco et al (2008) | Mexico | 12-17 | 12-17 | non-institutionalised children resident in the Mexico City Metropolitan Area | 3005 | interview | sexual,male,2.3% sexual,female,9.7% physical,male,12.2% physical,female,14.2% | sexual abuse, Beaten up as a child by caregiver |
| 250 | Parillo et al (2001) | US | mean age 32 | 32 | Non-injecting female sex partners of male heterosexual intravenous drug users | 1490 | self-completed | sexual,female,33.6% | sexual abuse in childhood and adolescence involving penetration |
| 251 | Paul et al (2001) | US | 18 or older | 18-100 | men who sleep with men | 2881 | interview | sexual,male,20.6% | child sexual assault |
| 252 | Pavio and Cramer (2004) | US | mean age of 19 | 19 | university/college students | 470 | self-completed | sexual,male,11.8% sexual,female,19.0% physical,male,22.2% physical,female,15.7% emo/psych,male,30.0% emo/psych,female,37.5% neglect,male,16.0% neglect,female,14.6% | emotional, sexual and physical abuse, and emotional and physical neglect |
| 253 | Pereda et al (2015) | Spain | 12-17 | 12-17 | school pupils | 1105 | self-completed | sexual,nogender,8.8% | sexual victimisation |
| 254 | Pérez-Fuentes et al (2013) | US | 18 and over | 18-100 | population sample | 34653 | interview | sexual,nogender,10.1% | child sexual abuse |
| 255 | Peschers et al (2003) | Germany | 14-87 | 14-87 | women attending an outpatients gynaecological clinic | 1075 | self-completed | sexual,female,33.8% | sexual abuse in childhood |
| 256 | Pluck et al (2011) | UK | 18-53 | 18-53 | homeless adults | 55 | interview | sexual,nogender,27.8% physical,nogender,53.7% emo/psych,nogender,66.7% neglect,nogender,77.8% | sexual, physical, emotional abuse, emotional and physical neglect. Highest number taken from emotional and physical neglect. Various severity reported, highest % taken. |
| 257 | Priebe & Svedin (2009) | Sweden | mean age 18.15 | 18.2 | school pupils | 4339 | self-completed | sexual,male,15.2% sexual,female,54.7% | child sexual abuse. Various types reported, highest % taken |
| 258 | Rada (2014) | Romainia | 18-75 | 18-75 | population sample | 869 | interview | physical,nogender,53.7% emo/psych,nogender,35.0% | victims of family violence and witnessed violence between their parents |
| 259 | Radford et al (2011) Child | UK | 11-17 | 11-17 | population sample | 2275 | self-completed | sexual,male,0.7% sexual,female,2.2% physical,male,6.8% physical,female,6.9% emo/psych,male,5.5% emo/psych,female,8.0% neglect,male,14.8% neglect,female,11.8% ma | emotional abuse, physical violence, sexual abuse, neglect (split by 2 tables by within and outside the family, highest % taken from each table) |
| 260 | Radford et al (2011) Adult | UK | 18-24 | 18-24 | population sample | 1761 | self-completed | sexual,male,11.4% sexual,female,14.3% physical,male,7.0% physical,female,9.9% emo/psych,male,6.2% emo/psych,female,9.6% neglect,male,5.6% neglect,female,6.4% ma | emotional abuse, physical violence, sexual abuse, neglect (split by 2 tables by within and outside the family, highest % taken from each table) |
| 261 | Ramiro et al (2010) | Philippines | mean 46.7 | 46.7 | population sample | 1068 | self-completed | sexual,male,4.5% sexual,female,6.0% physical,male,1.1% physical,female,1.5% emo/psych,male,26.4% emo/psych,female,19.3% neglect,male,44.5% neglect,female,42.8% | sexual, physical, emotional abuse and emotional and physical neglect. Highest % taken for emotional and physical neglect |
| 262 | Ramos et al (2004) | US | 18-44 | 18-44 | women from a primary health care setting | 491 | interview | sexual,female,11.6% physical,female,10.8% | sexual and physical abuse.n reported for White and African-American participants separately, added these together and divided by 2. Highest n taken for severity of abuse. |
| 263 | Rayburn et al (2005) | US | 18-55 | 18-55 | women who lived in shelters and low-income housing | 810 | interview | sexual,female,29.8% physical,female,31.0% | sexual and physical abuse |
| 264 | Rich-Edwards et al (2010) | US | 25-42 | 25-42 | adult nurses with diabetes | 7843 | self-completed | physical,female,54.0% | physical abuse in childhood or adolescence |
| 265 | Riley et al (2010) | US | 25-44 | 25-44 | Nurses | 68505 | self-completed | sexual,female,33.0% physical,female,53.0% | sexual and physical abuse |
| 266 | Ritchie et al (2009) | France | 65-92 | 65-92 | population sample | 942 | interview | emo/psych,nogender,5.6% neglect,nogender,5.5% unspecified,nogender,1.9% | sexual and physical abuse. BUT results do not separate types so coded as type of abuse 'unsure'. Neglect. Verbal abuse and Humiliation, harassment or mental cruelty taken as emotional abuse, highest % taken. |
| 267 | Roberts et al (2013) | US | from age 20 | 20-100 | population sample | 34296 | self-completed | sexual,male,2.2% sexual,female,7.0% unspecified,male,38.4% unspecified,female,34.4% | sexual abuse and non-sexual maltreatment. Various severity reported, highest % taken, and people who have ever and not ever had same sex partners n added together and divided by 2 |
| 268 | Robohm et al (2003) | US | 18-23 | 18-23 | lesbian and bisexual women | 227 | self-completed | sexual,female,37.9% | child sexual abuse |
| 269 | Rohde et al (2008) | US | mean age 52 | 52 | women enrolled in a large health plan | 4641 | interview | sexual,female,15.4% physical,female,12.3% | child sexual and physical abuse. |
| 270 | Romans et al (2002) | New Zealand | 26-70 | 26-70 | population sample | 354 | interview | sexual,female,48.9% | child sexual abuse |
| 271 | Rosenberg et al (2005) | US | 13-18 | 13-18 | population sample | 16644 | self-completed | sexual,nogender,7.0% physical,nogender,11.0% | sexual and physical assault |
| 272 | Rosenman and Rogers (2004) | Australia | 20-64 | 20-64 | population sample | 7485 | self-completed | sexual,nogender,1.1% physical,nogender,8.2% emo/psych,nogender,6.5% neglect,nogender,1.6% | sexual abuse, physical abuse and punishment (took the highest %of the two), verbal abuse and humiliation/mental cruelty (emotional abuse) (took the highest %of the two), neglect |
| 273 | Ross et al (2005) | China | average age 39.5 | 39.5 | inpatients and outpatients at a Mental Health Centre, and a non-clinical sample of workers at a clothing manufacturing factory | 1345 | interview | sexual,nogender,1.6% physical,nogender,4.8% | child physical and sexual abuse |
| 274 | Runtz (2002) | Canada | 17-56 | 17-56 | university/college students | 775 | self-completed | sexual,female,18.5% physical,female,19.7% | child sexual abuse, child physical maltreatment |
| 275 | Sar et al (2004) | Turkey | 16-56 | 16-56 | patients with conversion disorder | 38 | interview | sexual,nogender,26.3% physical,nogender,44.7% emo/psych,nogender,34.2% neglect,nogender,57.9% | sexual, physical, emotional abuse, emotional and physical and overall neglect. Highest number taken from emotional, physical and overall neglect. |
| 276 | Sar et al (2013) | Turkey | mean 34.8 | 34.8 | depressive women in the general population | 619 | interview | sexual,female,2.4% physical,female,9.0% emo/psych,female,8.9% neglect,female,20.7% | child sexual, physical and emotional abuse. Medical, emotional, and economic neglect, and deficiency of security and deficiency of nutrition (all taken to be neglect). Highest % taken for medical, emotional, and economic neglect and deficiency of security and deficiency of nutrition. Numbers reported with and without early cessation of education separately, added these together |
| 277 | Schein et al (2000) | Israel | 18-55 | 18-55 | adults presenting for routine health care at family practice clinics | 1005 | self-completed | sexual,male,15.7% sexual,female,30.7% | child sexual abuse |
| 278 | Scher et al (2004) | US | 18-65 | 18-65 | population sample | 967 | interview | sexual,male,2.2% sexual,female,7.5% physical,male,21.0% physical,female,17.1% emo/psych,male,9.6% emo/psych,female,14.3% neglect,male,22.1% neglect,female,14.2% | emotional abuse, emotional neglect, physical abuse, physical neglect, and sexual abuse |
| 279 | Schoemaker et al (2002) | Netherlands | 18-45 | 18-45 | participants with bulimia nervosa, psychiatric problems, substance misuse, dual diagnoses, and none of these issues | 1987 | interview | sexual,female,7.8% physical,female,4.2% emo/psych,female,11.8% neglect,female,18.3% | sexual, physical, psychological abuse and neglect. % reported for participants with various conditions reported separately, calculated the numbers these should be, added together and divided by total to give % |
| 280 | Schultz et al (2006) | US | 18-88 | 18-88 | women veterans and civilian community members | 223 | self-completed | sexual,female,46.6% | child sexual abuse |
| 281 | Scott et al (2008) | Colombia, Belgium, France, Germany, Italy, Netherlands, Spain, Japan, Mexico, US | 21-98 | 21-98 | adults with and without asthma | 18952 | interview | sexual,nogender,2.6% physical,nogender,9.8% neglect,nogender,6.6% | sexual and physical abuse and neglect. % reported participants with and without asthma, calculated the numbers these should be, added together and divided by total to give % |
| 282 | Seedat et al (2004) South Africa | South Africa | 14-22 | 14-22 | school pupils | 1140 | self-completed | sexual,male,15.0% sexual,female,12.0% sexual,nogender,14.0% | sexual assault |
| 283 | Seedat et al (2004) Kenya | Kenya | 14-22 | 14-22 | school pupils | 901 | self-completed | sexual,male,24.0% sexual,female,14.0% sexual,nogender,18.0% | sexual assault |
| 284 | Sesar et al (2008) | Croatia | 15-20 | 15-20 | School pupils | 458 | self-completed | sexual,male,21.0% sexual,female,13.0% physical,nogender,52.0% emo/psych,nogender,77.0% neglect,nogender,30.0% | emotional abuse, physical abuse, neglect, witnessing family violence, sexual abuse |
| 285 | Shen (2008) | Taiwan | 16-40 | 16-40 | University/college students | 1924 | self-completed | physical,nogender,6.0% | child physical maltreatment |
| 286 | Shen et al (2009) | Taiwan | 16-40 | 16-40 | university/college students | 1924 | self-completed | physical,nogender,11.8% emo/psych,nogender,15.9% | child physical maltreatment and interparental violence (taken to be emotional/psychological abuse).Many items reported, highest % taken. |
| 287 | Silvern et al (2000) | US | no age provided, college students | 18-100 | university/college students | 542 | self-completed | sexual,male,4.4% sexual,female,16.5% physical,male,26.5% physical,female,19.6% | child sexual and physical abuse both researcher and self-defined, took the highest % |
| 288 | Slonim-Nevo & Mukuka (2007) | Zambia | 10-19 | 10-19 | population sample of adolescents | 3360 | self-completed | sexual,male,7.0% sexual,female,10.0% physical,male,23.0% physical,female,24.0% | sexual and physical abuse. Took highest % from a series of questions. |
| 289 | So-kum Tang (2002) | China | 18-25 | 18-25 | university/college students | 2147 | self-completed | sexual,male,4.3% sexual,female,7.4% | child sexual abuse |
| 290 | Sørbø et al (2013) | Norway | 14 to over 35 | 14-35 | population sample based on a pregnancy cohort | 65393 | self-completed | sexual,female,7.0% physical,female,6.0% emo/psych,female,14.0% | sexual, physical, emotional child abuse |
| 291 | Spertus et al (2003) | US | 19-82 | 19-82 | women who presented in a hospital based primary care practice | 205 | self-completed | sexual,female,8.9% physical,female,5.4% emo/psych,female,19.5% neglect,female,22.4% | sexual, physical, emotional abuse and emotional neglect. Highest % taken for varying severity presented. |
| 292 | Springer (2009) | US | mean age 53 | 53 | population sample | 3317 | self-completed | physical,nogender,11.9% | physical abuse |
| 293 | Springer et al (2007) | US | mean age 55 | 55 | siblings of men and women who graduated in 1957 from Wisconsin high schools | 2051 | self-completed | physical,nogender,11.4% | child physical abuse |
| 294 | Steel & Herlitz (2005) | Sweden | 23-79 | 23-79 | population sample | 2810 | self-completed and interview | sexual,male,5.6% sexual,female,13.9% | childhood or adolescent sexual abuse |
| 295 | Stein et al (2002) | US | 16-50 | 16-50 | homeless adults | 581 | interview | sexual,female,36.0% physical,female,31.0% emo/psych,female,49.0% | sexual and physical abuse and verbal abuse. Verbal abuse taken to be emotional/psychological abuse |
| 296 | Stenson et al (2003) | Sweden | 16-43 | 16-43 | women registered at antenatal clinics | 1038 | self-completed | sexual,female,2.0% | child sexual abuse before 13 |
| 297 | Stephenson et al (2006) | Iran | 11-18 | 11-18 | school pupils | 1370 | self-completed | physical,nogender,74.0% emo/psych,nogender,67.2% neglect,nogender,67.3% | Physical and mental maltreatment and neglect. Average of % of abuse taken in home and school used |
| 298 | Stoddard et al (2009) | US | 40 and older | 40-100 | lesbians and their heterosexual sisters | 648 | self-completed | sexual,female,20.8% physical,female,15.0% | childhood sexual and physical abuse |
| 299 | Strine et al (2012) | US | 18-over 75 | 18-75 | managed care population | 7279 | self-completed | sexual,male,16.7% sexual,female,24.1% physical,male,28.3% physical,female,24.4% emo/psych,male,8.2% emo/psych,female,11.7% | sexual, physical, emotional abuse |
| 300 | Subica (2013) | US | 18-84 | 18-84 | participants with schizophrenia-spectrum, bipolar, or recurrent major depressive disorders | 172 | interview | sexual,nogender,29.1% physical,nogender,44.8% | child sexual and physical abuse |
| 301 | Suija et al (2011) | Estonia | 18-75 | 18-75 | patients with depression | 123 | self-completed | unspecified,nogender,68.0% | childhood abuse, does not specify type. |
| 302 | Sun et al (2008) | China | 18-25 | 18-25 | University/college students | 1307 | self-completed | sexual,male,14.7% sexual,female,22.1% | child sexual abuse |
| 303 | Sun et al (2012) | China | 30-60 | 30-60 | melancholic versus nonmelancholic patients with major depression | 1970 | interview | sexual,female,9.6% | childhood sexual abuse. Numbers reported for women with and without melancholia separately, added these together |
| 304 | Sung et al (2013) | US | mean 37.6 for early-onset MDD patients and 46.8 for adult-onset MDD patients | 42.2 | outpatients non-psychotic chronic major depressive episode or recurrent major depressive disorder | 663 | self-completed | sexual,nogender,21.7% physical,nogender,19.6% emo/psych,nogender,39.1% neglect,nogender,35.9% | sexual, physical, emotional abuse and neglect. Numbers reported for early and adult onset separately, added these together |
| 305 | Swahn & Bossarte (2007) | US | 14-18 | 14-18 | School pupils | 13,639 | self-completed | sexual,male,3.8% sexual,female,10.7% | sexual assault |
| 306 | Tang (2002) | China | 18-30 | 18-30 | University/college students | 2147 | self-completed | sexual,male,4.3% sexual,female,7.4% | child sexual abuse |
| 307 | Thakkar et al (2000) | US | mean age 18.74 | 18.7 | university/college students | 707 | self-completed | sexual,female,13.4% physical,female,14.7% | childhood contact sexual abuse and physical abuse |
| 308 | The NIMH Multisite HIV Prevention Trial Group (2001) | US | 18-65 | 18-65 | population sample | 3336 | interview | sexual,female,38.5% | Unwanted sexual activity during childhood |
| 309 | Thompson et al (2000) | US | 18-64 | 18-64 | women who presented at a hospital for non-fatal suicide attempts and controls who presented for non-emergency medical problems | 335 | interview | sexual,female,44.5% physical,female,59.0% emo/psych,female,49.5% neglect,female,48.0% | sexual, physical, emotional, emotional and physical neglect. Highest % taken for emotional and physical neglect |
| 310 | Thompson et al (2002) | US | 18 and over | 18-100 | population sample | 8000 | interview | unspecified,female,86.0% | child sexual and physical victimisation. BUT results do not separate types so coded as type of abuse 'unsure' |
| 311 | Thompson et al (2004) | US | 18 and over | 18-100 | population sample | 16000 | interview | physical,male,53.8% physical,female,40.0% | physical abuse. % reported for many items, chose 'experienced any violence' |
| 312 | Thurman et al (2006) | South Africa | 14-18 | 14-18 | population sample | 1694 | interview | sexual,male,0.6% sexual,female,7.4% | forced to have sex/raped |
| 313 | Tietjen et al (2010) | US and Canada | mean age 41 | 41 | patients seeking treatment in headache centres | 1348 | self-completed | sexual,nogender,8.1% physical,nogender,9.0% emo/psych,nogender,17.7% neglect,nogender,19.1% | sexual, physical, emotional abuse, physical or emotional neglect. Highest % taken for physical or emotional neglect. Highest % taken for severity |
| 314 | Timko et al (2008) | US | 18 and older | 18-100 | population sample | 6942 | self-completed | sexual,female,11.2% physical,female,19.1% emo/psych,female,18.4% | emotional abuse and physical and sexual assault |
| 315 | Tomeo et al (2001) | US | 18-68 | 18-68 | gay and heterosexual men and women | 942 | self-completed | sexual,male,33.7% sexual,female,29.0% | sexual molestation |
| 316 | Tourigny et al (2008) | Canada | 18 and over | 18-100 | population sample | 1002 | interview | sexual,nogender,16.0% physical,nogender,19.0% emo/psych,nogender,22.0% | physical, sexual and psychological violence against children |
| 317 | Tran et al (2015) | Vietnam | 18-30 | 18-30 | university/college students | 2099 | self-completed | sexual,male,14.9% sexual,female,15.2% physical,male,44.9% physical,female,34.9% emo/psych,male,44.0% emo/psych,female,40.6% neglect,male,9.3% neglect,female,7.2% | sexual, physical, emotional abuse and physical neglect |
| 318 | Tran et al (2016) | Vietnam | 12-17 | 12-17 | school pupils | 1851 | self-completed | sexual,male,8.9% sexual,female,5.3% physical,male,42.8% physical,female,34.6% emo/psych,male,57.5% emo/psych,female,62.6% neglect,male,22.6% neglect,female,27.2% | both emotional abuse and Witnessed parental conflict measured and so highest % was taken as a measure of emotional/psychological abuse |
| 319 | Trent et al (2007) | US | mean age 19.9 | 19.9 | US Navy recruits | 5697 | self-completed | sexual,male,22.5% sexual,female,48.5% physical,male,43.7% physical,female,51.7% | sexual and physical abuse |
| 320 | van der Kooij et al (2015) adult | Suriname | 18-22 | 18-22 | school pupils and those on vocational courses | 239 | self-completed | sexual,male,31.8% sexual,female,24.4% physical,male,63.6% physical,female,62.9% emo/psych,male,68.2% emo/psych,female,62.9% neglect,male,61.7% neglect,female,59.8% | sexual abuse, physical abuse, neglect, for psychological/emotional abuse authors used 2 measures 'Psychological aggression of parents & Experienced conflicts between parents' - took highest % |
| 321 | van der Kooij et al (2015) child | Suriname | 12-17 | 12-17 | school pupils and those on vocational courses | 1072 | self-completed | sexual,male,21.2% sexual,female,20.3% physical,male,51.0% physical,female,55.1% emo/psych,male,47.7% emo/psych,female,57.1% neglect,male,51.6% neglect,female,49.9% | sexual abuse, physical abuse, neglect, for psychological/emotional abuse authors used 2 measures 'Psychological aggression of parents & Experienced conflicts between parents' - took highest % |
| 322 | Vander Weg (2011) | US | 18 to over 65 | 18-65 | population sample | 10227 | interview | sexual,nogender,9.3% physical,nogender,12.2% emo/psych,nogender,28.8% | Touched sexually, forced to touch adult sexually, forced to have sex, victim of physical assault, victim of verbal abuse, household physical assault. 3 items for sexual abuse, taken highest. Household physical assault and victim of verbal abuse taken to be emotional/psychological abuse, highest taken |
| 323 | Von Korff et al (2009) | Colombia, Belgium, France, Germany, Italy, Netherlands, Spain, Japan, Mexico, US | adults, no age data | 18-100 | adults with and without arthritis | 18309 | interview | sexual,nogender,2.5% physical,nogender,9.6% neglect,nogender,6.5% | sexual and physical abuse and neglect |
| 324 | Wainwright & Surtees (2002) | UK | 17-77 | 17-77 | population sample | 3353 | self-completed | physical,nogender,3.6% | physical abuse |
| 325 | Wan & Leung (2010) | China | 11-18 | 11-18 | school pupils | 2754 | self-completed | physical,nogender,9.8% | history of physical abuse |
| 326 | Welles et al (2009) | US | 18 years or older | 18-100 | HIV positive men who have sex with men | 593 | self-completed | sexual,male,47.0% | childhood sexual abuse |
| 327 | Wiersma et al (2009) | Netherlands | mean 40.7 | 40.7 | participants with and without anxiety or depressive disorders | 1204 | interview | sexual,nogender,16.7% physical,nogender,11.0% emo/psych,nogender,28.5% neglect,nogender,44.5% | sexual, physical, emotional abuse, emotional neglect. Highest % taken for frequency. % reported for non-chronic and chronic MDD separately, added these together |
| 328 | Wilhelm et al (2002) | Australia | mean 44.1 for men and 42.9 for women | 43.5 | patients with major depression | 270 | interview | sexual,male,5.1% sexual,female,20.5% | sexual abuse. Split by parent and by other, highest % taken |
| 329 | Wilsnack et al (2008) | US | 21-40 | 21-40 | lesbian and heterosexual women | 953 | interview | sexual,female,34.8% | childhood sexual abuse. % presented for many groups indicating sexual orientation, numbers calculated from % and these added together and divided by number of groups |
| 330 | Wilsnack et al (2012) | US | 18 and older | 18-100 | Lesbian and heterosexual women | 1328 | self-completed | sexual,female,33.5% | childhood sexual abuse. |
| 331 | Wise et al (2001) | US | 36-45 | 36-45 | population sample | 732 | self-completed | sexual,female,9.0% physical,female,30.0% | sexual and physical abuse |
| 332 | Wise et al (2011) | US | 21-69 | 21-69 | convenience sample of African American women | 35728 | self-completed | sexual,female,18.0% physical,female,42.0% | sexual and physical abuse |
| 333 | Yen et al (2008) (a) | Taiwan | 13-8 | 13-18 | school pupils | 2079 | self-completed | sexual,male,3.0% sexual,female,2.0% physical,male,21.9% physical,female,22.5% | sexual and physical abuse |
| 334 | Yen et al (2008) (b) | Taiwan | 13-18 | 13-18 | school pupils | 1684 | self-completed | physical,nogender,22.3% | physical abuse |
| 335 | Yoshihama & Horrocks (2010) | Japan | 18-49 | 18-49 | population sample | 1371 | interview | sexual,female,10.4% | child sexual abuse |
| 336 | Yoshinaga et al (2004) | Japan | 14-19 | 14-19 | incarcerated juvenile delinquents | 251 | self-completed | sexual,male,2.9% sexual,female,6.7% physical,male,15.0% physical,female,8.9% | sexual and physical assault |
| 337 | Young et al (2006) | US | 18-20 | 18-20 | men starting military training | 41482 | self-completed | sexual,male,1.7% physical,male,2.8% emo/psych,male,13.2% neglect,male,17.1% | sexual abuse, physical abuse, emotional abuse, physical and emotional neglect, witnesses domestic violence. Highest % taken for physical and emotional neglect, and emotional abuse and witnessed domestic violence. |
| 338 | Young et al (2008) | US | 12-18 | 12-18 | School pupils | 1086 | self-completed | sexual,male,26.6% sexual,female,48.4% | sexual assault victimization experiences |
| 339 | Ystgaard et al (2004) | Norway | 16-82 | 16-82 | Patients who had taken an overdose or deliberately injured themselves | 74 | interview | sexual,nogender,35.1% physical,nogender,17.6% neglect,nogender,27.0% | sexual and physical abuse and neglect |
| 340 | Zanarini et al (2002) | US | 18-35 | 18-35 | borderline inpatients | 290 | interview | sexual,nogender,62.4% neglect,nogender,92.1% unspecified,nogender,86.2% | sexual abuse and neglect. 'Other' forms of abuse (not sexual) also reported so listed as unsure |
| 341 | Zhao et al (2010) | China | 6-18 | 6-18 | children who had lost one or both parents to HIV, other vulnerable children, and comparison children | 1019 | self-completed OR interview | sexual,male,37.4% sexual,female,24.1% | child sexual abuse |
| 342 | Zlotnick et al (2001) | US | 18-65 | 18-65 | patients with major depression | 235 | interview | sexual,nogender,24.7% | sexual abuse |
| 343 | Zoroglu et al (2003) | Turkey | 12-17 | 12-17 | school pupils | 839 | self-completed | sexual,male,6.7% sexual,female,13.3% physical,male,14.6% physical,female,12.1% emo/psych,male,15.6% emo/psych,female,16.1% neglect,male,12.9% neglect,female,18.9% | childhood physical, sexual and emotional abuse, and neglect |
