# Supplementary material for: Establishing the international prevalence of self-reported child maltreatment: a systematic review by maltreatment type and gender
Source: BMC Public Health. 2018 Oct 10;18:1164. doi: 10.1186/s12889-018-6044-y (PMC6180456; doi:10.1186/s12889-018-6044-y)
Supplement: Supplementary file 3 — References for table (additional file 2) containing data for each of the studies included in review. (DOCX 47 kb) [file 12889_2018_6044_MOESM3_ESM.docx]

**Additional file 3. References for table (additional file 2) containing data for each of the studies included in review**

Aaron, D. J., & Hughes, T.L. (2007). Association of childhood sexual abuse with obesity in a community sample of lesbians. Obesity, 15, 1023-1028.

Aberle, N., Ratkovic-Blazevic, V., Mitrovic-Dittrich, D., Coha, R., Stoic, A. et al. (2007). Emotional and physical abuse in family: survey among high school adolescents. Croat Med J, 42, 240-248.

Afifi, Z. E. M., Basuily, W. W., El-Lawindi, M. I., & Ahmed, S.A. (2003). Adolescent abuse in a community sample in Beni Suef, Egypt: Prevalence and risk factors. Eastern Mediterranean Health

Journal, 9, 1003-1018.

Afifi, T.O., Brownridge, D.A., Cox, B.J., & Sareen, J. (2006). Physical punishment, childhood abuse and psychiatric disorders. Child Abuse and Neglect, 30(10), 1093-103.

Afifi, T.O., Mota, N.P., Dasiewicz, P., Macmillan, H.L., & Sareen, J. (2012). Physical punishment and mental disorders: results from a nationally representative, U.S sample. Pediatrics, 130, 1–9.

Akyuz, G., Sar, V., Kugu, N., & Dogan, O. (2005). Reported childhood trauma, attempted suicide and self-mutilative behaviour among women in the general population. European Psychiatry, 20, 268-273.

Alami, M., & Kadri, N. (2004). Moroccan women with a history of child sexual abuse and its long-term repercussions: A population-based epidemiological study. Archives of Women's Mental Health, 7, 237−242.

Alikasifoglu, M., Erginoz, E., Ercan, O., Albayrak-Kaymak, D., Uysal, O., & Ilter, O. (2006). Sexual abuse among female high school students in Istanbul, Turkey. Child Abuse and Neglect, 30, 247–255.

Allard, C. (2009). Prevalence and sequelae of betrayal trauma in a Japanese student sample. Psychological Trauma: Theory, Research, Practice, and Policy, 1(1), 65-77.

Almeida, O.P., Alfonso, H., Pirkis, J., Kerse, N., Sim, M. et al. (2011). A practical approach to assess depression risk and to guide risk reduction strategies in later life. Int Psychogeriatr, 2011, 23(2), 280-91.

Ammerman, R.T., Putnam, F.W., Chard, K.M., Stevens, J., & van Ginkel, J.B. (2012). P.TSD in depressed mothers in home visitation. Psychol Trauma, 4(2), 186-95.

Amodeo, M., Griffin, M. L., Fassler, I. R., Clay, C. M., & Ellis, M.A. (2006). Childhood sexual abuse among black women and white women from two-parent families. Child Maltreatment, 11, 237-246.

Angst, J., Gamma, A., Rössler, W., Ajdacic, V., & Klein, D.N. (2011). Childhood adversity and chronicity of mood disorders. Eur Arch Psychiatry Clin Neurosci, 261(1), 21-7.

Annerbäck, E.M., Wingren, G., Svedin, C.G., & Gustafsson, P.A. (2010). Prevalence and characteristics of child physical abuse in Sweden - findings from a population-based youth survey. Acta Paediatr, 99(8), 1229-36.

Ansara, D., Cohen, M. M., Gallop, R., Kung, R., & Schei, B. (2005). Predictors of women’s physical health problems after childbirth. Journal of Psychosomatic Obstetrics and Gynecology, 26, 115-125.

Appel, K., Schwahn, C., Mahler, J., Schulz, A., Spitzer, C. et al. (2011). Moderation of adult depression by a polymorphism in the FKBP5 gene and childhood physical abuse in the general population. [Neuropsychopharmacology](https://www.ncbi.nlm.nih.gov/pmc/articles/PMC3158316/), 36(10), 1982–1991.

Arata, C.M., Langhinrichsen-Rohling, J., Bowers, D., & O'Farrill-Swails, L. (2005). Single versus multi-type maltreatment: An examination of the long-term effects of child abuse. J Aggress Maltreat Trauma, 11(4), 29-52.

Ariga, M., Uehara, T., Takeuchi, K., Ishige, Y., Nakano, R. & Mikuni, M. (2008). Trauma exposure and posttraumatic stress disorder in delinquent female adolescents. The Journal of Child Psychology and Psychiatry, 49(1), 79-87.

Arreola, S. G., Neilands, T., Pollack, L., Paul, J., & Catania, J. (2008). Childhood sexual experiences and adult health sequelae among gay and bisexual men: Defining childhood sexual abuse. Journal of Sex Research, 45, 246–252.

Arreola, S. G., Neilands, T. B., & Diaz, R. (2009). Childhood sexual abuse and the sociocultural context of sexual risk among adult Latino gay and bisexual men. American Journal of Public Health, 99, 432–438.

Arreola, S. G., Neilands, T. B., Pollack, L. M., Paul, J. P., & Catania, J.A. (2005). Higher prevalence of childhood sexual abuse among Latino men who have sex with men than non-Latino men who have sex with men: data from the Urban Men's Health Study. Child Abuse and Neglect, 29, 285−290.

Aslund, C., Nilsson, K.W., Starrin, B., & Sjoberg, R.L. (2007). Shaming experiences and the association between adolescent depression and psychosocial risk factors. Eur Child Adolesc Psychiatry 16, 298–304.

Aspelmeier, J. E., Elliot, A. N., & Smith, C.H. (2007). Childhood sexual abuse, attachment, and trauma symptoms in college females: The moderating role of attachment. Child Abuse and Neglect, 31, 549-566.

Audu, B., Geidam, A., & Jarma, H. (2009). Child labor and sexual assault among girls in Maiduguri, Nigeria. Int J Gynecol Obstet 104, 64–67.

Baccini, F., Pallota, N., Calabrese, E., Pezzotti, P., & Corazziari, E. (2003). Prevalence of sexual and physical abuse and its relationship with symptom manifestations in patients with chronic organic and functional gastrointestinal disorders. Digestive and Liver Disease, 35, 256-261.

Back, S. E., Jackson, J. L., Fitzgerald, M., Shaffer, A., Salstrom, S., & Osman, M.M. (2003). Child sexual and physical abuse among college students in Singapore and the United States. Child Abuse and Neglect, 27, 1259−1275.

Bailey, K., Webster, R., Baker, A.L., & Kavanagh, D.J. (2012). Exposure to dysfunctional parenting and trauma events and posttraumatic stress profiles among a treatment sample with coexisting depression and alcohol use problems. Drug Alcohol Rev, 31(4), 529-37.

Balsam, K.F., Lehavot, K., Beadnell, B., & Circo, E. (2010). Childhood abuse and mental health indicators among ethnically diverse lesbian, gay, and bisexual adults. J Consult Clin Psychol, 78(4), 459-68.

Bandelow, B., Krause, J., Wedekind, D., Broocks, A., Hajak, G., & Rüther, E. (2005). Early traumatic life events, parental attitudes, family history, and birth risk factors in patients with borderline personality disorder and healthy controls. Psychiatry Res, 134(2), 169-79.

Banerjee, S.R., Bharati, P., Vasulu, T.S., Chakrabarty, S., & Banerjee, P. (2008). Whole time domestic child labor in metropolitan city of Kolkata. Indian Pediatr, 45, 579–582

Banou, E., Hobfoll, S.E., & Trochelman, R.D. (2009). Loss of resources as mediators between interpersonal trauma and traumatic and depressive symptoms among women with cancer. J Health Psychol, 14(2), 200-14.

Barney, D.D. (2003). Health Risk-Factors for Gay American Indian and Alaska Native Adolescent Males. J Homosex, 46(1-2), 137-57.

Bebbington, P.E., Jonas, S., Brugha, T., Meltzer, H., Jenkins, R., Cooper, C., King, M., & Mcmanus, S. (2011). Child sexual abuse reported by an English national sample, characteristics and demography. Soc Psychiatry Psychiatr Epidemiol, 46(3), 255-62.

Bensley, L.S., Van Eenwyk, J., & Simmons, K.W. (2000). Self-reported childhood sexual and physical abuse and adult HIV-risk behaviors and heavy drinking. Am J Prev Med, 18, 151–158.

Bensley, L. S., Van Eenwyk, J., Wynkoop Simmons, K. (2003). Childhood family violence history and women’s risk for intimate partner violence and poor health. Am J Prev Med, 25, 38-44.

Berliner, L., Fine, D., & Moore, D. (2001). Sexual assault experiences and perceptions of community response to sexual assault: A survey of Washington state women. Olympia, W.A., Office of Crime Victims Advocacy.

Bifulco, A., Bernazzani, O., Moran, P.M., & Ball, C. (2000). Lifetime stressors and recurrent depression, Preliminary findings of the adult life phase interview (ALPHI). Soc Psychiatry Psychiatr Epidemiol, 35(6), 264-75.

Birdthistle, I.J., Floyd, S., Machingura, A., Mudziwapasi, N., Gregson, S., & Glynn, J.R. (2008). From affected to infected? Orphanhood and HIV risk among female adolescents in urban Zimbabwe. AIDS, 22, 759–766.

Blain, L.M., Muench, F., Morgenstern, J., & Parsons, J.T. (2012). Exploring the role of child sexual abuse and posttraumatic stress disorder symptoms in gay and bisexual men reporting compulsive sexual behavior. Child Abuse and Neglect, 36(5), 413-22.

Bohn, D., Bernardy, K., Wolfe, F., & Häuser, W. (2013). The association among childhood maltreatment, somatic symptom intensity, depression, and somatoform dissociative symptoms in patients with fibromyalgia syndrome: A single-center cohort study. J Trauma Dissociation, 14(3), 342-58.

Bonomi, A.E., Cannon, E.A., Anderson, M.L., Rivara, F.P., & Thompson, R.S. (2008). Association between selfreported health and physical and/or sexual abuse experienced before age 18. Child Abuse and Neglect, 32(7), 693-701.

Boynton-Jarrett, R., Rosenberg, L., Palmer, J.R., Boggs, D.A., & Wise, L.A. (2012). Child and adolescent abuse in relation to obesity in adulthood: The black women’s health study. Pediatr, 130(2), 245-53.

Bradley, R.G., Binder, E.B., Epstein, M.P., Tang, Y., Nair, H.P. et al. (2008). Influence of child abuse on adult depression: moderation by the corticotropin-releasing hormone receptor gene. Arch Gen Psychiatry, 65(2), 190-200.

Brand, S.R., Brennan, P.A., Newport, D.J., Smith, A.K., Weiss, T.E., & Stowe, Z.N. (2010). The impact of maternal childhood abuse on maternal and infant: HPA axis function in the postpartum period. Psychoneuroendocrinology, 35(5), 686-93.

Brennan, D. J., Hellerstedt, W. L., Ross, M. W., & Welles, S.L. (2007). History of childhood sexual abuse and HIV risk behaviors in homosexual and bisexual men. American Journal of Public Health, 97, 1107–1112

Brezo, J., Paris, J., Vitaro, F., Hebert, M., Tremblay, R.E., et al. (2008). Predicting suicide attempts in young adults with histories of childhood abuse. Br J Psychiatry, 193, 134–139.

Briere, J., & Elliott, D.M. (2003). Prevalence and psychological sequelae of self-reported childhood physical and sexual abuse in a general population sample of men and women. Child Abuse and Neglect, 27, 1205−1222.

Brodsky, B.S., Oquendo, M.A., Ellis, S.P., Haas, G.L., Malone, K.M., & Mann, J.J. (2001). The relationship of childhood abuse to impulsivity and suicidal behavior in adults with major depression. Am J Psychiatry, 158(11), 1871-7.

Brooker, S., Cawson, P., Kelly, G., & Wattam, C. (2001). The prevalence of child abuse and neglect: A survey of young people. International Journal of Market Research, 43, 249-289.

Brown, J., Berenson, K., & Cohen, P. (2005). Documented and selfreported child abuse and adult pain in a community sample. Clinical Journal of Pain, 21, 374-377.

Brown, G.W., Ban, M., Craig, T.KJ, Harris, T.O., Herbert, J., Uher, R. (2013). Serotonin transporter length polymorphism, childhood maltreatment, and chronic depression: A specific gene-environment interaction. Depress Anxiety, 30(1), 5-13.

Cawson, P., Wattam, C., Brooker, S., & Kelly, G. (2000). Child maltreatment in the United Kingdom: A study of the prevalence of child abuse and neglect. London, NSPCC.

Champion, J.D., Kelly, P., Shain, R.N., & Piper, J.M (2004). Rural Mexican–American adolescent sexual risk behavior. J Rural Health 20, 279–285.

Chapman, D. P., Whitfield, C. L., Felitti, V. J., Dube, S. R., Edwards, V. J., & Anda, R.F. (2004). Adverse childhood experiences and the risk of depressive disorders in adulthood. Journal of Affective Disorders, 82, 217–225.

Chartier, M. J., Walker, J. R., & Naimark, B. (2007). Childhood abuse, adult health, and health care utilization: Results from a representative community sample. American Journal of Epidemiology, 165, 1031-1038.

Chartier, M.J., Walker, J.R., & Naimark, B. (2009). Health risk behaviors and mental health problems as mediators of the relationship between childhood abuse and adult health. Am J Public Health, 99, 847–854.

Chen, J., Dunne, M.P., & Han, P. (2004). Child sexual abuse in China, a study of adolescents in four provinces. Child Abuse and Neglect, 28, 1171–1186.

Chen, J., Dunne, M.P., & Han, P. (2006). Child sexual abuse in Henan province, China, associations with sadness, suicidality, and risk behaviors among adolescent girls. J Adolesc Health, 38, 544–549.

Cheng-Fang, Y., Mei-Sang, Y., Ming-Jen, Y., Yi-Chinig, S., Mei-Hua, W., & Chu-Mei, L. (2008). Childhood physical and sexual abuse, prevalence and correlates among adolescents living in rural Taiwan. Child Abuse and Neglect, 32, 429–438.

Clemmons, J. C., Dilillo, D., Martinez, I. G., Degue, S., & Jeffcott, M. (2003). Co-occurring forms of child maltreatment and adult adjustment reported by Latina college students. Child Abuse and Neglect, 27, 751-767.

Cohen, P., Brown, J., & Smailes, E. (2001). Child abuse and neglect and the development of mental disorders in the general population. Dev Psychopathol, 13, 981–999.

Cohen, R. A., Paul, R. H., Stroud, L., Gunstad, J., Hitsman, B. L. et al. (2006). Early life stress and adult emotional experience: An international perspective. International Journal of Psychiatry in Medicine, 36, 35–52.

Coid, J., Petruckevitch, A., Chung, W. S., Richardson, J., Moorey, S., & Feder, G. (2003). Abusive experiences and psychiatric morbidity in women primary care attenders. British Journal of Psychiatry, 183, 332-339.

Collishaw, S., Pickles, A., Messer, J., Rutter, M., Shearer, C., & Maughan, B. (2007). Resilience to adult psychopathology following childhood maltreatment: Evidence from a community sample. Child Abuse and Neglect, 31, 211-229.

Collin-Vézina, D., Cyr, M., Pauzé, R., & Mcduff, P. (2005). The role of depression and dissociation in the link between childhood sexual abuse and later parental practices. J Trauma Dissociation, 6(1), 71-97.

Comijs, H.C., van Exel, E., van der Mast, R.E., Paauw, A., Voshaar, R.O., & Stek, M.L. (2013). Childhood abuse in late-life depression. J Affect Disord, 147(1-3), 241-6.

Cong, E., Li, Y., Shao, C., Chen, J., Wu, W. et al. (2012). Childhood sexual abuse and the risk for recurrent major depression in Chinese women. Psychol Med, 42(2), 409-17.

Conroy, E., Degenhardt, L., Mattick, R.P., & Nelson, E.C. (2009). Child maltreatment as a risk factor for opioid dependence, comparison of family characteristics and type and severity of child maltreatment with a matched control group. Child Abuse and Neglect, 33, 343–352.

Corliss, H.L., Cochran, S.D., & Mays, V.M. (2002). Reports of parental maltreatment during childhood in a United States population-based survey of homosexual, bisexual, and heterosexual adults. Child Abuse and Neglect, 26(11), 1165-78.

Cyr, K., Chamberland, C., Clément, M. È., Lessard, G., Wemmers, J. A. et al. (2013). Polyvictimization and victimization of children and youth: Results from a populational survey. Child Abuse and Neglect, 37(10), 814-820.

Dalenberg, C. J., & Palesh, O. G. (2004). Relationships between child abuse history, trauma, and dissociation in Russian college students. Child Abuse and Neglect, 28, 461-474.

Danese, A., Moffitt, T.E., Pariante, C.M., Ambler, A., Poulton, R., & Caspi, A. (2008). Elevated inflammation levels in depressed adults with a history of childhood maltreatment. Arch Gen Psychiatry, 65(4), 409-15.

De Von Figueroa-Moseley, C. D., Landrine, H., & Klonoff, E. A. (2004). Sexual abuse and smoking among college student women. Addictive Behaviors, 29, 245-251.

Decker, M.R., Raj, A., & Silverman, J.G (2007b). Sexual violence against adolescent girls: Influences of immigration and acculturation. Violence Against Woman, 13, 498–513.

Decker, M.R., Mccauley, H.L., Phuengsamran, D., Janyam, S., Seage, I.II, G.R. & Silverman, J.G. (2010). Violence victimisation, sexual risk and sexually transmitted infection symptoms among female sex workers in Thailand. Sexually Transmitted Infections, 86, 236-240.

Dennis, M.F., Flood, A.M., Reynolds, V., Araujo, G., Calncy, C.P. et al. (2009). Evaluation of lifetime trauma exposure and physical health in women with posttraumatic stress disorder or major depressive disorder. Violence Against Women, 15(5), 618-27.

Deyessa, N., Berhane, Y., Alem, A., Ellsberg, M., Emmelin, M. et al. (2009). Intimate partner violence and depression among women in rural Ethiopia: A cross-sectional study. Clin Pract Epidemiol

Ment Health, 5, 8.

Diaz, A., Simantov, E., & Rickert, V.I. (2002). Effect of abuse on health, results of a national survey. Arch Pediatr Adolesc Med, 156(8), 811-7.

Diaz-Olavarrieta, C., Paz, F., de la Cadena, C. G., & Campbell, J. (2001). Prevalence of intimate partner abuse among nurses and nurses’ aides in Mexico. Archives of Medical Research, 32, 79-87.

Dibble, S. L., Sato, N., & Haller, E. (2007). Asians and native Hawaiian or other Pacific Islanders midlife lesbians’ health: A pilot study. Women & Therapy, 30, 129-143.

Dietz, T.L. (2009). Drug and alcohol use among homeless older adults: Predictors of reported current and lifetime substance misuse problems in a national sample. Journal of Applied Gerontology, 28(2), 235-255.

Dolezal, C., & Carballo-Diéguez, A. (2002). Childhood sexual experiences and the perception of abuse among Latino men who have sex with men. Journal of Sex Research, 39, 165–173.

Dong, M., Anda, R.F., Felitti, V.J., Dube, S.R., Williamson, D.F., Thompson, T.J., Loo, C.M., & Giles, W.H. (2004). The interrelatedness of multiple forms of childhood abuse, neglect, and household dysfunction. Child Abuse and Neglect, 28(7), 771-84.

Draper, B., Pfaff, J.J., Pirkis, J., Snowdon, J., Lautenschlager, N.T. et al. (2008). Long-term effects of childhood abuse on the quality of life and health of older people, results from the depression and early prevention of suicide in general practice project. J Am Geriatr Soc 56, 262–271.

Dube, S.R., Anda, R.F., Felitti, V.J., Chapman, D.P., Williamson, D.F. et al. (2001). Childhood abuse, household dysfunction, and the risk of attempted suicide throughout the life span, findings from the adverse childhood experiences study. Journal of the American Medical Association, 286, 3089–3096.

Dube, S.R., Felitti, V.J., Dong, M., Chapman, D.P., Giles, W.H. et al. (2003). Childhood abuse, neglect, and household dysfunction and the risk of illicit drug use, the adverse childhood experiences study. Pediatrics, 111, 564–572.

Duke, N.N., Pettingell, S.L., mcmorris, B.J., & Borowsky, I.W (2010). Adolescent violence perpetration, associations with multiple types of adverse childhood experiences. Pediatrics, 125, 778–786.

Duncan, R. D. (2000). Childhood maltreatment and college drop-out rates: Implications for child abuse researchers. Journal of Interpersonal Violence, 15, 987-995.

Dunkle, K. L., Jewkes, R. K., Brown, H. C., Yoshihama, M., Gray, G. E., Mcintyre, J. A., & Harlow, S. D. (2004). Prevalence and patterns of gender-based violence and revictimization among women attending antenatal clinics in Soweto, South Africa. American Journal of Epidemiology, 160, 230-239.

Dunne, M. P., Purdie, D. M., Cook, M. D., Boyle, F. M., & Najman, J. M. (2003). Is child sexual abuse declining? Evidence from a population-based survey of men and women in Australia. Child Abuse and Neglect, 27, 141-152.

Duran, B., Malcoe, L. H., Sanders, M., Waitzkin, H., Skipper, B., & Yager, J. (2004). Child maltreatment prevalence and mental disorders outcomes among American Indian women in primary care. Child Abuse and Neglect, 28, 131-145.

Dussich, J.P.J., & Maekoya, C. (2007). Physical child harm and bullying-related behaviors: A comparative study in Japan, South Africa, and the United States. International Journal of Offender Therapy and Comparative Criminology, 51(5), 495-509.

Edgardh, K., & Ormstad, K. (2000). Prevalence and characteristics of sexual abuse in a national sample of Swedish seventeen-year-old boys and girls. Acta Paediatrica, 88, 310−319.

Edwards, V.J., Holden, G.W., Felitti, V.J., & Anda, R.F. (2003). Relationship between multiple forms of childhood maltreatment and adult mental health in community respondents: Results from the Adverse Childhood Experiences Study. Am J Psychiatry, 160(8), 1453-60.

Eisenberg, M.E., Ackard, D.M., & Resnick, M.D (2007). Protective factors and suicide risk in adolescents with a history of sexual abuse. J Pediatr, 151, 482–487.

Enns, M.W., Cox, B.J., Afifi, T.O., De Graaf, R., Ten Have, M. et al. (2006). Childhood adversities and risk for suicidal ideation and attempts, a longitudinal population-based study. Psychol Med 36, 1769–1778.

Eskin, M., Kaynak-Demir, H., & Demir, S. (2005). Same-sex sexual orientation, childhood sexual abuse, and suicidal behavior in university students in Turkey. Arch Sex Behav, 34(2), 185-95.

Evans-Campbell, T., Lindhorst, T., Huang, B., & Walters, K.L (2006). Interpersonal violence in the lives of urban American Indian and Alaska Native women: Implications for health, mental health, and help-seeking. Am J Public Health, 96, 1416–1422.

Evren, C., Evren, B. (2005). Self-mutilation in substance-dependent patients and relationship with childhood abuse and neglect, alexithymia and temperament and character dimensions of personality. Drug Alcohol Depend, 80, 15–22.

Evren, C., Kural, S., & Cakmak, D. (2006). Clinical correlates of childhood abuse and neglect, in substance dependents. Addict Behav, 31(3), 475-85.

Everson, M.D., Smith, J.B., Hussey, J.M., English, D., Litrownik, A.J., Dubowitz, H., Thompson, R., Dawes Knight, E., & Runyan, D.K. (2008). Concordance between adolescent reports of childhood abuse and Child Protective Service determinations in an at-risk sample of young adolescents. Child Maltreat, 13(1), 14-26.

Fakhari, A., Tabatabavakili, M., Javid, Y.S., & Farhang, S. (2012). Family violence influences mental health of school girls in Iran: Results of a preliminary study. Asian J Psychiatr, 5(1), 24-7.

Fanslow, J. L., Robinson, E. M., Crengle, S., & Perese, L. (2007). Prevalence of child sexual abuse reported by a cross-sectional sample of New Zealand women. Child Abuse and Neglect, 31, 935−945.

Feldman, M. B., & Meyer, I. H. (2007). Childhood abuse and eating disorders in gay and bisexual men. International Journal of Eating Disorders, 40, 418-423.

Feng, J.Y., Chang, Y.T., Chang, H.Y., Fetzer, S., & Wang, J.D. (2015). Prevalence of different forms of child maltreatment among Taiwanese adolescents: A population-based study. Child Abuse and Neglect, 42, 10-9.

Fergusson, D. M., Horwood, J. L., & Woodward, L. J. (2000). The stability of child abuse reports: A longitudinal study of the reporting behaviour of young adults. Psychological Medicine, 30, 529−544.

Fergusson, D.M., Boden, J.M., & Horwood, L. (2008). Exposure to childhood sexual and physical abuse and adjustment in early adulthood. Child Abuse and Neglect, 32(6), 607-19.

Figueiredo, B., Bifulco, A., Paiva, C., Maia, A., Fernandes, E., & Matos, R. (2004). History of childhood abuse in Portuguese parents. Child Abuse and Neglect, 28, 669−682.

Fillingim, R. B., & Edwards, R. R. (2005). Is self-reported childhood abuse history associated with pain perception among healthy young women and men? Clinical Journal of Pain, 21, 387-397.

Finkelhor, D., Shattuck, A., Turner, H.A., & Hamby, S.L. (2014). The lifetime prevalence of child sexual abuse and sexual assault assessed in late adolescence. J Adolesc Health, 55(3), 329-33.

Finkelhor, D., Turner, H.A., Shattuck, A., & Hamby, S.L. (2015). Prevalence of childhood exposure to violence, crime, and abuse: Results from the National Survey of Children's Exposure to Violence. Journal of the American Medical Association, Pediatr, 169(8), 746-54.

Fisher, H.L., Craig, T.K., Fearon, P., Morgan, K., & Dazzan, P. et al. (2011). Reliability and comparability of psychosis patients’ retrospective reports of childhood abuse. Schizophr Bull, 37(3), 546-53.

Fisher, H.L., Cohen-Woods, S., Hosang, G.M., Korszum, A., Owen, M. et al. (2013). Interaction between specific forms of childhood maltreatment and the serotonin transporter gene (5-HTT) in

Recurrent Depressive Disorder. J Affect Disord, 145(1), 136-41.

Flynn-O'Brien, K.T., Rivara, F.P., Weiss, N.S., Lea, V.A., Marcelin, L.H., et al. (2016). Prevalence of physical violence against children in Haiti, A national population-based cross-sectional survey. Child Abuse and Neglect, 51, 154-62.

Fogarty, C.T., Fredman, L., Heeren, T.C., & Liebschutz, J. (2008). Synergistic effects of child abuse and intimate partner violence on depressive symptoms in women. Prev Med, 46(5), 463-9.

Fricker, A. E., Smith, D. W., Davis, J. L., & Hanson, R. F. (2003). Effects of context and question type on endorsement of childhood sexual abuse. Journal of Traumatic Stress, 16, 265-268.

Friedman, S., Smith, L., Fogel, D., Paradis, C., Viswanathan, R. et al. (2002). The incidence and influence of early traumatic life events in patients with panic disorder: A comparison with other psychiatric outpatients. J Anxiety Disord, 16(3), 259-72.

Friedman, M. S., Marshal, M. P., Stall, R., Cheong, J., & Wright, E. R. (2008). Gay-related development, early abuse and adult health outcomes among gay males. AIDS & Behavior, 12, 891-902.

Fuemmeler, B.F., Dedert, E., mcclernon, F.J., & Beckham, J.C (2009). Adverse Childhood events are associated with obesity and disordered eating, results from a U.S. population-based survey of young adults. J Trauma Stress 22, 329–333.

Fujiwara, T., Okuyama, M., Izumi, M. & Osada, Y. (2010)(b). The impact of childhood abuse history and domestic violence on the mental health of women in Japan. Child Abuse and Neglect, 34, 267-274.

Fujiwara, T., Kawakami, N., & World Mental Health Japan Survey Group (2011). Association of childhood adversities with the first onset of mental disorders in Japan, results from the World Mental Health Japan, 2002–2004. J Psychiatr Res, 45, 481–487.

Fuller-Thomson, E., Baker, T.M., & Brennenstuhl S (2010). Investigating the association between childhood physical abuse and migraine. Headache, 50, 749–760.

Gagne´, M. H., Lavoie, F., & Herbert, M. (2005). Victimization during childhood and revictimization in dating relationships in adolescent Girls. Child Abuse and Neglect, 29, 1155-1172.

Gallagher, B., Bradford, M., & Pease, K. (2002). The sexual abuse of children by strangers: Its extent, nature and victims’ characteristics. Children and Society, 16, 346–359.

Gamble, S.A., Talbot, N.L., Conner, K.R., Tu, X., Franus, N. et al. (2007). Concordance about childhood sexual abuse among depressed patients 50 and over and their family and friends. Arch Suicide Res, 11(4), 321-6.

Garcia, J., Adams, J., Friedman, L., & East, P. (2002). Links between past abuse, suicide ideation, and sexual orientation among San Diego college students. Journal of American College Health, 51,

9-14.

Garcia-Moreno, C., Jansen, H.A.F.M., Ellsberg, M., Heise, & L., Watts, C. WHO multi-country study on women’s health and domestic violence against women. Initial results on prevalence, health outcomes and women’s responses. Geneva, World Health Organization, 2005.

Gaudiano, B.A., & Zimmerman, M. (2010). The relationship between childhood trauma history and the Psychotic subtype of major depression. Acta Psychiatr Scand, 121(6), 462-70.

Gault-Sherman, M., Silver, E., & Sigfúsdóttir, I.D. (2009). Gender and the associated impairments of childhood sexual abuse, a national study of Icelandic youth. Soc Sci Med, 69(10), 1515-22.

Gerke, C.K., Mazzeo, S.E., & Kliewer, W. (2006). The role of depression and dissociation in the relationship between childhood trauma and bulimic symptoms among ethnically diverse female undergraduates. Child Abuse and Neglect, 30(10), 1161-72.

Gibb, B.E., Butler, A.C., & Beck, J.S. (2003). Childhood abuse, depression, and anxiety in adult psychiatric outpatients. Depress Anxiety, 17(4), 226-8.

Gladstone, G.L., Parker, G.B., Mitchell, P.B., Malhi, G.S., Wilhelm, K., & Austin, M. (2004). Implications of childhood trauma for depressed women: an analysis of pathways from childhood sexual abuse to deliberate self-harm and revictimization. Am J Psychiatry, 161(8), 1417-25.

Goodwin, R.D., & Stein, M.B. (2004). Association between childhood trauma and physical disorders among adults in the United States. Psychol Med, 34, 509–520.

Goodwin, R.D., Hoven, C.W., Murison, R., & Hotopf, M. (2003). Association between childhood physical abuse and gastrointestinal disorders and migraine in adulthood. Am J Public Health, 93, 1065–1067.

Goodwin, R.D., Fergusson, D.M., & Horwood, L.J. (2005). Childhood abuse and familial violence and the risk of panic attacks and panic disorder in young adulthood. Psychol Med, 35, 881–890.

Gratz, K.L., Conrad, S.D., & Roemer, L. (2002). Risk factor for deliberate self-harm among college students. American Journal of Orthopsychiatry, 72, 128–40.

Grayson, C. E., & Nolen-Hoeksema, S. (2005). Motives to drink as mediators between childhood sexual assault and alcohol problems in adult women. Journal of Traumatic Stress, 18, 137-145.

Green, J.G., Mclaughlin, K.A., Berglund, P.A., Gruber, M.J., Sampson, N.A. et al. (2010). Childhood adversities and adult psychiatric disorders in the national comorbidity survey replication, associations with first onset of DSM-IV disorders. Arch Gen Psychiatry, 67, 113–123.

Groleau, P., Steiger, H., Bruce, K., Israel, M., Sycz, L. et al. (2012). Childhood emotional abuse and eating symptoms in bulimic disorders: In examination of possible mediating variables. Int J Eat Disord, 45(3), 326-32.

Grote, N.K., Spieker, S.J., Lohr, M., Geibel, S.L., Swartz, H.A. et al. (2012). Impact of childhood trauma on the outcomes of a perinatal depression trial. Depress Anxiety, 29(7), 563-73.

Gunn, J.M., Gilchrist, G.P., Chondros, P., Ramp, M., Hegarty, K.L. et al. (2008). Who is identified when screening for depression is undertaken in general practice? Baseline findings from the

Diagnosis, management and outcomes of depression in primary care (diamond) longitudinal study.

Med J Aust, 188, 119-25.

Gunnlaugsson, G., Kristjánsson, A.L., Einarsdóttir, J., & Sigfúsdóttir, I.D. (2011). Intrafamilial conflict and emotional well-being, a population based study among Icelandic adolescents. Child Abuse and Neglect, 35(5), 372-81.

Gwadz, M.V., Nish, D., Leonard, N.R., & Strauss, S.M. (2007). Gender differences in traumatic events and rates of post-traumatic stress disorder among homeless youth. J Adolesc, 30(1), 117-29.

Hamburger, M.E., Leeb, R.T., & Swahn, M.H (2008). Childhood maltreatment and early alcohol use among high-risk adolescents. J Stud Alcohol Drugs 69, 291–295.

Hamelin, C., Salomon, C., Sitta, R., Gueguen, A., Cyr, A. & Lert, F. (2009). Childhood sexual abuse and adult binge drinking among Kanak women in New Caledonia. Social Science & Medicine, 68, 1247-1253.

Handa, M., Nukina, H., Hosoi, M., & Kubo, C. (2008). Childhood physical abuse in outpatients with psychosomatic symptoms. Biopsychosoc Med, 2, 8.

Hanson, R.F., Saunders, B., Kilpatrick, D., Resnick, H., Crouch, J.A. et al. (2001). Impact of childhood rape and aggravated assault on adult mental health. Am J Orthopsychiatry, 71, 108–119.

Harkness, K.L., & Monroe, S.M. (2002). Childhood adversity and the endogenous versus nonendogenous distinction in women with major depression. Am J Psychiatry, 159(3), 387-93.

Harkness, K.L., Bagby, R.M., & Kennedy, S.H. (2012). Childhood maltreatment and differential treatment response and recurrence in adult major depressive disorder. J Consult Clin Psychol, 80(3), 342-53.

Harrison, P.A., & Narayan G (2003). Differences in behavior, psychological factors, and environmental factors associated with participation in school sports and other activities in adolescence. J Sch Health, 73, 113–120.

Hasnain, N., & Kumar D (2006). Psychological well-being of women reporting sexual abuse in childhood. J Indian Acad Appl Psychol, 32, 15–19

Hegarty, K., Gunn, J., Chondros, P., & Small, R. (2004). Association between depression and abuse by partners of women attending general practice, Descriptive, cross sectional survey. BMJ, 328(7440), 621-4.

Heidt, J. M., Marx, B. P., & Gold, S. D. (2005). Sexual revictimization among sexual minorities, A preliminary study. Journal of Traumatic Stress, 18, 533-540.

Helweg-Larsen, K., & Boving Larsen, H. (2006). The prevalence of unwanted and unlawful sexual experiences reported by Danish adolescents, results from a national youth survey in 2002. Acta

Paediatr, 95, 1270–1276.

Henny, K.D., Kidder, D.P., Stall, R., & Wolitski, R.J. (2007). Physical and sexual abuse among homeless and unstably housed adults living with HIV: Prevalence and associated risks. AIDS Behav, 11(6), 842-

53.

Hester, M., He, J., & Lan, L. (2009). Girls’ and Boys’ experiences and perceptions of parental discipline and punishment while growing up in China and England. Child Abuse Review, 18(6), 401-413.

Hetzel, M. D., & Mccanne, T. R. (2005). The roles of peritraumatic dissociation, child physical abuse, and child sexual abuse in the development of posttraumatic stress disorder and adult victimization.

Child Abuse and Neglect, 29, 915-930.

Hill, J., Davis, R., Byatt, M., Burnside, E., Rollinson, L., & Fear, S. (2000). Childhood sexual abuse and affective symptoms in women: A general population study. Psychological Medicine, 30, 1283-1291.

Hillis, S.D., Anda, R.F., Felitti, V., Nordenberg, D., & Marchbanks, P. (2000). Adverse childhood experiences and sexually transmitted diseases in men and women, a retrospective study. Pediatrics, 106, 11.

Hovens, J.GFM, Wiersma, J.E., Giltay, E.J., van Oppen, P., Spinhoven, P. et al. (2010). Childhood life events and childhood trauma in adult patients with depressive, anxiety and comorbid disorders vs. controls. Acta Psychiatr Scand, 122, 66–74.

Hovens, J.G., Giltay, E.J., Wiersma, J.E., Spinhoven, P., Penninx, B.W., & Zitman, F.G. (2012). Impact of childhood life events and trauma on the course of depressive and anxiety disorders. Acta Psychiatr Scand, 126(3), 198-207.

Howard, D. E., & Wang, M. Q. (2005). Psychosocial correlates of, U.S adolescents who report a history of forced sexual intercourse. Journal Of Adolescent Health, 36, 372-379.

Huang, S., Trapido, E., Fleming, L., Arheart, K., Crandall, L. et al. (2011). The longterm effects of childhood maltreatment experiences on subsequent illicit drug use and drug-related problems in young adulthood. Addict Behav, 36, 95–102.

Hughes, T. L., Haas, A. P., Razzano, L., Cassidy, R., & Matthews, A. (2000). Comparing lesbians’ and heterosexual women’s mental health, A multi-site survey. Journal of the Gay and Lesbian Social

Services, 11, 57-76.

Hughes, T. L., Johnson, T., & Wilsnack, S. C. (2001). Sexual assault and alcohol abuse, comparison of lesbians and heterosexual women. Journal of Substance Abuse, 13, 515–532.

Hussey, J.M., Chang, J.J., & Kotch, J.B. (2006). Child maltreatment in the United States, prevalence, risk factors, and adolescent health consequences. Pediatrics, 118(3), 933-42.

Jewkes, R., Levin, J., Mbananga, N., & Bradshaw, D. (2002). Rape of girls in South Africa. Lancet, 359, 319-320.

Jewkes, R.K., Dunkle, K., Nduna, M., Jama, P.N., & Puren, A. (2010). Associations between childhood adversity and depression, substance abuse and HIV and HSV2 incident infections in rural South African youth. Child Abuse and Neglect, 34, 833–841.

Jirapramukpitak, T., Prince, M., & Harpham, T. (2005). The experience of abuse and mental health in the young Thai population—A preliminary survey. Social Psychiatry and Psychiatric Epidemiology,

40, 955-963.

Jirapramukpitak, T., Harpham, T. & Prince, M. (2011). Family violence and its ‘adversity package’, a community of family violence and adverse mental outcomes among young people. Social Psychiatry and Pyschiatric Epidemiology, 46(9), 825–831.

Johnson, R. J., Ross, M. W., Taylor, W. C., Williams, M. L., Carvajal, R. I., & Peters, R. J. (2006). Prevalence of childhood sexual abuse among incarcerated males in county jail. Child Abuse

And Neglect, 30, 75-86.

Johnstone, J.M., Luty, S.E., Carter, J.D., Mulder, R.T., Frampton, C.M.A, & Joyce, P.R. (2009). Childhood neglect and abuse as predictors of antidepressant response in adult depression. Depress Anxiety, 26(8), 711-7.

Johnstone, J.M., Carter, J.D., Luty, S.E., Mulder, R.T., Frampton, C.M., & Joyce, P.R. (2013). Maternal care and paternal protection influence response to psychotherapy treatment for adult depression. J Affect Disord, 149(1-3), 221-9.

Joyce, P.R., Mckenzie, J.M., Luty, S.E., Mulder, R.T., Carter, J.D. et al. (2003). Temperament, childhood environment and psychopathology as risk factors for avoidant and borderline personality disorders. Aust N Z J Psychiatry, 37(6), 756-64.

Ju, S., & Lee, Y. (2010). Experiences of family maltreatment by Korean children in Korean National Protective Services. Child Abuse and Neglect, 34, 18-27.

Jumaian, A. (2001). Prevalence and long-term impact of child sexual abuse among a sample of male college students in Jordan. Eastern Mediterranean Health Journal, 7(3), 435−440.

Jun, H.J., Rich-Edwards, J.W., Boynton-Jarrett, R., Austin, S.B., Frazier, A.L. et al. (2008). Child abuse and smoking among young women, the importance of severity, accumulation, and timing. J Adolesc Health, 43, 55–63.

Karayianni, E., Fanti, K.A., Diakidoy, I.A., Hadjicharalambous, M.Z., & Katsimicha, E. (2017). Prevalence, contexts, and correlates of child sexual abuse in Cyprus. Child Abuse and Neglect, 66, 41-52.

Keeshin, B.R. & Campbell, K. (2011). Screening homeless youth for histories of abuse: prevalence, enduring effects, and interest in treatment. Child Abuse and Neglect, 35, 401– 407.

Kendler, K. S., Bulik, C. M., Silberg, J., Hettema, J. M., Myers, J., & Prescott, C. A. (2000). Childhood sexual abuse and adult psychiatric and substance use disorders in women—An epidemiological and Cotwin control analysis. Archives of General Psychiatry, 57, 953-959.

Kenny, M., & Mceachern, A.G. (2000a). Prevalence and characteristics of childhood sexual abuse in multiethnic female college students. Journal of child sexual abuse, 9, 57-70.

Kerr, T., Stoltz J-A., Marshall, B.D.L, Lai, C., Strathdee, S.A. et al. (2009). Childhood trauma and injection drug use among high-risk youth. J Adolesc Health 45, 300–302.

Khamis, V. (2000). Child psychological maltreatment in Palestinian families. Child Abuse and Neglect, 24, 1047-1059.

Kilpatrick, D. G., Acierno, R., Saunders, B., Resnick, H. S., & Best, C. L. (2000). Risk factors for adolescent substance abuse and dependence, Data from a national sample. Journal of Consulting

And Clinical Psychology, 68, 19-30.

Kim, H.S., & Kim, H.S (2005). Incestuous experience among Korean adolescents, prevalence, family problems, perceived family dynamics, and psychological characteristics. Publ Health Nurs, 22, 472–482.

Kim, J., Park, S. & Emery, C.R. (2009). The incidence and impact of family violence on mental health among South Korean women, Results of a national survey. Journal of Family Violence, 24, 193-202.

King, G., Flisher, A. J., Noubary, F., Reece, R., Marais, A., & Lombard, C. (2004). Substance abuse and behavioral correlates of sexual assault among South African adolescents. Child Abuse and Neglect, 28, 683-696.

Kitamura, T., Kaibori, Y., Takara, N., Oga, H., Yamauchi, K. & Fujihara, S. (2000). Child abuse, other early experiences and depression: Edpidemiology of parental loss, child abuse, perceived rearing experiences and early life events among a Japanese community population. Archives of Women’s Mental Health, 3, 47-52.

Kong, S., & Bernstein, K. (2009). Childhood trauma as a predictor of eating psychopathology and its

Mediating variables in patients with eating disorders. J Clin Nurs, 18(13), 1897-907.

Kounou, K.B., Bui, E., Dassa, K.S., Hinton, D., Fischer, L. et al. (2013). Childhood trauma, personality disorders symptoms and current major depressive disorder in Togo. Soc Psychiatry Psychiatr Epidemiol, 48(7), 1095-103.

Kraaij, V., & de Wilde, E.J. (2001). Negative life events and depressive symptoms in the elderly: a life span perspective. Aging Ment Health, 5(1), 84-91.

Kvam, M. H. (2004). Sexual abuse of deaf children. A retrospective analysis of the prevalence and characteristics of childhood sexual abuse among deaf adults in Norway. Child Abuse and Neglect,

28, 241-251.

Leeners, B., Neumaier-Wagner, P., Quarg, A. F., & Rath, W. (2006). Childhood sexual abuse (CSA) experiences: An underestimated factor in perinatal care. Acta Obstetricia et Gynecologica Scandinavica, 85, 971-976.

Lehavot, K., Walters, K. L., & Simoni, J.M. (2009). Abuse, mastery, and health among lesbian, bisexual, and two-spirit American Indian and Alaska Native women. Cultural Diversity and Ethnic Minority Psychology, 15, 275–284.

Lepistö, S., Luukkaala, T., & Paavilainen, E. (2011). Witnessing and experiencing domestic violence, a descriptive study of adolescents. Scand J Caring Sci, 25(1), 70-80.

Lewis, R.J., Griffin, J.L., Winstead, B.A., Morrow, J.A., & Schubert, C.P. (2003). Psychological characteristics of women who do or do not report a history of sexual abuse. J Prev Interv Community, 26(1), 49-65.

Li, N., Ahmed, S., & Zabin, L.S. (2012). Association between childhood sexual abuse and adverse psychological outcomes among youth in Taipei. J Adolesc Health, 50, 45-51.

Libby, A.M., Orton, H.D., Novins, D.K., Beals, J., & Manson, S.M. (2005). Childhood physical and sexual abuse and subsequent depressive and anxiety disorders for two American Indian tribes. Psychol Med 2005, 35(3), 329-40.

Logan, J.E., Leeb, R.T., & Barker, L.E (2009). Gender-specific mental and behavioural outcomes among physically abused high-risk seventh-grade youths. Public Health Reports, 124, 234–245.

Lu, W., Mueser, K.T., Rosenberg, S.D., & Jankowski, M.K. (2008). Correlates of adverse childhood experiences among adults with severe mood disorders. Psychiatr Serv, 59(9), 1018-26.

Lutenbacher, M. (2000). Perceptions of health status and the relationship with abuse history and mental health in low-income single mothers. J Fam Nurs, 6(4), 320-40.

Macmillan, H.L., Fleming, J.E., Streiner, D.L., Lin, E., Boyle, M.H. et al. (2001). Childhood abuse and lifetime psychopathology in a community sample. Am J Psychiatry, 158(11), 1878-83.

Madu, S.N., & Peltzer, K. (2001). Prevalence and patterns of child sexual abuse and victim–perpetrator relationship among secondary school students in the Northern Province (South Africa). Archives of Sexual Behavior, 30(3), 311−321.

Madu, S. N. (2003). The relationship between parental physical availability and child sexual, physical and emotional abuse: A study among a sample of university students in South Africa. Scandinavian Journal of Psychology, 44, 311-318.

Mahram, M., Hosseinkhani, Z., Nedjat S., & Aflatouni, A. (2013). Epidemiologic evaluation of child abuse and neglect in school-aged children of Qazvin province, Iran. Iran J Pediatr, 23, 159-164.

Mamun, A., Alati, R., O’Callaghan, M., Hayatbakhsh, M. R., O’Callaghan, F. V. et al. (2007). Does childhood sexual abuse have an effect on young adults’ nicotine disorder (dependence or withdrawal)? Evidence from a birth Cohort study. Addiction, 102, 647-654.

Mann, J.J., Bortinger, J., Oquendo, M.A., Currier, D., Li, S., Brent, D.A. (2005). Family history of suicidal behavior and mood disorders in probands with mood disorders. Am J Psychiatry, 162(9), 1672-9.

Martin, G., Bergen, H.A., Richardson, A.S., Roeger, L., & Allison, S. (2004). Sexual abuse and suicidality: Gender differences in a large community sample of adolescents. Child Abuse and Neglect, 28, 491–593.

Martsolf, D.S. (2004). Childhood maltreatment and mental and physical health in Haitian adults. J Nurs Scholarsh, 36(4), 293-9.

Masho, S.W., & Ahmed, G. (2007). Age at sexual assault and posttraumatic stress disorder among women: Prevalence, correlates, and implications for prevention. Journal of Womens Health, 16,

262-271.

Matsumoto, T., Yamaguchi, A., Chiba, Y., Asami, T., Iseki, E. & Hirayasu, Y. (2004). Patters of self-cutting: preliminary study on differences in clinical implications between wrist- and arm-cutting using a Japanese juvenile detention center sample. Psychiatry and Clinical Neurosciences, 58, 377-382.

Matsumoto, T., Tsutsumi, A., Izutsu, T., Imamura, F., Chiba, Y. & Takeshima, T. (2009). Comparative stud of the prevalence of suicidal behavior and sexual abuse history in delinquent and non-delinquent adolescents. Psychiatry and Clinical Neurosciences, 63, 238-240.

Matsuura, N., Hashimoto, T. & Toichi, M. (2009). Correlations among self-esteem, aggression, adverse childhood experiences and depression in inmates of a female juvenile correctional facility in Japan. Psychiatry and Clinical Neurosciences, 63, 478-485.

Matthews, A.K., Hughes, T. L., Johnson, T., Razzano, L. A., & Cassidy, R. (2002). Prediction of depressive distress in a community Sample of women: The role of sexual orientation. American

Journal of Public Health, 92, 1131-1139.

May-Chahal, C., & Cawson, P. (2005). Measuring child maltreatment in the United Kingdom, A study of the prevalence of child abuse and neglect. Child Abuse and Neglect, 29, 969−984.

Mazzeo, S.E., Mitchell, K.S., & Williams, L.J. (2008). Anxiety, alexithymia, and depression as mediators of the association between childhood abuse and eating disordered behavior in African American and European American women. Psychol Women Q, 32(3), 267-80.

Mbagaya, C., Oburu, P., & Bakermans-Kranenburg, M.J. (2013). Child physical abuse and neglect in Kenya, Zambia and the Netherlands, a cross-cultural comparison of prevalence, psychopathological sequelae and mediation by PTSS. Int J Psychol, 48(2), 95-107.

Mccrann, D., Lalor, K., & Katabaro, J. K. (2006). Childhood sexual abuse among university students in Tanzania. Child Abuse and Neglect, 30, 1343−1351.

Mcnutt, L.A., Carlson, B.E., Persaud, M., & Postmus, J. (2002). Cumulative abuse experiences, physical health and health behaviors. Ann Epidemiol, 12(2), 123-30.

Melander, L.A., & Tyler, K.A (2010). The effect of early maltreatment, victimization, and partner violence on HIV risk behavior among homeless young adults. J Adolesc Health 47(2010), 575–581

Menard, C., Bandeen-Roche, K. J., & Chilcoat, H. D. (2004). Epidemiology of multiple childhood traumatic events, child abuse, parental psychopathology, and other family-level stressors. Social

Psychiatry and Psychiatric Epidemiology, 39, 857-865.

Messman-Moore, T.L., & Brown, A. L. (2004). Child maltreatment and perceived family environment as risk factors for adult rape: Is child sexual abuse the most salient experience. Child Abuse and

Neglect, 28, 1019-1034.

Messman-Moore, T. L. (2000). Child sexual abuse and revictimization in the form of adult sexual abuse, adult physical abuse, and adult psychological maltreatment. Journal of Interpersonal

Violence, 15, 489-502.

Mimiaga, M. J., Noonan, E., Donnell, D., Safren, S. A., Koenen, K. C. et al. (2009). Childhood sexual abuse is highly associated with HIV risk-taking behavior and infection among MSM in the EXPLORE study. Journal of Acquired Immune Deficiency Syndromes, 51, 340-348.

Molnar, B.E., Buka, S.L., & Kessler, R.C. (2001). Child sexual abuse and subsequent psychopathology, results from the National Comorbidity Survey. Am J Public Health, 91(5), 753-60.

Moran, P.B., Vuchinich, S., & Hall, N.K (2004). Associations between types of maltreatment and substance use during adolescence. Child Abuse and Neglect, 28, 565–574.

Morris, J. F., & Balsam, K. F. (2003). Lesbian and bisexual women’s experiences of victimization, mental health, revictimization, and sexual identity development. Journal of Lesbian Studies, 7, 67-85.

Moskvina, V., Farmer, A., Swainson, V., O'Leary, J., Gunasinghe, C. et al. (2007). Interrelationship of childhood trauma, neuroticism, and depressive phenotype. Depress Anxiety, 24(3), 163-8.

Mowlds, W., Shannon, C., Mccusker, C.G., Meenagh, C., Robinson, D. et al. (2010). Autobiographical memory specificity, depression, and trauma in bipolar disorder. Br J Clin Psychol, 49(2), 217-33.

Mullings, J.L., Marquart, J.W., & Brewer, V.E. (2000). Assessing the relationship between child sexual abuse and marginal living conditions on, HIV/AIDS-related risk behavior among women prisoners. Child Abuse and Neglect, 24(5), 677-88.

Ndetei, D.M., Ongecha-Owuor, F.A., Khasakhala, L., Mutiso, V., Odhiambo, G., & Kokonya, D.A (2007). Traumatic experiences of Kenyan secondary school students. J Child Adolesc Mental Health, 19, 147–155.

Nduna, M., Jewkes, R.K., Dunkle, K.L., Shai, N.P.J, & Colman, I. (2013). Prevalence and factors associated with depressive symptoms among young women and men in the Eastern Cape Province, South Africa. J Child Adolesc Ment Health, 25(1), 43-54.

Nelson, E.C., Heath, A.C., Madden, P.A., Cooper, M.L., Dinwiddie, S.H., Bucholz, K.K., Glowinski, A., Mclaughlin, T., Dunne, M.P., Statham, D.J., & Martin, N.G. (2002). Association between self-reported childhood sexual abuse and adverse psychosocial outcomes, results from a twin study. Arch Gen Psychiatry, 59(2), 139-45.

Nelson, E. C., Heath, A. C., Lynskey, M. T., Bucholz, K. K., Madden, P. A. F., Statham, D. J., & Martin, N.G. (2006). Childhood sexual abuse and risks for licit and illicit drug-related outcomes: A twin study. Psychological Medicine, 36, 1473-1483.

Nemeroff, C.B., Heim, C.M., Thase, M.E., Klein, D.N., Rush, A.J. et al. (2003). Differential responses to psychotherapy versus pharmacotherapy in patients with chronic forms of major depression and childhood trauma. Proc Natl Acad Sci USA, 100(24), 14293-6.

Nguyen, H.T., Dunne, M.P. & Le, A.V. (2009). Multiple types of child maltreatment and adolescent mental health in Vietnam. Bulletin of the World Health Organization, 87, 22-30.

Nichols, H.B., & Harlow, B.L (2004). Childhood abuse and risk of smoking onset. J Epidemiol Community Health, 58, 402–406.

Nickel, M. K., Tritt, K., Mitterlehner, F. O., Leiberich, P., Nickel, C. et al. (2004). Sexual abuse in childhood and youth as psychopathologically relevant life occurrence crosssectional survey. Croatian Medical Journal, 45, 483-489.

Nicolaidis, C., Curry, M., Mcfarland, B.H., & Gerrity, M.S. (2004). Violence, mental health, and physical symptoms in an academic internal medicine practice. J Gen Intern Med, 19(8), 819-27.

Nicolaidis, C., mcfarland, B.H., Curry, M., & Gerrity, M.S. (2009). Differences in physical and mental health symptoms and mental health utilization associated with intimate-partner violence versus childhood abuse. Psychosomatics, 50(4), 340-6.

Niederberger, J.M. (2002). The perpetrator's strategy as a crucial variable, a representative study of sexual abuse of girls and its sequelae in Switzerland. Child Abuse and Neglect, 26, 55−71.

Oaksford, K. L., & Frude, N. (2001). The prevalence and nature of child sexual abuse: Evidence from a female university sample in the UK. Child Abuse Review, 10, 49−59.

O'Leary, A., Purcell, D., Remien, R.H., & Gomez, C. (2003). Childhood sexual abuse and sexual transmission risk behaviour among HIV-positive men who have sex with men. AIDS Care, 15(1), 17-26.

Olsson, A., Ellsberg, M., Berglund, S., Herrera, A., Zelaya, E. et al. (2000). Sexual abuse during childhood and adolescence among Nicaraguan men and women: A population-based anonymous survey. Child Abuse and Neglect, 24, 1579-1589.

Oquendo, M., Brent, D.A., Birmaher, B., Greenhill, L, Kolko, D. et al. (2005). Posttraumatic Stress Disorder Comorbid With Major Depression: Factors Mediating the Association With Suicidal Behavior. Am J Psychiatry, 162, 560-566.

Orozco, R., Borges, G., Benjet, C., Medina-Mora, M.E., & Lopez-Carrillo L. (2008). Traumatic life events and posttraumatic stress disorder among Mexican adolescents, results from a survey. Salud Publica Mex, 50, 29–37

Parillo, K., Freeman, R., Collier, K., & Young, P. (2001). Association between early sexual abuse and adult HIV-risky sexual behaviors among community-recruited women. Child Abuse and Neglect, 25, 335–346.

Paul, J. P., Catania, J., Pollack, L., & Stall, R. (2001). Understanding childhood sexual abuse as a predictor of sexual risk-taking among men who have sex with men, The Urban Men’s Health Study.

Child Abuse and Neglect, 25, 557-584.

Paivio, S.C., & Cramer, K.M. Factor structure and reliability of the Childhood Trauma Questionnaire in a Canadian undergraduate student sample. Child Abuse and Neglect. 2004 Aug, 28(8), 889-904.

Pereda, N., Abad, J., & Guilera, G. (2016). Lifetime Prevalence and Characteristics of Child Sexual Victimization in a Community Sample of Spanish Adolescents. J Child Sex Abus, 25(2), 142-58.

Pérez-Fuentes, G., Olfson, M., Villegas, L., Morcillo, C., Wang, S., & Blanco, C. (2013). Prevalence and correlates of child sexual abuse, a national study. Compr Psychiatry, 54(1), 16-27.

Peschers, U. M., Du Mont, J., Jundt, K., Pfurtner, M., Dugan, E., & Kindermann, G. (2003). Prevalence of sexual abuse among women seeking gynecologic care in Germany. The American College of Obstetricians and Gynecologists, 101, 103-108.

Pluck, G., Lee, K.W., David, R., Macleod, D.C., Spence, S.A. et al (2011). Neurobehavioural and cognitive function is linked to childhood trauma in homeless adults. Brit J Clin Psychol 50, 33–45

Priebe, G., & Svedin, C.G. (2009). Prevalence, characteristics, and associations of sexual abuse with sociodemographics and consensual sex in a population-based sample of Swedish adolescents.

Journal of Child Sexual Abuse, 18, 19–39.

Rada, C. (2014). Violence against women by male partners and against children within the family, prevalence, associated factors, and intergenerational transmission in Romania, a cross-sectional study. B.MC Public Health, 14, 129.

Radford, L., Corral, S., Bradley, C., Fisher, H., Bassett, C. et al. Child Abuse and Neglect, in the UK today. London, National Society for the Prevention to Cruelty to Children, 2011.

Ramiro, L.S., Madrid, B.J., & Brown, D.W (2010). Adverse childhood experiences (ACE) and health-risk behaviors among adults in a developing country setting. Child Abuse and Neglect, 34, 842–855.

Ramos, B.M., Carlson, B.E., & Mcnutt, L. (2004). Lifetime abuse, mental health, and African American women. J Fam Violence, 19(3), 153-64.

Rayburn, N.R., Wenzel, S.L., Elliott, M.N., Hambarsoomians, K., Marshall, J.N. et al (2005). Trauma, depression, coping, and mental health service seeking among impoverished women. J Consult Clin Psychol, 73, 667–677

Rich-Edwards, J.W., Spiegelman, D., Hibert, E.NL, Jun H-J., Todd, T.J. et al. (2010). Abuse in childhood and adolescence as a predictor of type 2 diabetes in adult women. Am J Prev Med, 39, 529–536.

Riley, E.H., Wright, R.J., Jun, H.J., Hibert, E.N., & Rich-Edwards, J.W (2010). Hypertension in adult survivors of child abuse, observations from the Nurses’ Health Study II. J Epidemiol Community Health, 64, 413–418.

Ritchie, K., Jaussent, I., Stewart, R., Dupuy, A.M., Courtet, P. et al. (2009). Association of adverse childhood environment and 5-H.TTLPR genotype with late-life depression. J Clin Psychiatry, 70(9), 1281-88.

Roberts, A. L., Glymour, M. M., & Koenen, K.C. (2013). Does maltreatment in childhood affect sexual orientation in adulthood? Archives of Sex Behavior, 42, 161–171.

Robohm, J. S., Litzenberger, B. W., & Pearlman, L.A. (2003). Sexual abuse in lesbian and bisexual young women, Associations with emotional/behavioral difficulties, feelings about sexuality, and the

‘coming out’ process. Journal of Lesbian Studies, 7, 31-47.

Rohde, P., Ichikawa, L., Simon, G.E., Ludman, E.J., Linde, J.A. et al. (2008). Associations of child sexual and physical abuse with obesity and depression in middle-aged women. Child Abuse and Neglect, 32(9), 878-87

Romans, S., Belaise, C., Martin, J., Morris, E., & Raffi A (2002). Childhood abuse and later medical disorders in women, an epidemiological study. Psychother Psychosom, 71, 141–150.

Rosenberg, H. J., Jankowski, M. K., Sengupta, A., Wolfe, R. S., Wolford, G. L., & Rosenberg, S.D. (2005). Single and multiple suicide attempts and associated health risk factors in New Hampshire

Adolescents. Suicide and Life-Threatening Behavior, 35, 547-557.

Rosenman, S., & Rodgers, B. (2004). Childhood adversity in an Australian population. Social Psychiatry and Psychiatric Epidemiology, 39, 695-702.

Ross, C. A., Keyes, B. B., Xiao, Z., Yan, H., Wang, Z. et al. (2005). Childhood physical and sexual abuse in China. Journal of Child Sexual Abuse, 14, 115-126.

Runtz, M. (2002). Health concerns of university women with a history of child physical and sexual maltreatment. Child Maltreatment, 7, 241-253.

Sar, V., Akyüz, G., Kundakçi, T., Kiziltan, E., & Dogan, O. (2004). Childhood trauma, dissociation, and psychiatric comorbidity in patients with conversion disorder. Am J Psychiatry, 161(12), 2271-6.

Sar, V., Akyüz, G., Öztürk, E., & Alioğlu, F. (2013). Dissociative depression among women in the community. J Trauma Dissociation, 14(4), 423-38.

Schein, M., Biderman, A., Baras, M., Bennett, L., Bisharat, B. et al. (2000). The prevalence of a history of child sexual abuse among adults visiting family practitioners in Israel. Child Abuse and Neglect, 24(5), 667−675.

Scher, C.D., Forde, D.R., Mcquaid, J.R., & Stein, M.B. (2004). Prevalence and demographic correlates of childhood maltreatment in an adult community sample. Child Abuse and Neglect, 28(2), 167-80.

Schoemaker, C., Smit, F., Bijl, R.V., &Vollebergh, W.A.M. (2002). Bulimia nervosa following psychological and multiple child abuse, support for the self medication hypothesis in a population-based cohort study. Int J Eat Disord, 32, 381–388.

Schultz, J. R., Bell, K. M., Naugle, A. E., & Polusny, M.A. (2006). Child sexual abuse and adulthood sexual assault among military veteran and civilian women. Military Medicine, 171, 723-728.

Scott, K.M., Von Korff, M., Alonso, J., Angermeyer, M.C., Benjet, C. et al. (2008). Childhood adversity, early-onset depressive/anxiety disorders, and adult-onset asthma. Psychosom Med, 70, 1035–1043.

Seedat, S., Nyamai, C., Njenga, F., Vythilingum, B., & Stein, D.J. (2004). Trauma exposure and post-traumatic stress symptoms in urban African schools. Survey in capetown and Nairobi. Br J Psychiatry, 184, 169-75.

Sesar, K., Zivcic-Becirevic, I., & Sesar, D. (2008). Multi-type maltreatment in childhood and psychological adjustment in adolescence, questionnaire study among adolescents in Western Herzegovina Canton. Croat Med J, 49, 243–256.

Shen, A.C.T. (2008). Self-esteem of young adults experiencing interparental violence and child physical maltreatment, parental and peer relationships as mediators. Journal of Interpersonal Violence, 24(5), 770-794.

Shen, A.C.T. (2009). Long-term effects of interparental violence and child physical maltreatment experiences on PTSD and behavior problems: A national survey of Taiwanese college students. Child Abuse and Neglect, 33, 148–160.

Silvern, L., Waelde, L. C., mcclintic Baughan, B., & Kaersvang, L.L. (2000). Two formats for eliciting retrospective reports of child sexual and physical abuse, Effects on apparent prevalence and relationships to adjustment. Child Maltreatment, 5, 236-250.

Slonim-Nevo, V., & Mukuka, L. (2007). Child abuse and aids related knowledge, attitudes and behavior among adolescents in Zambia. Child Abuse and Neglect, 31, 143-159.

So-kum Tang, C. (2002). Childhood experience of sexual abuse among Hong Kong Chinese college students. Child Abuse and Neglect, 26, 23.

Sørbø, M.F., Grimstad, H., Bjørngaard, J.H., Schei, B., & Lukasse, M. (2013). Prevalence of sexual, physical and emotional abuse in the Norwegian mother and child cohort study. BMC Public Health, 13, 186.

Spertus, I.L., Yehuda, R., Wong, C.M., Halligan, S.L., & Seremetis, S.V. (2003). Childhood emotional abuse and neglect as predictors of psychological and physical symptoms in women presenting to a primary care practice. Child Abuse and Neglect, 27(11), 1247-58.

Springer, K.W. (2009). Childhood physical abuse and midlife physical health: Testing a multi-pathway life course model. Soc Sci Med, 69(1), 138-46.

Springer, K.W., Sheridan, J., Kuo, D., & Carnes, M. (2007). Long-term physical and mental health consequences of childhood physical abuse, results from a large population-based sample of men and women. Child Abuse and Neglect, 31, 517–530.

Steel, J. L., & Herlitz, C. A. (2005). The association between childhood and adolescent sexual abuse and proxies for sexual risk behaviour: A random sample of the general population of Sweden. Child

Abuse & Neglect, 29, 1141-1153.

Stein, J.A., Leslie, M.B., & Nyamathi, A. (2002). Relative contributions of parent substance use and childhood maltreatment to chronic homelessness. Child Abuse and Neglect, 26, 1011–1027

Stenson, K., Heimer, G., Lundh, C., Nordstro¨m, M. L., Saarinen, H., & Wenker, A. (2003). Lifetime prevalence of sexual abuse in a Swedish pregnant population. Acta Obstetetrica et Gynecologica

Scandinavica, 82, 529-536.

Stephenson, R., Sheikhattari, P., Assasi, N., Eftekhar, H., Zamani, Q. et al. (2006). Child maltreatment among school children in the Kurdistan Province, Iran. Child Abuse and Neglect, 30, 231–245.

Stoddard, J. P., Dibble, S. L., & Fineman, N. (2009). Sexual and physical abuse: A comparison between lesbians and their heterosexual sisters. Journal of Homosexuality, 56, 407-420.

Strine, T.W., Dube, S.R., Edwards, V.J., Prehn, A.W., Rasmussen, S. et al. (2012). Associations between adverse childhood experiences, psychological distress, and adult alcohol problems. Am J Health Behav, 36, 408–423.

Subica, A.M. (2013). Psychiatric and physical sequelae of childhood physical and sexual abuse and forced sexual trauma among individuals with serious mental illness. J Trauma Stress, 26(5), 588-96.

Suija, K., Aluoja, A., Kalda, R., & Maaroos, H.I. (2011). Factors associated with recurrent depression, a prospective study in family practice. Fam Prac, 28, 22–28

Sun, Y.P., Zhang, B., Dong, Z.J., Yi, M.J., Sun, D.F. & Shi, S.S. (2008). Psychiatric state of college students with a history of child sexual abuse. World Journal of Pediatrics, 4(4), 2898-294.

Sun, N., Li, Y., Cai, Y., Chen, J., Shen, Y. et al. (2012). A comparison of melancholic and nonmelancholic recurrent major depression in Han Chinese women. Depress Anxiety, 29(1), 4-9.

Sung, S.C., Wisniewski, S.R., Balasubramani, G.K., Zisook, S., Kurian, B. et al. (2013). Does early-onset chronic or recurrent major depression impact outcomes with antidepressant medications? A CO-MED trial report. Psychol Med, 43(5), 945-60.

Swahn, M.H., & Bossarte, R.M. (2007). Gender, early alcohol use, and suicide ideation and attempts, findings from the 2005 youth risk behavior survey. J Adolesc Health, 41(2), 175-81.

Tang, C.S. (2002).Childhood experiences of sexual abuse among Hong Kong Chinese College students. Child Abuse and Neglect, 26, 23−37.

Thakkar, R. R., Gutierrez, P. M., Kuczen, C. L., & Mccanne, T. R. (2000). History of physical and/or sexual abuse and current suicidality in college women. Child Abuse and Neglect, 24, 1345-1354.

The NIMH Multisite HIV Prevention Trial Group. (2001). A test of factors mediating the relationship between unwanted sexual activity during childhood and risky sexual practices among women enrolled in the NIMH Multisite HIV Prevention Trial. Women & Health, 33, 163–180.

Thompson, M. P., Kaslow, N. J., Bradshaw, D., Lane, D. B., & Kingree, J.B. (2000). Childhood maltreatment, PTSD and suicidal behavior among African American females. Journal of Interpersonal Violence, 15, 3–15.

Thompson, M. P., Arias, I., Basil, C., & Desai, S. (2002). The association between childhood physical and sexual victimization and health problems in adulthood in a nationally representative sample

Of women. Journal of Interpersonal Violence, 17, 1115-1128.

Thompson, M.P., Kingree, J.B., & Desai, S. (2004). Gender differences in long-term health consequences of physical abuse of children, data from a nationally representative survey. Am J Public Health, 94, 599–604.

Thurman, T.R., Brown, L., Richter, L., Maharaj, P., & Magnani, R. (2006). Sexual risk behavior among South African adolescents, is orphan status a factor? AIDS Behav, 10, 627–635.

Tietjen, G.E., Brandes, J.L., Peterlin, B., Eloff, A., Dafer, R.M. et al. (2010). Childhood maltreatment and migraine (Part I): Prevalence and adult revictimization, a multicenter headache clinic survey.

Headache, 50(1), 20-31.

Timko, C., Sutkowi, A., Pavao, J., & Kimerling, R. (2008). Women’s childhood and adult adverse experiences, mental health, and binge drinking, the California Women’s Health Survey. Subst Abuse Treat Prev Policy, 3, 15.

Tomeo, M. E., Templer, D. I., Anderson, S., & Kotler, D. (2001). Comparative data of childhood and adolescence molestation in heterosexual and homosexual persons. Archives of Sexual Behavior,

30, 535-541.

Tourigny, M., Hébert, M., Joly, J., Cyr, M., & Baril, K. (2008). Prevalence and co-occurrence of violence against children in the Quebec population. Aust N Z J Public Health, 32(4), 331-5.

Tran, Q.A., Dunne, M.P., Vo, T.V., & Luu, N.H. (2015). Adverse childhood experiences and the health of university students in eight provinces of Vietnam. Asia Pac J Public Health, 27, 26S-32S.

Tran, Q.A. Link, L.R.A.A., Van Berkel, S.R., & Van Ijzendoorn, M.H. (2017). Child Maltreatment in Vietnam, prevalence and cross-cultural comparison. Journal of Aggression, Maltreatment & Trauma, 26(3), 211-230.

Trent, L., Stander, V., Thomsen, C., & Merrill, L. (2007). Alcohol abuse among US Navy recruits who were maltreated in childhood. Alcohol Alcohol, 42, 370–375.

Van der Kooij, I.W., Nieuwendam, J., Bipat, S., Boer, F., Lindauer, R.J., & Graafsma, T.L. (2015). A national study on the prevalence of child abuse and neglect in Suriname. Child Abuse and Neglect, 47, 153-61.

Vander Weg, M.W (2011). Adverse childhood experiences and cigarette smoking, the 2009 Arkansas and Louisiana Behavioral Risk Factor Surveillance Systems. Nicotine Tob Res, 13, 616–622.

Von Korff, M., Alonso, J., Ormel, J., Angermeyer, M., Bruffaerts, R. et al. (2009). Childhood psychosocial stressors and adult onset arthritis, broad spectrum risk factors and allostatic load. Pain, 143, 76–83.

Wainwright, N.J., & Surtees, P.G. (2002). Childhood adversity, gender and depression over the life-course. J Affect Disord, 72(1), 33-44.

Wan, G.WY, & Leung, P.W.L. (2010). Factors accounting for youth suicide attempt in Hong Kong, a model building. J Adolesc, 33, 575–582.

Welles, S. L., Baker, A. C., Miner, M. H., Brennan, D. J., Jacoby, S., & Rosser, B.R. (2009). History of childhood sexual abuse and unsafe anal intercourse in a 6-city study of HIV-positive men who have sex with men. American Journal of Public Health, 99, 1079–1086.

Wiersma, J.E., Hovens, J.M., van Oppen, P., Giltay, E.J., van Schaik, D.J. et al. (2009). The importance of childhood trauma and childhood life events for chronicity of depression in adults. J Clin Psychiatry, 70(7), 983-9.

Wilhelm, K., Roy, K., Mitchell, P., Brownhill, S., & Parker, G. (2002). Gender differences in depression risk and coping factors in a clinical sample. Acta Psychiatr Scand, 106(1), 45-53.

Wilsnack, S. C., Hughes, T. L., Johnson, T. P., Bostwick, W. B., Szalacha, L. A. et al. (2008). Drinking and drinking-related problems among heterosexual and sexual minority women. Journal of Studies on Alcohol & Drugs, 69, 129-139.

Wilsnack, S. C., Kristjanson, A. F., Hughes, T. L., & Benson, P. W. (2012). Characteristics of childhood sexual abuse in lesbians and heterosexual women. Child Abuse and Neglect, 36, 260–265.

Wise, L.A., Zierler, S., Krieger, N., & Harlow, B.L. (2001). Adult onset of major depressive disorder in relation to early life violent victimisation: A case-control study. Lancet, 358(9285), 881-7.

Wise, L.A., Palmer, J.R., Boggs, D.A., Adams-Campbell, L.L., & Rosenberg, L. (2011). Abuse victimization and risk of breast cancer in the Black Women’s Health Study [corrected]. Cancer Causes Control, 22, 659–669.

Yen, C.F., Yang, M.S., Yang, M.J., Su, Y.C., Wang, M.H., & Lan, C.M. (2008). Childhood physical and sexual abuse, Prevalence and correlates among adolescents living in rural Taiwan. Child Abuse and Neglect, 32(3), 429-38.

Yen, C.F., Yang, M.S., Chen, C.C., Yang, M.J., Su, Y.C., Wang, M.H. & Lan, C.M. (2008)(b). Effects of childhood physical abuse on depression, problem drinking and perceived poor health status in adolescents living in rural Taiwan. Psychiatry and Clinical Neurosciences, 62, 575-583.

Yoshihama, M. & Horrocks, J. (2010). Risk of intimate partner violence: Role of childhood sexual abuse and sexual initiation in women in Japan. Children and Youth Services Review, 32, 28-37.

Yoshinaga, C., Kadomoto, I., Otani, T., Sasaki, T., & Kato, N. (2004). Prevalence of post-traumatic stress disorder in incarcerated juvenile delinquents in Japan. Psychiatry and Clinical Neurosciences, 58, 383-388.

Young, S.YN, Hansen, C.J., Gibson, R.L., Ryan, M.A.K. (2006). Risky alcohol use, age at onset of drinking, and adverse childhood experiences in young men entering the US Marine Corps. Arch

Pediatr Adolesc Med, 160, 1207–1214.

Young, A., Grey, M., Abbey, A., Boyd, C.J., & Mccabe, S.E. (2008). Alcohol-related sexual assault victimization among adolescents, prevalence, characteristics, and correlates. J Stud Alcohol Drugs, 69(1), 39-48.

Ystgaard, M., Hestetun, I., Loeb, M., & Mehlum, L. (2004). Is there a specific relationship between childhood sexual and physical abuse and repeated suicidal behavior? Child Abuse and Neglect, 28, 863–75.

Zanarini, M.C., Yong, L.MA, Frankenburg, F.R., Hennen, J., Reich, D.B. et al. (2002). Severity of reported childhood sexual abuse and its relation to severity of borderline psychopathology and psychosocial impairment among borderline inpatients. J Nerv Ment Dis, 190, 381–7.

Zhao, Q., Zhao, J., Li, X., Zhao, G., Fang, X. et al. (2010). Childhood sexual abuse and its relationship with psychosocial outcomes among children affected by HIV in rural China. Journal of the Association of Nurses in AIDS Care, 22(3), 202-14

Zlotnick, C., Mattia, J.I., & Zimmerman, M. (2001). Clinical features of survivors of sexual abuse with major depression. Child Abuse and Neglect, 25(3), 357-67.

Zoroglu, S.S., Tuzun, U., Sar, V., Tutkun, H., Savas, H.A. et al. (2003). Suicide attempt and self-mutilation among Turkish high school students in relation with abuse, neglect, and dissociation. Psychiatry Clin Neurosci, 57, 119–26.
