# Supplementary material for: Establishing the international prevalence of self-reported child maltreatment: a systematic review by maltreatment type and gender
Source: BMC Public Health. 2018 Oct 10;18:1164. doi: 10.1186/s12889-018-6044-y (PMC6180456; doi:10.1186/s12889-018-6044-y)
Supplement: Supplementary file 4 — Prevalence of abuse by type and population. (DOCX 28 kb) [file 12889_2018_6044_MOESM4_ESM.docx]

**Prevalence of abuse by type and population**

**
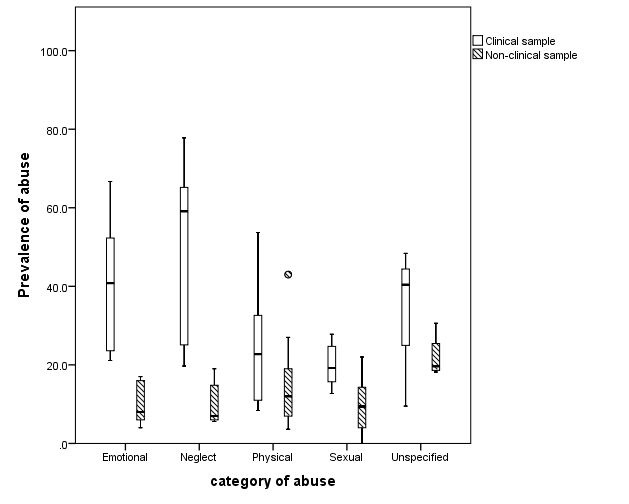
**

|  | **Emotional** | **Neglect** | **Physical** | **Sexual** | **Unspecified** |
| --- | --- | --- | --- | --- | --- |
| **Clinical sample** |  |  |  |  |  |
| N studies | 6 | 5 | 6 | 5 | 3 |
| Median  (25th to 75th centile) | 40.8  (23.6 to 52.3) | 59.1  (25.1 to 65.2) | 22.8  (11.0 to 32.6) | 19.2  (15.7 to 24.7) | 40.4  (9.5 to 48.4) |
| **Non-clinical sample** |  |  |  |  |  |
| N studies | 10 | 9 | 15 | 14 | 4 |
| Median  (25th to 75th centile) | 8.0  (6.0 to 16.0) | 7.0  (6.0 to 14.8) | 12.0  (6.9 to 23.0) | 9.4  (4.0 to 14.3) | 19.7  (18.6 to 25.5) |
