# Supplementary material for: Establishing the international prevalence of self-reported child maltreatment: a systematic review by maltreatment type and gender
Source: BMC Public Health. 2018 Oct 10;18:1164. doi: 10.1186/s12889-018-6044-y (PMC6180456; doi:10.1186/s12889-018-6044-y)
Supplement: Supplementary file 5 — Prevalence of maltretment by continent and gender - non-clinical samples only. (DOCX 81 kb) [file 12889_2018_6044_MOESM5_ESM.docx]

**NON-CLINICAL SAMPLES ONLY**

**Prevalence of emotional abuse by continent and gender - non-clinical sample only**


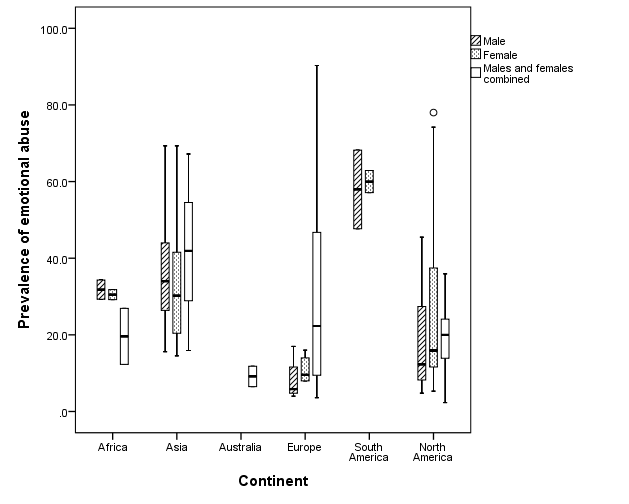


o = Outliers. Between 1.5 and 3 times the height of the boxes (25^th^ to 75^th^ centile)

* = Extreme outliers. Values more than 3 times the height of the boxes (25^th^ to 75^th^ centile)

|  | **Africa** | **Asia** | **Australia** | **Europe** | **S**  **America** | **N**  **America** |
| --- | --- | --- | --- | --- | --- | --- |
| **Males** | | | | | | |
| N studies | 2 | 9 | 0 | 4 | 2 | 13 |
| Median (25th to 75th centile) | 31.8  (29.3 to 34.3) | 34.0  (26.4 to 44.0) | - | 5.9  (4.8 to 11.6) | 58.0  (47.7 to 68.2) | 12.3  (8.2 to 27.4) |
| **Females** | | | | | | |
| N studies | 2 | 12 | 0 | 5 | 2 | 19 |
| Median (25th to 75th centile) | 30.5  (29.2 to 31.8) | 30.3  (20.4 to 41.6) | - | 9.6  (8.0 to 14.0) | 60.0  (57.1 to 62.9) | 15.9  (11.5 to 37.5) |
|  | | | | | | |
| N studies | 2 | 3 | 2 | 11 | 0 | 10 |
| Median (25th to 75th centile) | 19.6  (12.3 to 26.9) | 41.9  (15.9 to 67.2) | 9.2 (6.5 to 11.8) | 22.3  (6.0 to 51.2) | - | 20.0  (13.9 to 24.1) |

**Prevalence of neglect by continent and gender - non-clinical sample only**

**
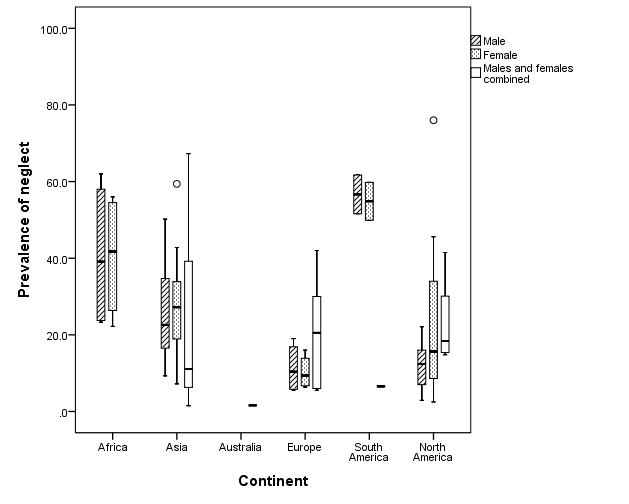
**

o = Outliers. Between 1.5 and 3 times the height of the boxes (25^th^ to 75^th^ centile)

* = Extreme outliers. Values more than 3 times the height of the boxes (25^th^ to 75^th^ centile)

|  | **Africa** | **Asia** | **Australia** | **Europe** | **S**  **America** | **N**  **America** |
| --- | --- | --- | --- | --- | --- | --- |
| **Males** | | | | | | |
| N studies | 4 | 7 | 0 | 4 | 2 | 5 |
| Median  (25th to 75th centile) | 39.1  (23.8 to 58.0) | 22.6  (12.9 to 44.5) | - | 10.4  (5.8 to 16.9) | 56.7  (51.6 to 61.7) | 12.4  (7.0 to 16.0) |
| **Females** | | | | | | |
| N studies | 4 | 9 | 0 | 4 | 2 | 8 |
| Median  (25th to 75th centile) | 41.8  (26.4 to 54.5) | 27.2  (18.9 to 33.9) | - | 9.4  (6.7 to 13.9) | 54.8  (49.9 to 59.8) | 15.6  (8.6 to 34.0) |
| **Combined** | | | | | | |
| N studies | 0 | 3 | 1 | 6 | 2 | 151 |
| Median  (25th to 75th centile) | - | 11.1  (31.5 to 67.3) | 1.6 | 20.5  (6.0 to 30.0) | 6.6  (6.5 to 6.6) | 18.4  (15.4 to 30.1) |

**Prevalence of sexual abuse by continent and gender - non-clinical sample only**

**
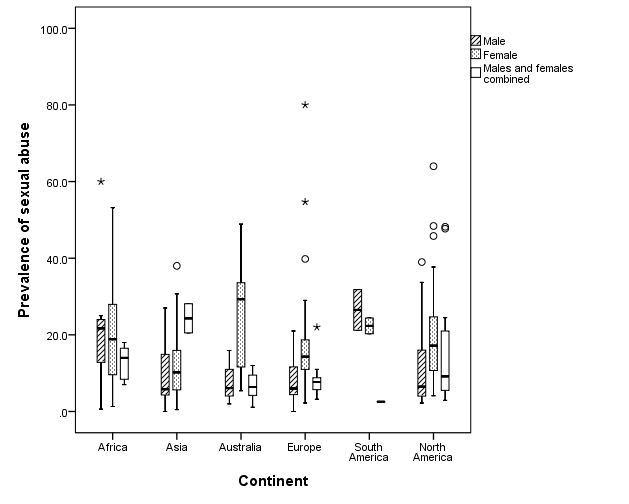
**

o = Outliers. Between 1.5 and 3 times the height of the boxes (25^th^ to 75^th^ centile)

* = Extreme outliers. Values more than 3 times the height of the boxes (25^th^ to 75^th^ centile)

|  | **Africa** | **Asia** | **Australia** | **Europe** | **S**  **America** | **N**  **America** |
| --- | --- | --- | --- | --- | --- | --- |
| **Males** | | | | | | |
| N studies | 9 | 18 | 3 | 16 | 2 | 34 |
| Median  (25th to 75th centile) | 21.7  (12.8 to 24.0) | 5.8  (4.3 to 14.9) | 6.1  (2.0 to 15.9) | 6.0  (4.4 to 11.7) | 26.5  (21.2 to 31.8) | 6.5  (4.0 to 16.0) |
| **Females** | | | | | | |
| N studies | 12 | 35 | 6 | 21 | 2 | 61 |
| Median  (25th to 75th centile) | 18.9  (9.6 to 28.0) | 10.2  (5.3 to 16.7) | 29.3  (11.6 to 33.6) | 14.3  (11.0 to 18.7) | 22.4  (20.3 to 24.4) | 17.2  (10.7 to 24.7) |
| **Combined** | | | | | | |
| N studies | 5 | 2 | 5 | 9 | 2 | 18 |
| Median  (25th to 75th centile) | 13.0  (8.4 to 16.5) | 24.3  (20.5 to 28.1) | 6.4  (4.2 to 9.5) | 7.7  (5.7 to 8.8) | 2.6  (2.5 to 2.6) | 9.2  (5.5 to 21.0) |

**Prevalence of physical abuse by continent and gender – non-clinical sample only**

**
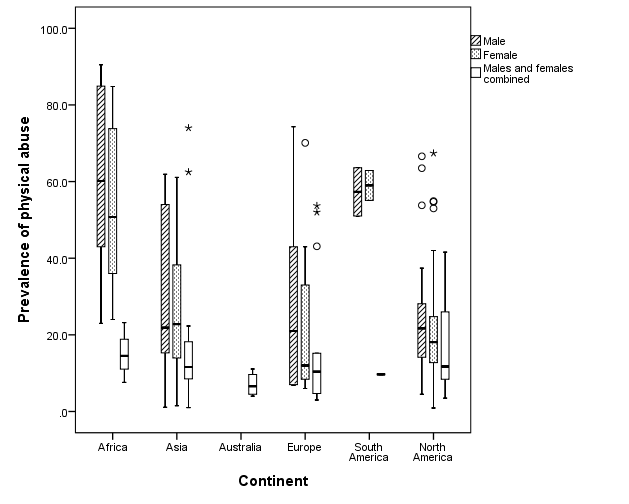
**

o = Outliers. Between 1.5 and 3 times the height of the boxes (25^th^ to 75^th^ centile)

* = Extreme outliers. Values more than 3 times the height of the boxes (25^th^ to 75^th^ centile)

|  | **Africa** | **Asia** | **Australia** | **Europe** | **S**  **America** | **N**  **America** |
| --- | --- | --- | --- | --- | --- | --- |
| **Males** | | | | | | |
| N studies | 6 | 13 | 0 | 6 | 2 | 28 |
| Median  (25th to 75th centile) | 60.2  (43.0 to 84.9) | 21.9  (15.3 to 54.0) | - | 21.0  (7.0 to 43.0) | 57.3  (51.0 to 63.6) | 21.7  (14.1 to 28.1) |
| **Females** | | | | | | |
| N studies | 6 | 16 | 0 | 8 | 2 | 44 |
| Median  (25th to 75th centile) | 50.8  (36.0 to 73.8) | 22.8  (14.0 to 38.3) | - | 12.0  (8.4 to 33.0) | 59.0  (55.1 to 62.9) | 18.1  (12.8 to 24.8) |
| **Combined** | | | | | | |
| N studies | 3 | 11 | 4 | 14 | 2 | 22 |
| Median  (25th to 75th centile) | 14.5  (7.6 to 23.2) | 11.6  (7.5 to 22.3) | 6.6  (4.5 to 9.6) | 10.4  (4.7 to 15.2) | 9.7  (9.6 to 9.8) | 11.8  (8.4 to 26.0) |
